# Field-enhanced graphene fiber arrays reveal serum metabolic alterations in pancreatic cancer

**DOI:** 10.1039/d6sc04783a

**Published:** 2026-08-07

**Authors:** Zhihao Ye, Haiyan Luo, Mengyang Song, Pei Ding, Chao Ding, Junyu Chen, Zongxiu Nie, Yuze Li

**Affiliations:** a School of Physics, Ningxia University Yinchuan 750021 China; b State Key Laboratory of High-Efficiency Utilization of Coal and Green Chemical Engineering, School of Chemistry and Chemical Engineering, Ningxia University Yinchuan 750021 China znie@iccas.ac.cn liyuze@nxu.edu.cn; c Zhengzhou University of Aeronautics Zhengzhou 450046 China; d People's Hospital of Ningxia Hui Autonomous Region Yinchuan 750021 China; e Jiangxi Province Key Laboratory of Immunology and Inflammation, Jiangxi Provincial Clinical Research Center for Laboratory Medicine, Department of Clinical Laboratory, The Second Affiliated Hospital, Jiangxi Medical College, Nanchang University Nanchang 330006 China junyu94@outlook.com; f State Key Laboratory of Tropic Ocean Engineering Materials and Materials Evaluation, School of Marine Science and Engineering, Hainan University Haikou 570228 China; g Beijing National Laboratory for Molecular Sciences, Key Laboratory of Analytical Chemistry for Living Biosystems, Institute of Chemistry, Chinese Academy of Sciences Beijing 100190 China

## Abstract

A laser desorption/ionization mass spectrometry (LDI-MS) method based on laser-induced graphene fibers (LIGF) is developed. LIGF are fabricated in one step *via* CO_2_ laser writing on a polyimide film and serve directly as an LDI substrate without modification. Its fibrous structure produces a strong field-enhancement effect in negative-ion mode, promoting deprotonation of acidic metabolites into stable [M–H]^−^ ions and enabling highly sensitive (fmol-level), highly salt-tolerant, low-background, and uniform detection (with a relative standard deviation of approximately 2.26%). Computer-controlled laser scanning allows rapid production of LIGF arrays for high-throughput metabolic fingerprinting. Applied to serum metabolomics of pancreatic cancer (PC), LIGF-LDI-MS analysis of 195 samples (101 PC patients and 94 age-matched controls) yields tentative identification of 109 metabolites, 28 of which show significant inter-group differences, revealing PC-associated metabolic reprogramming. A neural-network model trained on these profiles achieves 88% diagnostic accuracy in blind validation. This work offers an efficient, low-cost strategy for the non-invasive detection of PC and provides insights for designing application-specific, high-performance LDI substrates.

## Abstract 2

The fiber arrays enable high-throughput serum metabolic fingerprinting and achieve 88% diagnostic accuracy for pancreatic cancer through machine learning.

## Graphical abstract

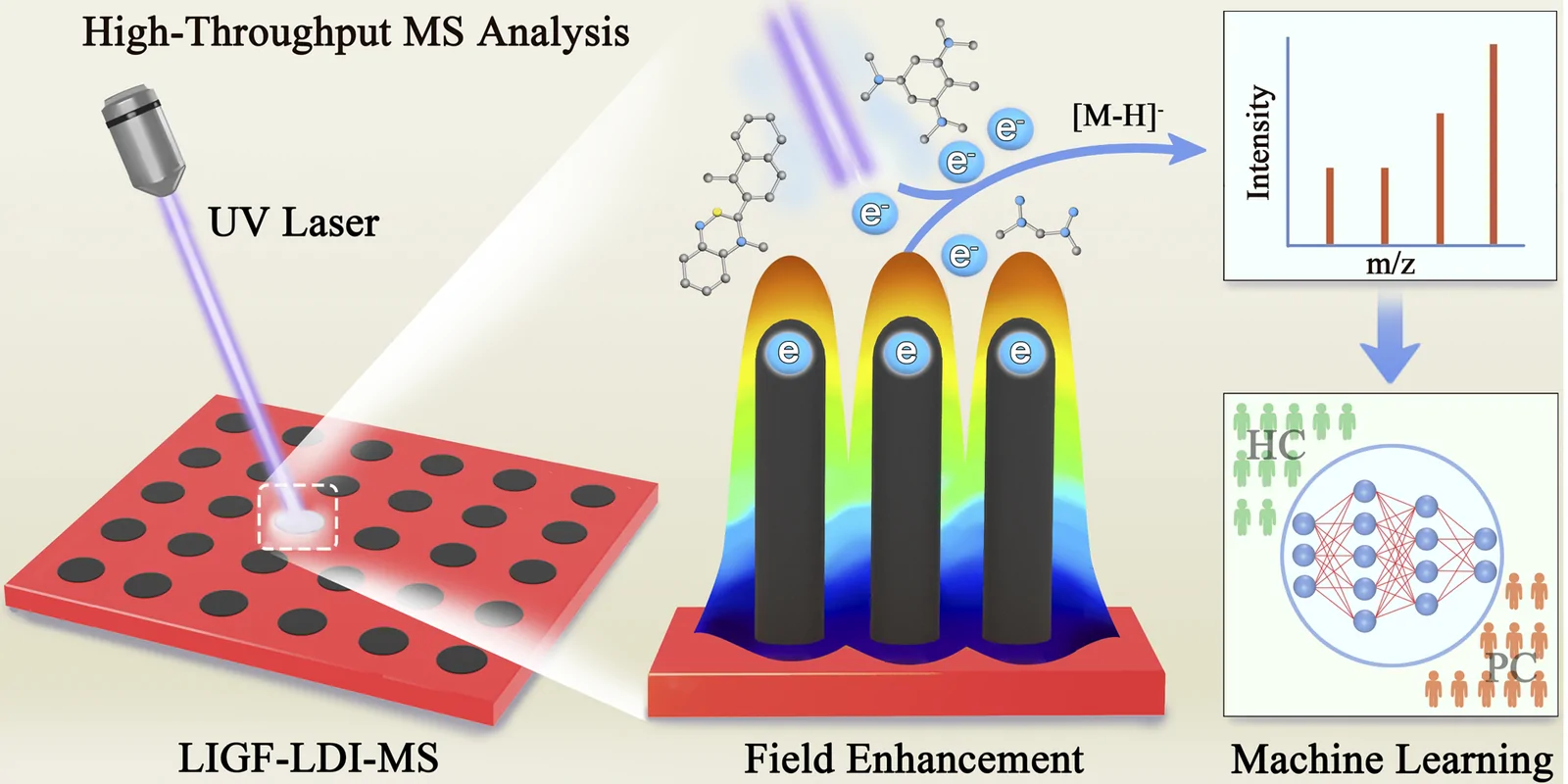

## 1 Introduction

PC is one of the most lethal malignancies worldwide, with a five-year survival rate of less than 10%.^1^ This dire prognosis is primarily attributed to its insidious symptoms and the lack of specific biomarkers, resulting in the majority of patients missing the opportunity for curative surgery at the time of diagnosis.^2,3^ Currently, clinical diagnosis of PC mainly relies on imaging examinations, including computed tomography (CT) and magnetic resonance imaging (MRI), as well as measurement of the serum carbohydrate antigen 19-9 (CA19-9). However, imaging modalities can exhibit limited specificity in definitively distinguishing malignant tumors from inflammatory conditions. Meanwhile, CA19-9, as the most commonly used serum biomarker, is not cancer-specific and is frequently elevated in various benign hepatobiliary and pancreatic disorders; its diagnostic specificity is therefore constrained, limiting its reliability for unambiguous diagnosis and monitoring.^4,5^ In recent years, mass spectrometry-based metabolomics has emerged as a promising strategy for cancer liquid biopsy.^6–10^ This technology enables the systematic profiling of global alterations in endogenous small-molecule metabolites within patient biofluids (*e.g.*, serum and plasma), unveiling disease-associated metabolic reprogramming and thereby facilitating the discovery of potential diagnostic biomarker panels.

Matrix-assisted laser desorption/ionization mass spectrometry (MALDI-MS) demonstrates distinctive advantages for rapid metabolic fingerprinting, owing to its straightforward sample preparation, high analytical speed, and minimal sample consumption.^11–14^ However, conventional organic compound matrices are prone to produce background interference in the low-mass region, and the mixing of matrix solution with samples tends to result in inhomogeneous crystallization, leading to the “sweet spot” effect. Consequently, considerable effort has been devoted to developing various techniques and methods to address the issues associated with traditional organic matrices.^15–21^ In recent years, the use of nanomaterials as LDI matrices or substrate materials has gradually become a primary approach to solving the challenges in small molecule analysis. Nanomaterials can lower the limit of detection (LOD) for analytes and exhibit higher signal intensity than conventional MALDI.^22,23^ Currently, the widely studied nanomaterial matrices or LDI substrate materials mainly include metals and their oxides,^24,25^ silicon-based nanomaterials,^26,27^ and carbon-based nanomaterials.^28,29^ However, most of these materials face challenges such as complex preparation processes, high production costs, susceptibility to contamination, and low ionization efficiency, which hinder their large-scale clinical application.

**Fig. 1:**
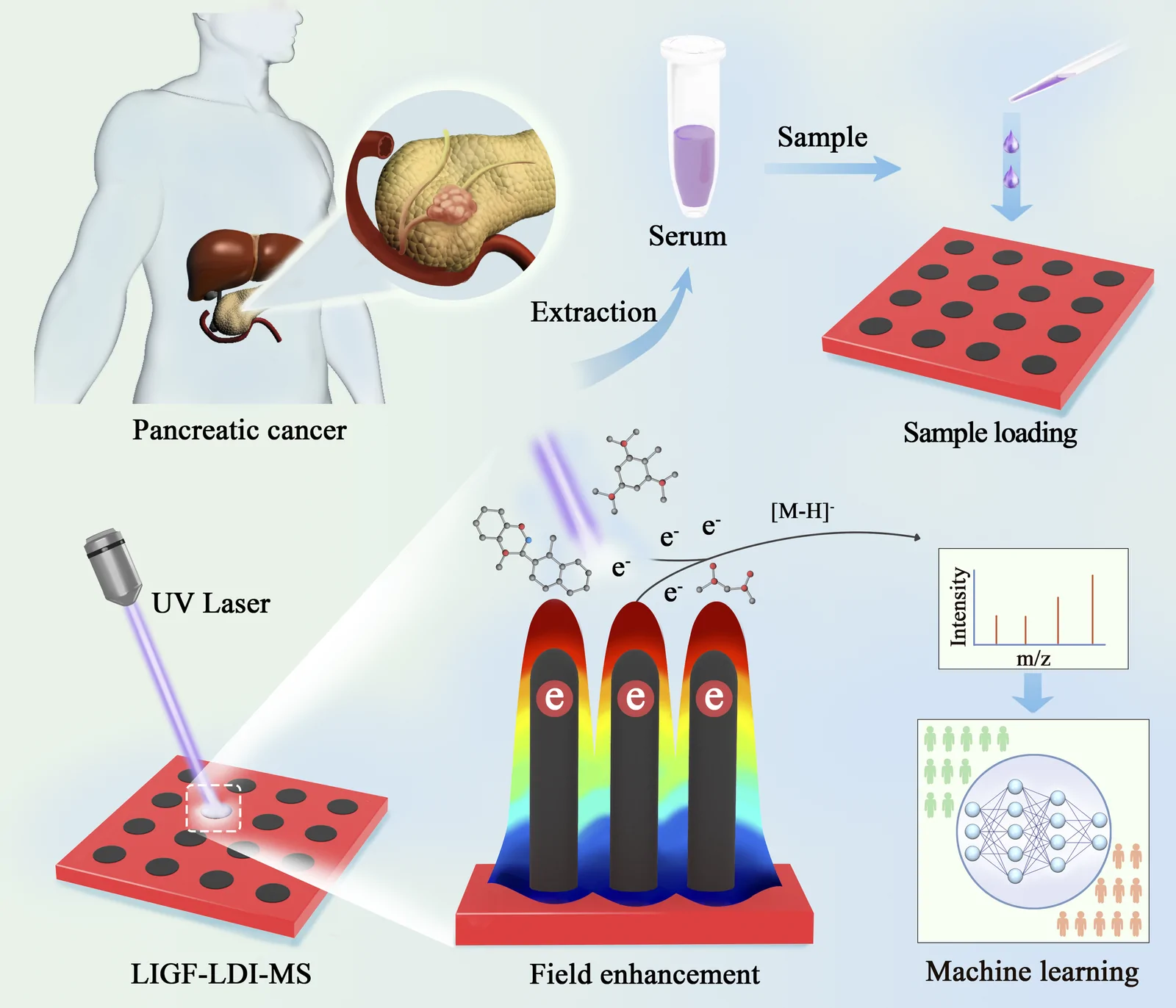
Workflow of the LIGF-LDI-MS technology for serum metabolomics in PC patients.

In this study, we propose the application of laser-induced graphene fibers (LIGF) as an LDI substrate in negative-ion mode. This approach enables the fabrication of LDI substrate materials using a single low-cost commercial laser engraver, with a streamlined process that significantly saves time and cost. Laser-induced graphene technology was first introduced for the rapid preparation of three-dimensional porous graphene films.^30^ Subsequent in-depth research revealed that increasing the laser power can significantly alter the morphology of graphene. At higher laser energy, graphene fibers can be induced on the surface of polyimide (PI) films.^31^ We applied the LIGF-LDI-MS technology to a serum metabolomics study of PC patients, with its workflow illustrated in Fig. 1.

## 2 Results and discussion

### 2.1. Material characterization

**Fig. 2:**
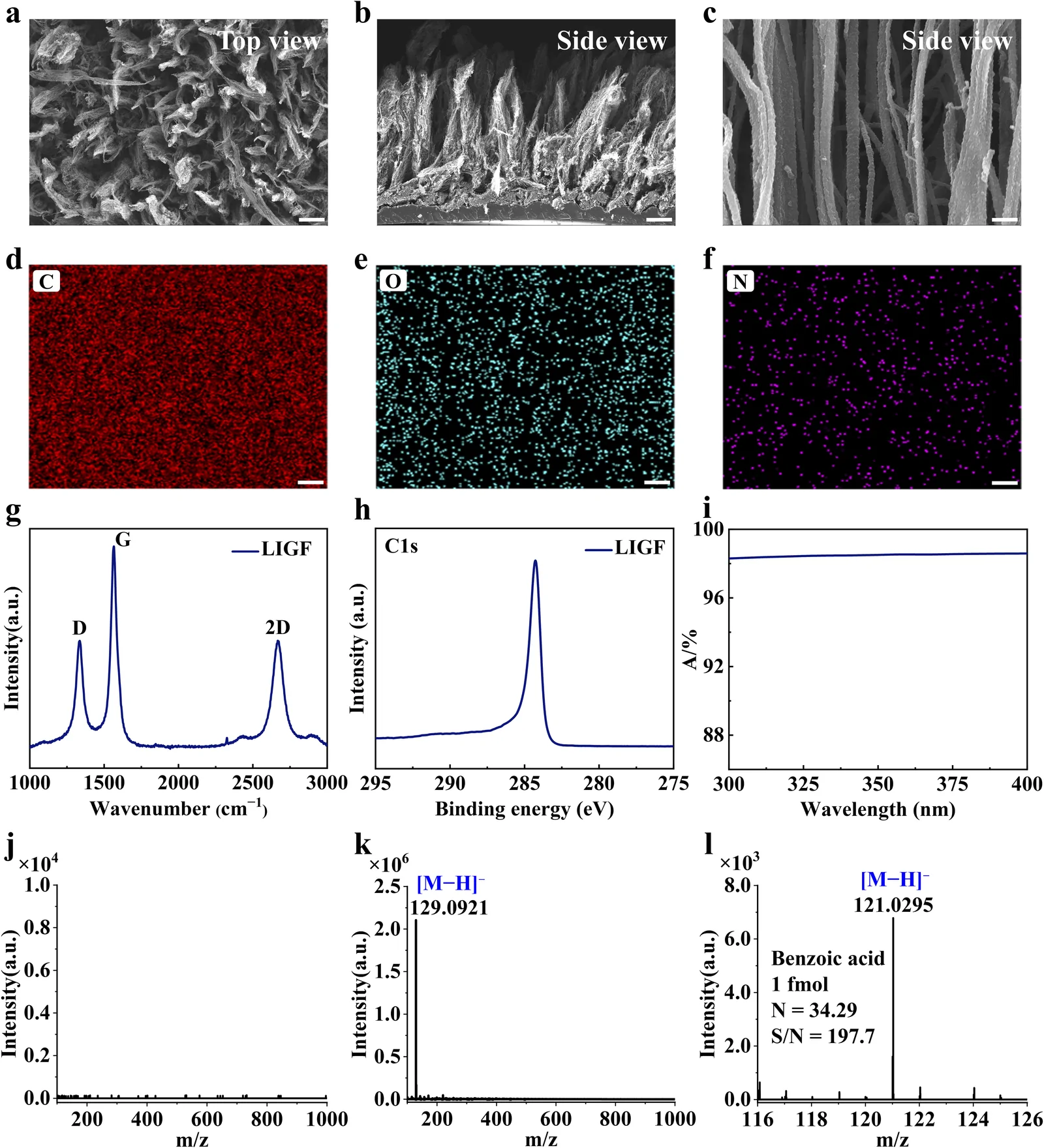
Characterization of LIGF. (a)–(c) SEM images of LIGF. Scale bars: 100 µm in (a) and (b), 500 nm in (c). (d)–(f) EDS elemental mapping of LIGF. Scale bar: 2.5 µm. (g) Raman spectrum, (h) C 1s XPS spectrum, (i) UV absorption spectrum, and (j) LDI-MS background spectrum of LIGF. LIGF-LDI-MS spectra of (k) 1 mg mL^−1^ heptanoic acid (1 µL) and (l) 1 fmol benzoic acid.

The LIG technique operates on the principle of applying laser radiation to a PI film, which generates intense localized heating. Under these conditions, specific chemical bonds in the polymer—such as C–O, C=O, and N–C—are broken. The nitrogen and oxygen atoms are subsequently eliminated as volatile gaseous products. Meanwhile, the remaining carbon atoms undergo structural reorganization, assembling into interconnected aromatic ring networks that yield three-dimensional porous graphene frameworks or fibrous graphene architectures.^30–32^ LIGF were fabricated by raster-scanning with a CO_2_ laser over the surface of a PI film (Fig. S1). We first optimized the laser power. As shown in Fig. S2, the SEM images of the graphene structures induced at laser powers of 5.0 W and 6.0 W both exhibited porous film-like morphologies. Upon further increasing the power to 7.0 W, fibrous structures were successfully obtained, as depicted in Fig. 2(a)–(c), which present the top view, side view, and a magnified side view of the LIGF, respectively. A typical fibrous microstructure is clearly observed in Fig. 2(c), with fiber heights of approximately 465 µm and diameters of approximately 245 nm. Furthermore, energy-dispersive X-ray spectroscopy (EDS) mapping was performed to analyze the elemental distribution on the LIGF surface, with the results displayed in Fig. 2(d)–(f). The atomic percentage (at%) of carbon was as high as 95.28% and uniformly distributed, indicating a high degree of carbonization of the PI film under the instantaneous high temperature induced by the laser (quantitative elemental results are provided in Table S1). To gain deeper insights into the structural characteristics of LIGF, X-ray photoelectron spectroscopy (XPS) and Raman spectroscopy were conducted. The C 1s peak in the XPS spectrum confirmed the sp^2^-hybridized structure of graphene (Fig. 2(h)).^33^ The Raman spectrum (Fig. 2(g)) exhibited three characteristic peaks of graphene: the D band (∼1350 cm^−1^, corresponding to structural defects), the G band (∼1580 cm^−1^, originating from the in-plane vibration of sp^2^ carbon atoms), and the 2D band (∼2700 cm^−1^, a second-order resonant peak related to the number of graphene layers), further confirming the successful induction of a graphene fiber structure on the PI surface.^34–36^ To characterize the quality uniformity of LIGF across different fabrication batches, representative Raman spectra of two additional LIGF batches are shown in Fig. S3. The *I*_D_/*I*_G_ values of the three batches were 0.53, 0.57, and 0.51, with a mean ± standard deviation of 0.54 ± 0.03, indicating that the LIGF quality is uniform and the fabrication process is reproducible. To preliminarily evaluate the potential of LIGF as an LDI substrate, its UV absorption at 343 nm (the wavelength of the MS ion source laser used in this study) was tested. By measuring the transmittance (*T*) and reflectance (*R*), the absorption (*A* = 1 − *T* − *R*) was calculated to be as high as 98.5% (Fig. 2(i)); two additional independent measurements yielded values of 97.7% and 97.9% (Fig. S4), with a mean ± standard deviation of 98.0% ± 0.4% for the three replicates, demonstrating excellent UV absorption capability and meeting the fundamental requirement for an LDI substrate.

### 2.2. LIGF-LDI-MS analysis

**Fig. 3:**
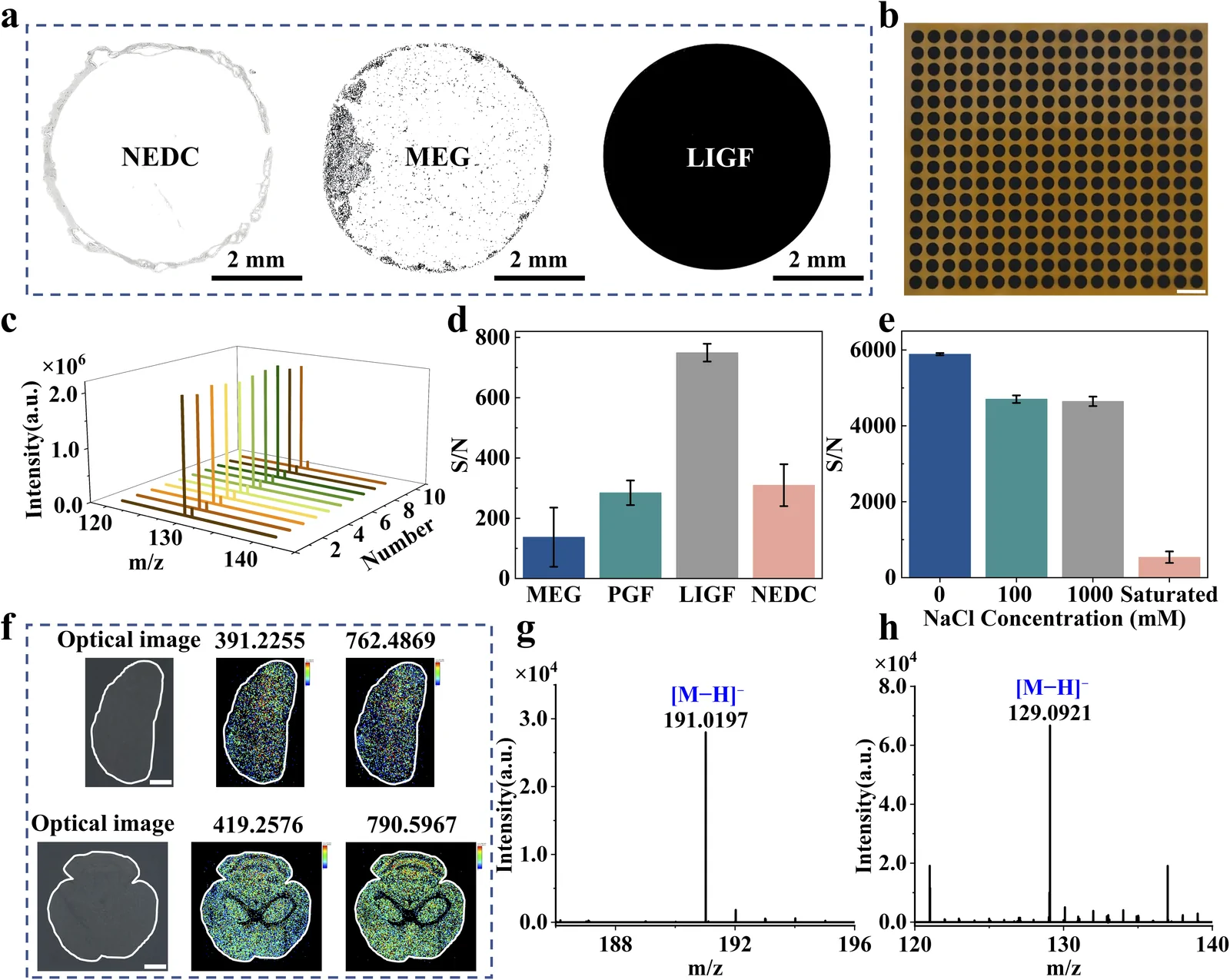
(a) Optical images (from left to right) of an NEDC matrix (mixed with 1 mM heptanoic acid), an MEG matrix (mixed with 1 mM heptanoic acid), and an LIGF substrate (with 1 mM heptanoic acid spotted on the surface). The volume for both matrix and analyte was 1 µL. (b) Optical image of an LIGF array. Scale bar: 7 mm. (c) LDI-MS spectra of heptanoic acid (1 nmol) acquired from 10 random positions on the LIGF surface. (d) S/N of citric acid (1 nmol) obtained from different matrices. Error bars represent standard deviation (*n* = 3). (e) S/N of 1 mM heptanoic acid in NaCl solutions at varying concentrations. Error bars represent standard deviation (*n* = 3). (f) LIGF-LDI-MSI images of mouse liver and brain tissue sections. Scale bars: 3 mm for liver, 2 mm for brain. Spatial resolution: 50 µm. (g) LIGF-LDI-MS spectrum of 1 nmol citric acid. (h) LIGF-LDI-MS spectrum of 1 mM heptanoic acid in a saturated NaCl solution.

To systematically evaluate the analytical performance of LIGF-LDI-MS, we first examined the background signal of the LIGF substrate. As shown in Fig. 2(j), LIGF exhibited minimal background interference in negative-ion mode, indicating excellent baseline cleanliness. In contrast, under identical experimental conditions, both mechanically exfoliated graphene (MEG) and laser-induced porous graphene film (PGF) showed prominent background peaks corresponding to carbon clusters (Fig. S5), highlighting the unique advantage of LIGF as a substrate in negative-ion mode. Furthermore, we validated the detection capability of LIGF-LDI-MS using heptanoic acid. Fig. 2(k) shows that a distinct [M–H]^−^ ion signal (*m*/*z* 129.0921) for heptanoic acid was obtained using LIGF-LDI-MS, with almost no background interference. Sensitivity tests (Fig. 2(l) and S6) revealed that the LOD (signal-to-noise ratio (S/N) > 3) for benzoic acid, linolenic acid, heptanoic acid, malic acid, and *trans*-aconitic acid reached 1 fmol, 30 pmol, 7 fmol, 10 pmol, and 1 pmol, respectively. For comparison, the sensitivity of the conventional matrix *N*-(1-naphthyl)ethylenediamine dihydrochloride (NEDC), as well as anti-reflective stainless steel surface and porous silicon substrate, was evaluated under identical conditions (Fig. S7–S9). For all tested compounds except heptanoic acid, LIGF-LDI-MS afforded higher S/N than NEDC-LDI-MS. Moreover, LIGF exhibited superior sensitivity compared with the anti-reflective surface and porous silicon substrate. Additionally, we investigated the uniformity of sample distribution on the substrate surface. Fig. 3(a), left and middle, show the pronounced “coffee-ring” effect formed after the drying of heptanoic acid solutions mixed with NEDC (7 mg mL^−1^) and MEG (1 mg mL^−1^), respectively. In contrast, droplet deposition of the same heptanoic acid solution on the LIGF surface resulted in a more uniform distribution (Fig. 3(a), right), which was corroborated by the corresponding signal profiles (Fig. 3(c), LDI-MS spectra of heptanoic acid acquired from ten random spots on the LIGF surface, with the relative standard deviation (RSD) of their signal intensities calculated to be approximately 2.26%). Fig. 3(b) displays a circular LIGF array with a diameter of 3 mm, demonstrating the capability of this method for high-throughput MS analysis. Benefiting from the high UV absorption of LIGF (∼98% absorptance, Fig. 2(i)), this substrate enables efficient desorption/ionization at relatively low laser energy. As shown in Fig. 3(d), LIGF achieved the highest S/N for 1 nmol citric acid at a laser pulse energy of 1.92 µJ per pulse compared with the other three matrices. Fig. 3(g) presents a representative LDI-MS spectrum of citric acid obtained under these conditions using the LIGF substrate, while results for other matrices are shown in Fig. S10.

High-salt environments represent a non-negligible interfering factor in LDI-MS analysis, as the presence of salts can significantly compromise the ionization efficiency and signal stability of analytes. Therefore, the salt tolerance of the substrate material is a critical parameter for evaluating its potential for practical applications in complex sample analysis. To evaluate the salt tolerance of the LIGF substrate, we systematically assessed its analytical performance in saline systems. When detecting 1 mM heptanoic acid in NaCl solutions of varying concentrations, the LIGF substrate maintained high S/N (Fig. 3(e)); the corresponding representative LDI-MS spectra are shown in Fig. 3(h) and S11. Notably, even in a saturated NaCl solution, an S/N of 511.1 was achieved for heptanoic acid (Fig. 3(h)), indicating the excellent salt tolerance of LIGF. Under the same condition, the conventional NEDC matrix yielded an S/N of 323.9 for heptanoic acid (Fig. S12). To further validate this advantage, pyruvic acid and linoleic acid (1 mM each) were analyzed in saturated NaCl solution. The results similarly showed that LIGF provided higher S/N than the NEDC matrix (Fig. S13), further confirming the superior analytical stability of LIGF in salt-containing solutions. Furthermore, to elucidate the unique advantages of the fibrous graphene structure in negative-ion mode, we systematically compared LIGF with MEG and PGF. A series of 1 mM standards—including citric acid, benzoic acid, malic acid, *trans*-aconitic acid, sugars, amino acids, and fatty acids—were analyzed under identical conditions (Fig. S14–S27). The results demonstrated that LIGF exhibited the lowest background interference and the highest signal intensity across all tested analytes, significantly outperforming both MEG and PGF. This highlights the distinct role of the fibrous structure in promoting the desorption/ionization process in negative-ion mode.

To demonstrate the applicability of LIGF-LDI-MS for biological sample analysis, we further analyzed MCF-7 and HT-1080 cell samples (Fig. S28), successfully detecting endogenous metabolites such as decanoic acid and palmitic acid. These collective results indicate the excellent capability of the LIGF substrate for detecting acidic metabolites in negative-ion mode. Furthermore, we applied LIGF to mass spectrometry imaging (MSI) of mouse brain and liver tissue sections (experimental workflow shown in Fig. S29). Spatial metabolic distribution information was acquired *via* 3D scanning mode (Fig. 3(f)) and linear scanning mode (Fig. S30). The corresponding list of tentatively identified metabolites is provided in Table S2, and representative LIGF-LDI-MS spectra are shown in Fig. S31. In summary, the LIGF substrate exhibits multiple advantages in negative-ion mode, including high salt tolerance, low background interference, and high sensitivity. Its fibrous structure confers superior desorption/ionization efficiency compared to other graphene-based materials, demonstrating promising potential for both cellular metabolite profiling and spatial metabolomics of tissue sections.

### 2.3. Field-enhancement effect at LIGF tips

**Fig. 4:**
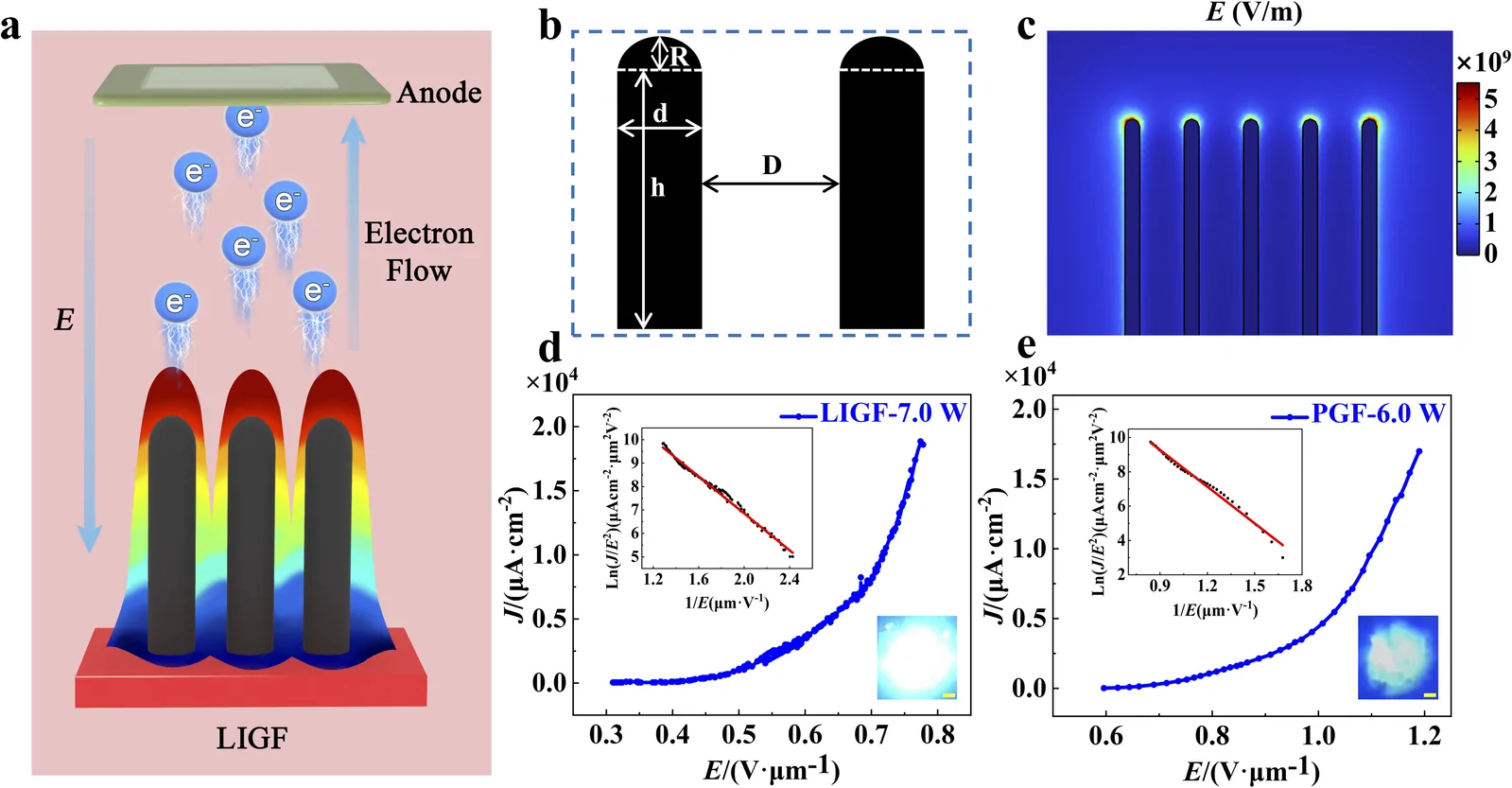
(a) Schematic of electron emission from the LIGF tip. (b) Model illustration of LIGF. (c) Simulation of the electric field enhancement at the LIGF tip. (d) and (e) Field emission characteristics (F–N plots) of the LIGF and PGF cold cathodes. Insets: optical images of the corresponding electron beam spots for LIGF (d) and PGF (e). Scale bars: 1 mm.

Building on the favorable detection performance of LIGF for acidic metabolites in negative-ion mode, we hypothesized that its superior desorption/ionization efficiency originates from a local field-enhancement effect induced by the unique fibrous morphology of the material. Under the negative high-voltage condition (−4000 V) applied in negative-ion mode, the LIGF tip is expected to generate a highly localized, strong electric field, which could significantly promote the deprotonation of analytes, particularly acidic compounds, thereby favoring the generation of stable [M–H]^−^ ions. To validate this hypothesis, we systematically investigated the field-enhancement effect at the LIGF tip through a combination of simulation and experimental measurement. The tip morphology of LIGF is shown in Fig. S32. Based on this, we established a model for simulation with dimensions illustrated in Fig. 4(b): *R* = 122.5 nm, *d* = 245 nm, *h* = 465 µm, and *D* = 735 nm. The simulation conditions were maintained consistent with the actual experimental environment: a voltage of −4000 V was applied to the LIGF, and the environment was kept consistent with the ion source (ambient temperature and atmospheric pressure). It should be noted that a representative geometry of a single LIGF was adopted for the simulation, which deviates from the actual conditions to some extent. Therefore, the simulation was employed only for qualitative assessment of the field enhancement effect at the LIGF tip. The simulation results (Fig. 4(c)) reveal a pronounced field enhancement at the LIGF tip. To further corroborate this finding, we conducted related experimental validation. A schematic of electron emission from the LIGF tip is shown in Fig. 4(a). The LIGF served as the cathode connected to a negative high-voltage supply, and an indium tin oxide (ITO) glass plate screen-printed with a phosphor layer (*i.e.*, the fluorescent screen, approximately 40 mm × 90 mm) served as the anode, with the anode being grounded. The distance between the LIGF cold cathode tip and the anode was about 5 mm. Electrons emitted from the cathode struck the fluorescent screen, producing a field emission pattern, which was recorded through a quartz window on the vacuum chamber using a camera (see insets in the lower right corners of Fig. 4(d) and (e)). By gradually increasing the voltage from 0 V, we recorded the field emission characteristics of LIGF and PGF (emission area ≈ 20 mm^2^).

The F–N plots for both materials essentially showed a linear relationship between ln(*J*/*E*^2^) and 1/*E*, expressed by the equation:1
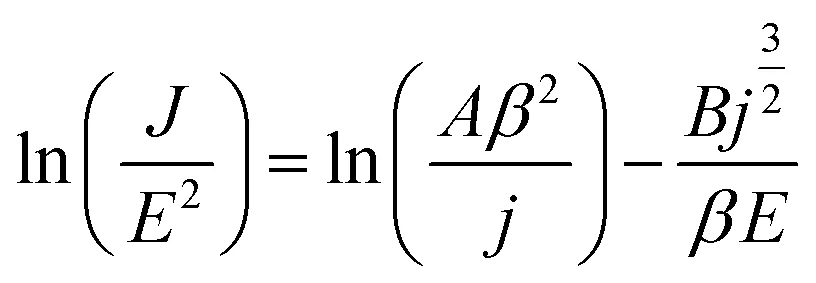
where *A* = 1.54 × 10^−6^ A eV V^−2^, *B* = 6.83 × 10^3^ eV^−3/2^ V µm^−1^, *φ* is the work function of the cathode material representing the energy barrier for electron emission into vacuum, and *β* is the field enhancement factor.^37^ Denoting the slope of the ln(*J*/*E*^2^) *versus* 1/*E* plot as *K*, we obtain from eqn (1):2
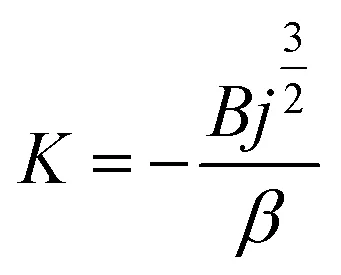
thus, the calculation formula for *β* is:3
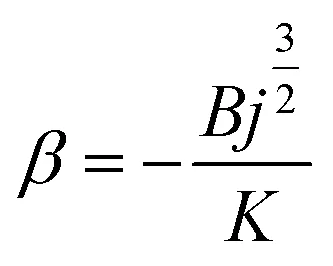


Taking *φ* = 5 eV,^37,38^ the calculated field enhancement factors *β* for LIGF and PGF are 19 381 and 10 695, respectively. The *β* value for LIGF is 1.8 times that of PGF, indicating a more significant field-enhancement effect at the graphene fiber tip. Furthermore, the insets in Fig. 4(d) and (e) show that the electron beam spot for LIGF is noticeably brighter, providing additional evidence for its stronger local electric field enhancement capability. Using six representative standard compounds (citric acid, malic acid, *trans*-aconitic acid, sucrose, maltose, and linolenic acid) as examples (Fig. S14, S16–S19, S23, and S24), the S/N obtained with the LIGF substrate were 4.7- to 57.1-fold higher than those obtained with PGF. This indicates that, in negative ion mode, the higher field enhancement factor at the LIGF tip facilitates the deprotonation of acidic metabolites, thereby improving detection sensitivity and achieving superior analytical performance. To further confirm that the field enhancement effect at the LIGF tip plays a crucial role in negative ion mode, we also performed LDI-MS analysis of the nine standard compounds using the LIGF substrate in positive ion mode (Fig. S33). All standard compounds exhibited S/N values lower than those obtained in negative ion mode. This result demonstrates the limited detection efficiency of the LIGF substrate in positive ion mode and further corroborates the specificity of the tip-enhanced field effect in promoting ionization efficiency in negative ion mode.

Integrating simulation and experimental results, this study elucidates the physical underpinnings of the highly efficient desorption/ionization achieved by LIGF in negative-ion mode. Finite element simulation intuitively reveals a pronounced localization and enhancement of the electric field at the LIGF tip. Further field emission experiments demonstrate that the field enhancement factor for LIGF is significantly higher than that for PGF. These results collectively demonstrate that the fibrous tip architecture of LIGF can effectively focus and amplify the local electric field. In negative-ion mode, this effect, by intensifying the surface electric field, markedly accelerates the deprotonation process of analytes, especially acidic molecules, thereby yielding stronger [M–H]^−^ signals. These findings collectively offer a mechanistic explanation for the superior performance of LIGF-LDI-MS.

### 2.4. Analysis of clinical serum samples

The persistent limitations in PC diagnosis stem from its clinically silent nature and the absence of highly specific symptoms, which collectively hinder timely detection and curative intervention.^2,3^ Current diagnostic strategies primarily rely on imaging examinations (such as contrast-enhanced CT and MRI) and detection of the serum tumor marker CA19-9. However, the former has limited resolution for smaller lesions and struggles to reliably differentiate malignant tumors from inflammatory pseudotumors; the latter is frequently elevated in benign hepatobiliary and pancreatic diseases, rendering its specificity and positive predictive value suboptimal.^4,5^ Furthermore, tissue biopsy, considered the diagnostic gold standard, is an invasive procedure associated with certain risks of complications. Therefore, developing a novel detection technology with high sensitivity, high specificity, and a non-invasive nature holds practical clinical value for improving the diagnostic efficacy for PC.

**Fig. 5:**
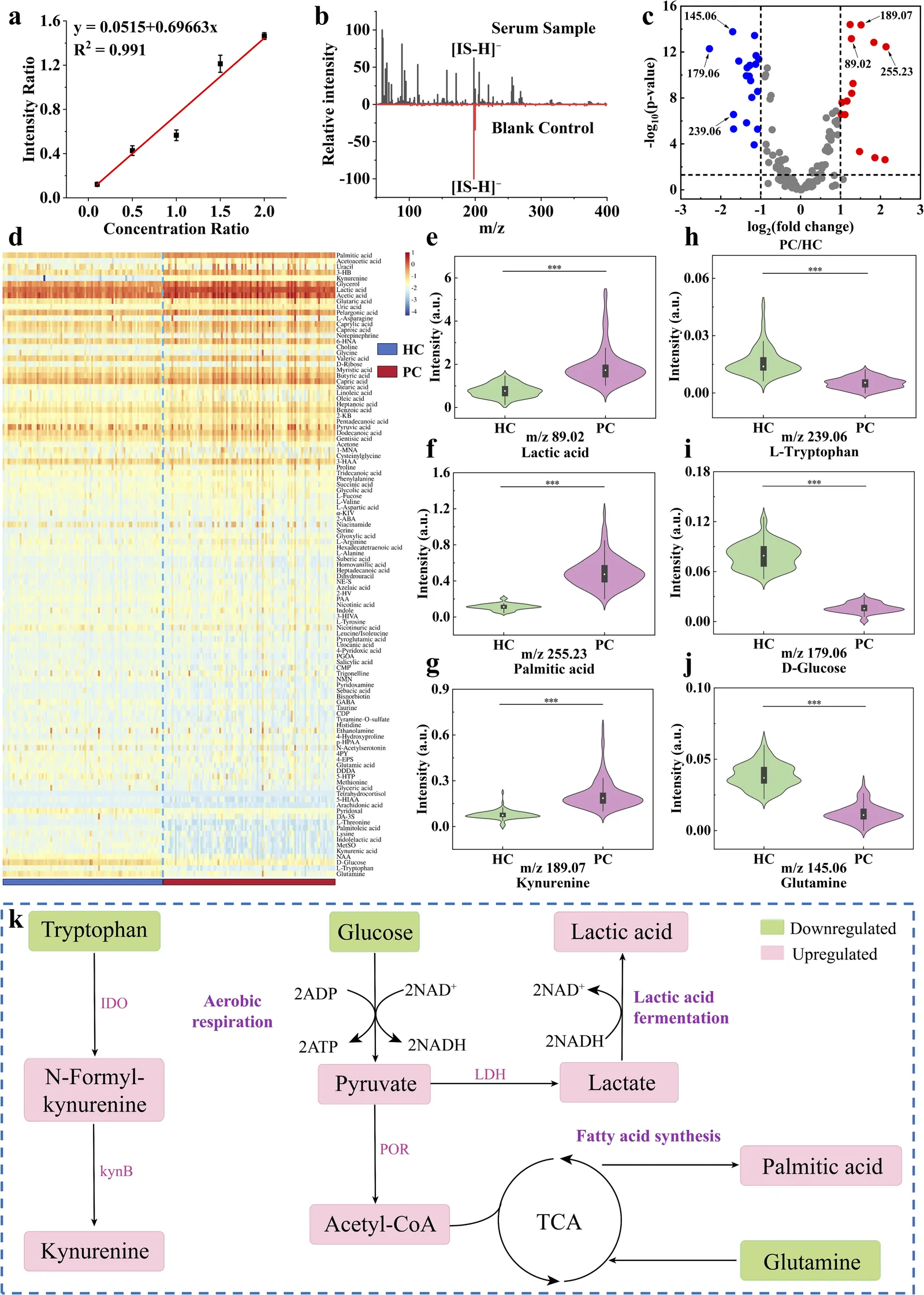
(a) Calibration curve of the l-phenylalanine/IS signal intensity ratio. (b) Comparison of LIGF-LDI-MS spectra from a serum sample and a blank control. (c) Volcano plot comparing PC patients and HC (|log_2_ FC| > 1, *p* < 0.05). (d) Heatmap of normalized MS features from HC and PC patients. (e)–(j) Violin plots showing the abundance of six representative differential metabolites in HC *vs.* PC patients (***p < 0.001). (k) Metabolic pathways of the six representative differential metabolites.

Using the LIGF array enables rapid high-throughput MS analysis. Each LIGF spot with a diameter of 3 mm can be fabricated within 12 s. For serum analysis, only 1 µL of the pretreated serum sample needs to be deposited onto the LIGF surface without the need for matrix mixing. A PI film with the size of a glass slide (76 mm × 25 mm) can yield 94 LIGF spots for serum analysis, with a material cost of only 0.049 USD per slide. In this study, LIGF-LDI-MS technology was employed to differentiate serum samples from PC patients and healthy controls (HC) through metabolic fingerprinting analysis, with the detailed experimental workflow illustrated in Fig. S34. To facilitate quantitative comparison, the laser energy was fixed at 10.00 µJ per pulse. For signal normalization, the exogenous compound L-3-chlorophenylalanine was systematically introduced into all serum samples as an internal standard (IS). This normalization protocol effectively corrected for instrumental fluctuations by performing ratio-based quantification of endogenous metabolite signals relative to the IS signal intensity. Method validation using the representative endogenous metabolite l-phenylalanine demonstrated a good linear proportionality (*R*^2^ = 0.991) between the analyte/IS concentration ratio and the corresponding signal intensity ratio across a serial dilution series (Fig. S35 and 5(a)), confirming the reliability of our normalization approach. Representative MS spectra from quality control (QC) serum samples and blank controls (containing only IS) are presented in Fig. 5(b). Based on exact mass measurements (error < 5 ppm) of QC samples and matching against the HMDB database, 109 metabolites were tentatively identified, predominantly including amino acids and their derivatives, organic acids, carbohydrates, fatty acids and lipids, nucleotide metabolites, vitamins and cofactors, neurotransmitters and related metabolites, among other classes (detailed in Table S5). Identification was performed by accurate mass matching, which is considered as putative identification at confidence level 3. It should be noted that structural confirmation of isomeric compounds requires further orthogonal analysis.

The experimental cohort comprised 195 serum samples (101 PC patients and 94 age-matched controls). Cluster analysis of the normalized metabolic profiles revealed a clear separation between PC patients and HC, as visualized in the heatmap (Fig. 5(d)). All metabolites were sorted in descending order according to their fold change in signal intensity between PC and HC (PC/HC). Specifically, metabolites with greater upregulation in the PC group relative to the HC group were positioned at one end of the heatmap, while those with greater downregulation were positioned at the opposite end. This differential pattern suggests the presence of disease-specific metabolic remodeling, warranting subsequent biomarker validation studies. Further analysis *via* volcano plot visualization, applying a screening threshold of |log_2_ FC| > 1 and *p* < 0.05, revealed 28 metabolites exhibiting marked metabolic alterations between PC patients and HC (Fig. 5(c)). The differential metabolites identified in this study collectively point to the core features of metabolic reprogramming in PC. The decreased serum glucose level coupled with elevated lactate aligns with the Warburg effect, characteristic of aberrant glycolytic activation in tumor cells.^39,40^ Concurrent tryptophan depletion and kynurenine accumulation indicate activation of the immunosuppressive tryptophan metabolism pathway mediated by indoleamine 2,3-dioxygenase 1 (IDO1) and tryptophan 2,3-dioxygenase (TDO) within the tumor microenvironment.^41–45^ Furthermore, glutamine consumption may provide biosynthetic precursors for cancer cells,^46–48^ and its association with the observed increase in palmitic acid levels reflects a restructuring of lipid metabolism.^49–51^ Notably, the upregulated metabolites identified above are closely associated with the biological characteristics of pancreatic cancer. Activation of the tryptophan–kynurenine pathway has been demonstrated to promote immune evasion in pancreatic cancer, and elevated IDO1 expression in pancreatic cancer tissues is significantly correlated with poor patient prognosis.^43,52,53^ As an endogenous ligand of the aryl hydrocarbon receptor (AhR), kynurenine induces the differentiation of regulatory T cells (Tregs) while suppressing effector T cell function,^44,54,55^ thereby establishing an immunosuppressive microenvironment in pancreatic cancer. Furthermore, elevated levels of saturated fatty acids such as palmitic acid reflect enhanced *de novo* fatty acid synthesis in pancreatic cancer cells,^56,57^ a process that not only provides structural lipids for membrane biosynthesis but also influences cancer cell survival through the regulation of ER stress and lipotoxicity.^58,59^ Meanwhile, glutamine serves as a dependency source of non-essential amino acids for pancreatic cancer cells, providing carbon skeletons and nitrogen sources to support the synthesis of nucleotides and non-essential amino acids; its increased consumption reflects a pronounced reliance of pancreatic cancer cells on glutaminolysis.^46,48,60^ These upregulated metabolites are not randomly altered but collectively point to a systemic adaptation of pancreatic cancer in terms of nutrient acquisition, immune evasion, and lipid remodeling. These metabolic alterations are not isolated events but systematically represent an overall metabolic phenotype wherein PC cells competitively uptake nutrient substrates, alter energy metabolic pathways, and foster an immunosuppressive microenvironment. Violin plots for the six aforementioned representative differential metabolites are shown in Fig. 5(e)–(g) (upregulated in PC patients relative to HC) and Fig. 5(h)–(j) (downregulated in PC patients relative to HC). The related metabolic pathways are depicted in Fig. 5(k). For the identification of the six key metabolites, MS/MS validation was performed, as shown in Fig. S36. By comparing the MS/MS spectra obtained from authentic standards with those of the corresponding metabolites in serum samples, the structural assignment of these key metabolites was further confirmed, elevating the confidence level of identification to level 2.

**Fig. 6:**
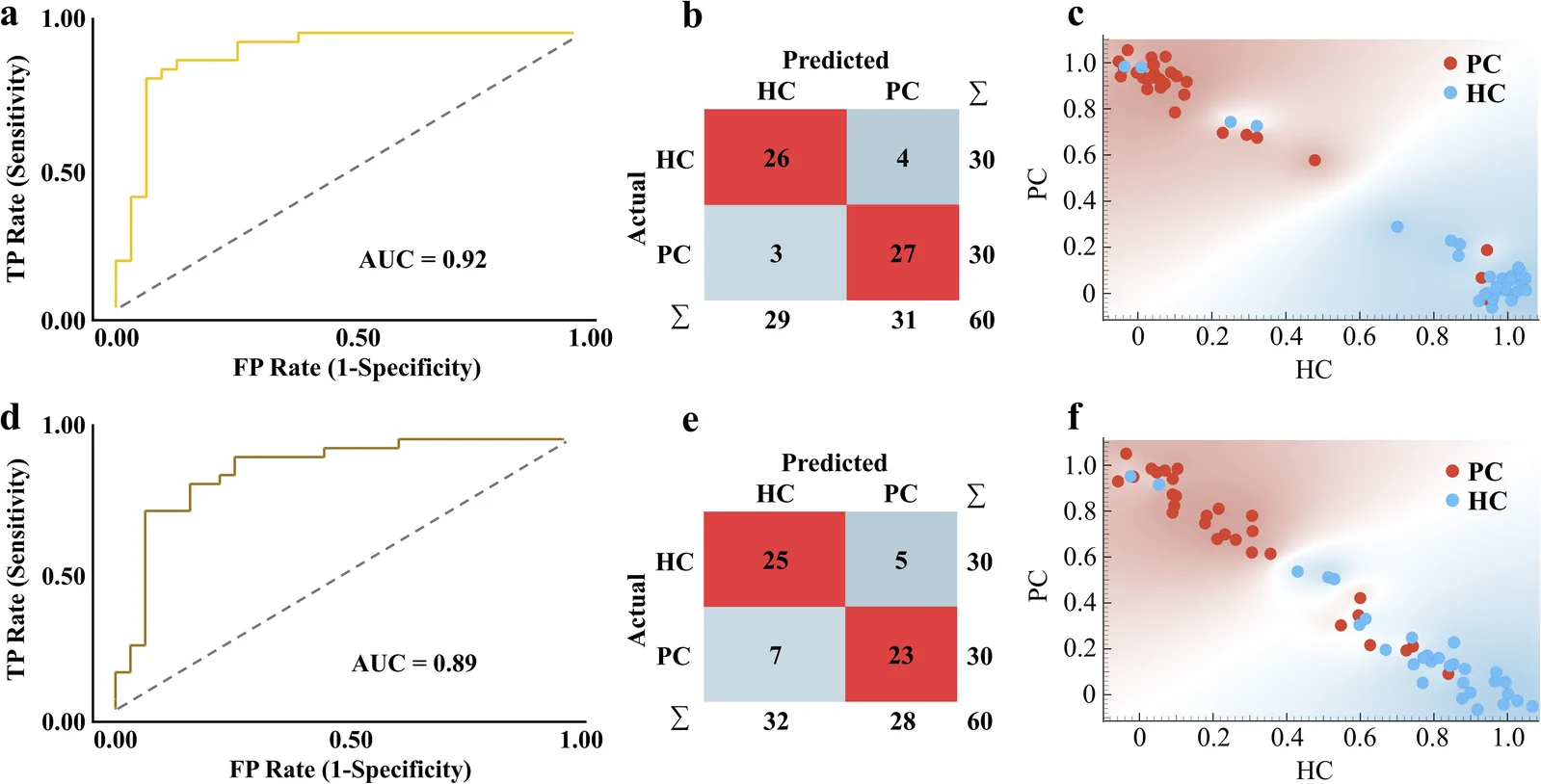
(a) ROC curve from the test set. (b) Confusion matrix showing 88% accuracy in blind validation. (c) Cluster visualization of sample separation. (d)–(f) Performance of a streamlined model using six selected metabolites, achieving 80% accuracy. The training set comprised 71 PC patients and 64 HC, while the test set consisted of 30 PC patients and 30 HC. The test set was used as the blind validation cohort to evaluate the predictive performance of all models.

Subsequent machine learning analysis incorporated eight distinct algorithms (Neural Network, Logistic Regression, Naive Bayes, Random Forest, SVM, kNN, Gradient Boosting, and AdaBoost) to process the metabolomic profiles encompassing all 109 detected metabolites from both the disease and control groups. Model performance was systematically evaluated using area under the curve (AUC) values and classification accuracy (CA), as detailed in Fig. S37 and Table S3. Notably, the neural network architecture demonstrated superior predictive capability relative to the other models and was therefore selected for the subsequent analytical phase. The dataset was partitioned into training (70%) and testing (30%) subsets *via* stratified randomization. The test set yielded a robust receiver operating characteristic (ROC) curve (Fig. 6(a)), while blind validation on 30 PC samples and 30 control samples revealed a diagnostic accuracy of 88% through confusion matrix analysis (Fig. 6(b)). The cluster visualization in Fig. 6(c) further corroborates the model's effective clustering ability. It is noteworthy that when feature selection was performed by retaining six metabolites exhibiting significant inter-group differences (*m*/*z* 89.0244, 145.0619, 179.0563, 189.0672, 239.0598, and 255.2328) while excluding other features, the simplified model maintained good diagnostic performance (accuracy of 80%), as quantitatively demonstrated by the replicated analytical workflow in Fig. S38, Table S4, and Fig. 6(d)–(f). It should be noted that Fig. 6(c) and (f) display the scatter distribution of the HC and PC probabilities predicted by the neural network model for each sample in the test set. In a binary classification model, the predicted probabilities of HC and PC for each sample satisfy the normalization constraint *P*(HC) + *P*(PC) = 1, giving rise to an inherent complementary relationship between the two probabilities. Consequently, in the two-dimensional space defined by the HC probability as the *x*-axis and the PC probability as the *y*-axis, the data points naturally distribute along the diagonal from the upper-left to the lower-right. Specifically, HC samples are concentrated in the lower-right region (high HC probability, low PC probability), whereas PC samples are concentrated in the upper-left region (high PC probability, low HC probability), with a clear separation between the two groups. This distribution pattern reflects the favorable classification performance of the model, rather than indicating the presence of multicollinearity in the dataset.

In addition, we performed Bootstrap resampling analysis (10 000 iterations) on the neural network model to calculate the 95% confidence intervals for each metric. The results are presented below. For the model based on all metabolites, the accuracy was 0.883 (95% CI: 0.800–0.950), with a sensitivity of 0.900 (95% CI: 0.800–1.000), a specificity of 0.867 (95% CI: 0.733–0.967), and an AUC of 0.924 (95% CI: 0.840–0.986). For the simplified model based on the six key metabolites, the corresponding values were 0.800 (95% CI: 0.700–0.900) for accuracy, 0.767 (95% CI: 0.600–0.900) for sensitivity, 0.833 (95% CI: 0.700–0.967) for specificity, and 0.885 (95% CI: 0.787–0.964) for AUC.

## 3 Conclusions

In summary, this study developed a high-performance LDI-MS method based on LIGF and applied it to serum metabolomics analysis of PC. Systematic material characterization confirmed that LIGF possesses a typical graphene fiber structure, high carbon content (∼95.28%), and excellent UV absorption capability (∼98%), establishing a solid physical foundation for its role as an efficient LDI substrate. Analytical performance evaluation demonstrated that LIGF in negative-ion mode exhibits multiple advantages, including extremely low background interference, high sensitivity (fmol-level), uniform signal distribution (with an RSD of approximately 2.26%), and high salt tolerance (achieving an S/N of 511.1 for 1 nmol heptanoic acid in saturated NaCl solution), which are clearly superior to those of the conventional organic matrix and other graphene-based materials. Mechanistic studies revealed that the pronounced local field-enhancement effect at the fibrous LIGF tip (field enhancement factor *β* ≈ 19 381) is the key physical reason for its efficient promotion of acidic metabolite deprotonation and the generation of strong [M–H]^−^ signals in negative-ion mode. The LIGF array enables rapid and high-throughput MS analysis. Each LIGF spot with a diameter of 3 mm can be fabricated within 12 s, and the prepared spots can be directly used for serum analysis without matrix mixing. A PI film with the size of a glass slide (76 mm × 25 mm) can yield 94 LIGF spots, with a material cost of only 0.049 USD, highlighting the unique advantages of extremely low cost, high efficiency, and time savings. Leveraging these advantages, we applied LIGF-LDI-MS to clinical serum metabolic fingerprinting of PC. A total of 109 metabolites were tentatively identified from 195 samples, and 28 differential metabolites exhibited significant alterations in PC. Their alteration patterns—such as enhanced glycolysis, activation of the tryptophan–kynurenine pathway, and remodeling of lipid metabolism—systematically reveal the characteristic metabolic reprogramming of PC. By mining the metabolic profiles with machine learning models, a neural network algorithm achieved a diagnostic accuracy of 88% in an independent test set. The LIGF is fabricated *via* a simple and rapid process, requires no additional chemical modification to serve directly as an LDI substrate, and offers notable advantages of low cost and short preparation time. This work not only demonstrates the capability of LIGF-LDI-MS as a rapid, low-cost, and high-throughput platform for metabolomic analysis—providing a validated foundation for its use in this field—but also offers a novel, non-invasive diagnostic strategy with high clinical value for PC.

## 4 Experimental section

### 4.1. Chemicals, reagents, and materials

*N*-(1-Naphthyl) ethylenediamine dihydrochloride (NEDC) was purchased from J&K Scientific Ltd (Beijing, China). Citric acid, benzoic acid, malic acid, *trans*-aconitic acid, linolenic acid, linoleic acid, oleic acid, heptanoic acid, carbohydrates, amino acids, HPLC grade ethanol and HPLC grade methanol were purchased from Shanghai Macklin Biochemical Technology Co., Ltd (Shanghai, China). Sodium chloride was purchased from Shandong Keyuan Bio-Chemical Co., Ltd (Shandong, China). Few-layered graphene nanosheets (3–6 layers) were purchased from Shenzhen Jiasheng New Materials Co., Ltd (Shenzhen, China) and prepared by mechanical exfoliation. The PI films were purchased from Shenzhen Beilong Electronics Co., Ltd (Shenzhen, China). The PI films had a thickness of 125 µm each.

### 4.2. Experimental procedure

In this work, a 125 µm-thick PI film was exposed to irradiation from a CO_2_ laser system (wavelength: 10.6 µm; Model: 4060, Ketai Laser, China) to generate LIGF. Prior to laser treatment, the PI substrate was thoroughly cleaned in sequential steps with ethanol and deionized water and then air-dried. The laser beam was raster-scanned across the PI surface to convert it into LIGF. Key processing parameters were set as follows: a lens-to-substrate focal distance of 5.0 cm, a scan speed of 300 mm s^−1^, and an output power of 7.0 W. The total exposure time was adjusted according to the intended sample area. For subsequent use as LDI-MS substrates, circular LIGF spots with a diameter of 3.0 mm were fabricated. Through computer software control, graphene fiber arrays can be fabricated simply and rapidly, making them applicable to high-throughput MS analysis and holding substantial potential for large-scale clinical application. Prior to MS analysis, analyte solutions were spotted onto the LIGF substrates and allowed to dry at room temperature.

For LDI-MSI applications, the preparation of LIGF substrates involved a specific two-step laser scanning procedure. The substrate was first scanned at a laser power of 7.0 W, followed by a second scan at 6.0 W, both performed at a constant speed of 300 mm s^−1^ and in orthogonal directions. This sequential scanning protocol aimed at removing loose carbonaceous debris and enhancing the surface uniformity of the LIGF, which is crucial for achieving uniform and consistent contact with tissue sections. For LDI-MSI analysis of tissue sections, square LIGF substrates with a side length of 15 mm were fabricated. Tissue sections were first mounted onto the LIGF substrates. Prior to LDI-MSI analysis, the system was evacuated using a diaphragm vacuum pump at a pressure of 0.035 MPa for approximately 30 min. This matrix-free approach successfully enabled MSI of the tissue sections. Furthermore, the computer-controlled laser patterning system allows for the fabrication of LIGF substrates with diverse dimensions and custom geometries, indicating that the utility of LIGF extends beyond the specific sizes and shapes utilized in the present work. The fabrication procedure and parameters for PGF are detailed in our previous work.^61^ The porous silicon substrate used in this study was prepared by etching a p-type silicon wafer in a mixed solution of nitric acid, hydrofluoric acid, and deionized water at a volume ratio of 1 : 1 : 2 for 1 h. The anti-reflective substrate modified with polydopamine was fabricated as follows: a 304 stainless steel substrate was mechanically polished, followed by one-step femtosecond laser processing to generate micro/nano-structured surface. Subsequently, the substrate was vertically immersed in a dopamine solution with magnetic stirring for 15 h. After the reaction, the substrate was rinsed with deionized water and dried in an oven to obtain the polydopamine-modified anti-reflective substrate. Detailed procedures can be found in previous literature.^62^

### 4.3. Collection and processing of serum samples

A total of 195 serum specimens were collected *via* venipuncture at The Second Affiliated Hospital of Jiangxi Medical College and stored at −80 °C. Of these, 101 were obtained from patients clinically diagnosed with PC, and the remaining 94 were from healthy control participants. The age range of the healthy control group was 28–81 years, with the majority of individuals aged 50–80 years. The age range of the PC group was 46–83 years. The healthy control and PC groups were closely matched in age, and both groups exhibited an approximately 1 : 1 male-to-female ratio with no statistically significant differences, thereby minimizing potential confounding by age and gender in the serum metabolomics study.

Serum samples were processed as follows: each serum sample was mixed with methanol/acetonitrile (1 : 1, v/v) at a volume ratio of 1 : 1. The mixture was ultrasonicated for 15 min, followed by vortexing for 5 min. Subsequently, the solution was centrifuged at 6000 rpm for 15 min, and 1 µL of the supernatant was deposited onto the LIGF surface. After air-drying, the sample was subjected to LDI-MS analysis. Mass spectral data for each serum sample were obtained by averaging spectra acquired from five random spots on the LIGF surface.

The selection of the internal standard for serum sample detection was based on the following considerations. In this study, l-3-chlorophenylalanine was chosen as the internal standard for the following reasons: (1) l-3-chlorophenylalanine is an exogenous compound that is not naturally present in serum samples, thus avoiding interference with the detection of endogenous metabolites; (2) its physicochemical properties, such as molecular weight, polarity, and ionization efficiency, are similar to those of the target metabolites in serum, enabling effective correction of signal variations caused by differences in sample spotting volume and fluctuations in instrument response; (3) in LDI-MS analysis, l-3-chlorophenylalanine generates strong characteristic mass spectrometric signals that do not overlap with those of endogenous metabolites, facilitating accurate quantification.

### 4.4. Characterizations

Scanning electron microscope (SEM) images were obtained using a ZEISS Sigma 360 (Carl Zeiss AG, Oberkochen, Germany). The element distributions on the LIGF surface were characterized by two-dimensional scanning using energy dispersive spectroscopy (EDS) (SU5000, Hitachi, Tokyo, Japan). Light reflectivity and transmittance curves were measured using a UV-VIS-NIR spectrophotometer (Lambda 1050+, PerkinElmer, Waltham, United States). X-ray photoelectron spectroscopy (XPS) spectra were obtained using an AXIS SUPRA+ (Shimadzu Corp., Tokyo, Japan). Raman spectra were obtained using a DXR laser Raman spectrometer (Thermo Fisher Scientific, Waltham, United States).

The ultra-high vacuum field emission testing system employed in this study utilized a diode-type field emission testing configuration. During testing, an ITO-coated glass substrate served as the anode, while laser-induced graphene on a PI substrate, secured onto a stainless steel plate using conductive adhesive tape, functioned as the cathode. To acquire electron beam spot images, a simple phosphor screen was fabricated by screen-printing a phosphor layer onto ITO glass. Electrons striking this phosphor layer generate light emission, allowing the study of electron distribution based on the shape and intensity profile of the luminescent spot. A high-voltage DC power supply was connected to provide a negative high voltage to the cathode, with the anode being grounded. All tested samples were circular with a diameter of 5 mm, and the anode–cathode gap was set at 5 mm.

### 4.5. Animals, tissue, and cell suspension preparation

BALB/cJGpt mice (5 weeks old, 15–18 g body weight) were acquired from GemPharmatech (Nanjing, China). Upon harvest, whole brain and liver tissues were rapidly frozen in liquid nitrogen and stored at −80 °C until further processing. Tissue sections were prepared using a Leica CM1950 cryostat (Leica Microsystems GmbH, Germany) at a chamber temperature of −20 °C and subsequently mounted onto the LIGF substrates. Before MSI analysis, the mounted sections were dried in a vacuum desiccator for about 30 min. All tissue sections used in this study had a thickness of 20 µm, and the MSI was performed with a spatial resolution of 50 µm.

MCF-7 and HT-1080 cells were purchased from BeNa Culture Collection. The cells were cultured in complete growth medium consisting of 90% Dulbecco's Modified Eagle Medium (DMEM), 10% fetal bovine serum (FBS), and 1% penicillin-streptomycin mixture, and maintained in a humidified incubator at 37 °C with 5% CO_2_. When cell confluency reached 85–90%, the medium was aspirated, and the cells were washed 2–3 times with phosphate-buffered saline (PBS), followed by digestion with 0.25% trypsin for 2–3 min. The resulting cell suspension was centrifuged at 900 rpm for 5 min. After discarding the supernatant, the cell pellet was resuspended in PBS for subsequent experiments.

### 4.6. LDI-MS analysis

MS analysis was performed using an atmospheric pressure scanning microprobe MALDI source coupled to an electrostatic field Orbitrap high-resolution mass spectrometer. The ion source incorporates a UV laser (Flare NX 343-0.2-2, wavelength: 343 nm) operating at a repetition rate of 2000 Hz. For each spectrum, signals from 50 consecutive laser shots were accumulated. The laser fluence was optimized by adjusting the deflection angle of an energy-attenuating lens to achieve an optimal balance between signal intensity and background noise. Ion images were reconstructed using MirionV3 software (TransMIT). For solution-phase analysis, 1 µL of sample was deposited onto the LIGF substrate and air-dried. For tissue imaging, sections mounted on LIGF were secured to a glass slide with ultra-thin double-sided tape and then loaded onto the LDI sample stage. All analyses, for both solution samples and tissue sections, were conducted in negative-ion mode.

### 4.7. Data analysis

Peaks were identified by comparing their precise masses with the Human Metabolome Database (HMDB) (https://www.hmdb.ca), ensuring a mass error of under 5 ppm. Additionally, previously published data (ref. 63–67) were also utilized to confirm the identifications.

The normalization workflow for serum sample data processing was as follows. First, the signal intensities of each endogenous metabolite feature peak in the serum sample were extracted. Then, the intensity of the characteristic peak of l-3-chlorophenylalanine in the same spectrum was used as a reference. Finally, the ratio of each endogenous metabolite signal intensity to that of l-3-chlorophenylalanine was calculated, and these relative intensity values were subsequently used for statistical analysis, heatmap construction, and machine learning. This normalization strategy effectively reduces the technical variations introduced by differences in sample spotting volume and fluctuations in instrument response, thereby ensuring reliable comparison of metabolite signal intensities across different samples.

### 4.8. Neural network model

(1) Network Architecture: the neural network classification model was implemented using the “Neural Network” module in Orange Data Mining (Version 3.39), which is built upon the “MLPClassifier” from scikit-learn. The model adopts a multilayer perceptron (MLP) architecture, consisting of:

Input layer: the number of neurons equals the number of input features;

Hidden layer: a single hidden layer containing 100 neurons;

Output layer: the number of neurons equals the number of classes (in this study, two classes: pancreatic cancer patients and healthy controls).

(2) The key hyperparameters of the model were configured as follows:

Activation function: ReLU was used in the hidden layer;

Optimizer: the Adam optimizer was employed for weight updates;

L2 regularization (*α*): set to 0.0001 to control model complexity and prevent overfitting;

Maximum number of iterations: set to 200;

Reproducibility: the “Replicable Training” option was enabled to fix the random seed, ensuring reproducibility of the training results.

All other parameters were kept at their default values in Orange.

(3) The model training and validation procedures were as follows:

Data splitting: the dataset was divided into a training set and an independent test set (the test set served as the blind validation set). The training set was used for model training and cross-validation, while the test set was completely withheld during the entire training process and used only for the final blind validation.

Parallel training and cross-validation of multiple models: in the “Test and Score” node, eight models—AdaBoost, Gradient Boosting, kNN, Logistic Regression, Naive Bayes, Neural Network, Random Forest, and SVM—were simultaneously trained and evaluated. This node internally performed 5-fold cross-validation for hyperparameter tuning and model selection, with AUC and classification accuracy (CA) as the primary evaluation metrics.

Blind validation on the independent test set: the test set, which had been completely isolated during training and cross-validation, was evaluated using the “Predictions” node to obtain the prediction performance (accuracy, confusion matrix, ROC curves, *etc.*) of each model on the independent test set, thereby objectively assessing the generalization ability of the models.

### 4.9. Material cost of the LIGF-LDI-MS technology

The fabrication time for each circular LIGF spot (3 mm in diameter) was approximately 12 s, allowing for rapid preparation prior to MS analysis. The PI film used in this study has dimensions of 210 mm × 297 mm and costs approximately 1.6 USD per sheet. Each sheet can yield around 3700 circular LIGF spots, meaning that a single film can meet the analysis demands of up to 3700 serum samples. This clearly demonstrates that the LIGF array-based LDI-MS method entails extremely low material costs and is well-suited for large-scale, high-throughput clinical sample analysis.

## Live subject statement

All experiments in this study were performed in compliance with relevant laws and the institutional guidelines of Ningxia University. The experiments were approved by the Science and Technology Ethics Committee of Ningxia University (Approval Nos. NXU-A-2025-289 and NXU-H-2025-290). This study involved human subjects, and informed consent was obtained from all human subjects.

## Author contributions

Zhihao Ye: writing – review and editing, writing – original draft, methodology, investigation, formal analysis, data curation, conceptualization. Haiyan Luo: formal analysis. Mengyang Song: formal analysis. Pei Ding: methodology. Chao Ding: resources. Junyu Chen: resources. Zongxiu Nie: writing – review and editing, validation, resources, project administration, conceptualization, funding acquisition. Yuze Li: writing – review and editing, validation, project administration, conceptualization, funding acquisition.

## Conflicts of interest

The authors declare that they have no known competing financial interests or personal relationships that could have appeared to influence the work reported in this paper.

## Supplementary Material

SC-OLF-D6SC04783A-s001

## Data Availability

The data that support the findings of this study are available from the corresponding author upon reasonable request. Supplementary information (SI): SEM characterization of LIG prepared at low laser powers; Raman and UV absorption data; sensitivity evaluation of LIGF substrate, NEDC matrix, and other representative substrates; salt tolerance assessment of LIGF substrate and NEDC matrix; test data for standards; MS/MS spectra; machine learning results; metabolite identification lists, *etc.* See DOI: https://doi.org/10.1039/d6sc04783a.
