# Supplementary material for: Field-enhanced graphene fiber arrays reveal serum metabolic alterations in pancreatic cancer
Source: Chem Sci. 2026 Aug 7;17(34):16170–85. doi: 10.1039/d6sc04783a (PMC13473998; doi:10.1039/d6sc04783a)
Supplement: Supplementary file 1 [file SC-017-D6SC04783A-s001.pdf]

Supplementary Information (SI) for Chemical Science.
This journal is © The Royal Society of Chemistry 2026

Supporting Information

Field-enhanced Graphene Fiber Arrays Reveal Serum Metabolic Alterations in Pancreatic Cancer

Zhihao Ye[1], Haiyan Luo[2], Mengyang Song[2], Pei Ding[3], Chao Ding[4], Junyu Chen[5,], Zongxiu Nie[2,6,7,*], and Yuze Li[2,*]*

[1]School of Physics, Ningxia University, Yinchuan 750021, China

[2]State Key Laboratory of High-Efficiency Utilization of Coal and Green Chemical Engineering, School of Chemistry and Chemical Engineering, Ningxia University, Yinchuan 750021, China

[3]Zhengzhou University of Aeronautics, Zhengzhou 450046, China

[4]People's Hospital of Ningxia Hui Autonomous Region, Yinchuan 750021, China

[5]Jiangxi Province Key Laboratory of Immunology and Inflammation, Jiangxi Provincial Clinical Research Center for Laboratory Medicine, Department of Clinical Laboratory, The Second Affiliated Hospital, Jiangxi Medical College, Nanchang University, Nanchang 330006, China

[6]State Key Laboratory of Tropic Ocean Engineering Materials and Materials Evaluation, School of Marine Science and Engineering, Hainan University, Haikou 570228, China

[7]Beijing National Laboratory for Molecular Sciences, Key Laboratory of Analytical Chemistry for Living Biosystems, Institute of Chemistry, Chinese Academy of Sciences, Beijing 100190, China

Correspondence: Junyu Chen (junyu94@outlook.com), Zongxiu Nie (znie@iccas.ac.cn), Yuze Li (liyuze@nxu.edu.cn)

Table of Content

	Contents	Page No.
**Figure S1**	Schematic of LIGF fabrication.	5
**Figure S2**	SEM images of graphene structures induced on PI film surface under laser powers of (a) 5.0 W and (b) 6.0 W.	6
**Figure S3**	Raman spectroscopic characterization of LIGF from two independent fabrication batches.	7
**Figure S4**	UV absorption characterization of LIGF from two independent fabrication batches.	8
**Figure S5**	LDI-MS spectra of (a) MEG and (b) PGF.	9
**Figure S6**	Limit of detection (S/N > 3) for linolenic acid, heptanoic acid, malic acid, and trans-aconitic acid by LIGF-LDI-MS.	10
**Figure S7**	Sensitivity assessment of NEDC-assisted LDI-MS for linolenic acid, heptanoic acid, malic acid, trans-aconitic acid, and benzoic acid.	11
**Figure S8**	Sensitivity assessment of anti-reflective surface-assisted LDI-MS for linolenic acid, heptanoic acid, malic acid, trans-aconitic acid, and benzoic acid.	12
**Figure S9**	Sensitivity assessment of porous silicon-assisted LDI-MS for linolenic acid, heptanoic acid, malic acid, trans-aconitic acid, and benzoic acid.	13
**Figure S10**	LDI-MS spectra of 1 nmol citric acid acquired at a laser energy of 1.92 μJ/pulse using (a) MEG, (b) PGF, and (c) NEDC as the substrate/matrix.	14
**Figure S11**	LIGF-LDI-MS spectra of 1 mM heptanoic acid in (a) 0 mM, (b) 100 mM, and (c) 1000 mM NaCl solutions.	15
**Figure S12**	NEDC-assisted LDI-MS spectrum of 1 mM heptanoic acid in a saturated NaCl solution.	16
**Figure S13**	(a), (b) LIGF-LDI-MS and (c), (d) NEDC-assisted LDI-MS spectra of 1 mM pyruvic acid and linoleic acid, respectively, in saturated NaCl solutions.	17
**Figure S14**	(a) MEG-LDI-MS, (b) PGF-LDI-MS, and (c) LIGF-LDI-MS spectra of 1 nmol citric acid.	18
**Figure S15**	(a) MEG-LDI-MS, (b) PGF-LDI-MS, and (c) LIGF-LDI-MS spectra of 1 nmol benzoic acid.	19
**Figure S16**	(a) MEG-LDI-MS, (b) PGF-LDI-MS, and (c) LIGF-LDI-MS spectra of 1 nmol malic acid.	20
**Figure S17**	(a) MEG-LDI-MS, (b) PGF-LDI-MS, and (c) LIGF-LDI-MS spectra of 1 nmol trans-aconitic acid.	21

**Figure S18**	(a) MEG-LDI-MS, (b) PGF-LDI-MS, and (c) LIGF-LDI-MS spectra of 1 nmol sucrose.	22
**Figure S19**	(a) MEG-LDI-MS, (b) PGF-LDI-MS, and (c) LIGF-LDI-MS spectra of 1 nmol maltose.	23
**Figure S20**	(a) MEG-LDI-MS, (b) PGF-LDI-MS, and (c) LIGF-LDI-MS spectra of 10 nmol L-isoleucine.	24
**Figure S21**	(a) MEG-LDI-MS, (b) PGF-LDI-MS, and (c) LIGF-LDI-MS spectra of 10 nmol L-phenylalanine.	25
**Figure S22**	MEG-LDI-MS spectra of 1 mg/mL linolenic acid (1 μL).	26
**Figure S23**	PGF-LDI-MS spectra of 1 mg/mL linolenic acid (1 μL).	27
**Figure S24**	LIGF-LDI-MS spectra of 1 mg/mL linolenic acid (1 μL).	28
**Figure S25**	(a) MEG-LDI-MS, (b) PGF-LDI-MS, and (c) LIGF-LDI-MS spectra of 1 mg/mL linoleic acid (1 μL).	29
**Figure S26**	(a) MEG-LDI-MS, (b) PGF-LDI-MS, and (c) LIGF-LDI-MS spectra of 1 mg/mL oleic acid (1 μL).	30
**Figure S27**	(a) MEG-LDI-MS, (b) PGF-LDI-MS, and (c) LIGF-LDI-MS spectra of 1 mg/mL heptanoic acid (1 μL).	31
**Figure S28**	LIGF-LDI-MS spectra of MCF-7 cells and HT-1080 cells.	32
**Figure S29**	Schematic diagram of the LIGF-LDI-MSI.	33
**Figure S30**	LIGF-LDI-MSI images of a mouse brain tissue section acquired in linear scanning mode.	34
**Figure S31**	Representative LIGF-LDI-MS spectra of (a) mouse liver tissue and (b) mouse brain tissue acquired in 3D mode. (c) Representative LIGF-LDI-MS spectra of mouse brain tissue acquired in linear scanning mode.	35
**Figure S32**	SEM image of the LIGF tip morphology.	36
**Figure S33**	LIGF-LDI-MS spectra of nine standard analytes in positive ion mode.	37
**Figure S34**	Experimental workflow for clinical serum sample analysis using LIGF-LDI-MS.	38
**Figure S35**	Overlaid LIGF-LDI-MS spectra of L-phenylalanine and the internal standard (IS) at different concentration ratios (0.1-2.0).	39
**Figure S36**	MS/MS spectra of six key metabolites (m/z: 89.02, 255.23, 189.07, 239.06, 179.06, and 145.06). (a–f) MS/MS spectra of six standards. (g–l) MS/MS spectra of six metabolites in serum.	40
**Figure S37**	Comparison between eight machine learning models.	41

**Figure S38**	Comparison between eight machine learning models using six selected metabolites.	42
**Table S1**	Elemental composition of the LIGF.	43
**Table S2**	List of metabolites identified in mouse brain and liver tissues.	44
**Table S3**	Comparison of classification performance of various machine learning models on data sets.	45
**Table S4**	Comparison of classification performance of various machine learning models on the dataset derived from the six representative differential metabolites.	46
**Table S5**	Tentatively Identified compounds from the clinical serum samples.	47–53

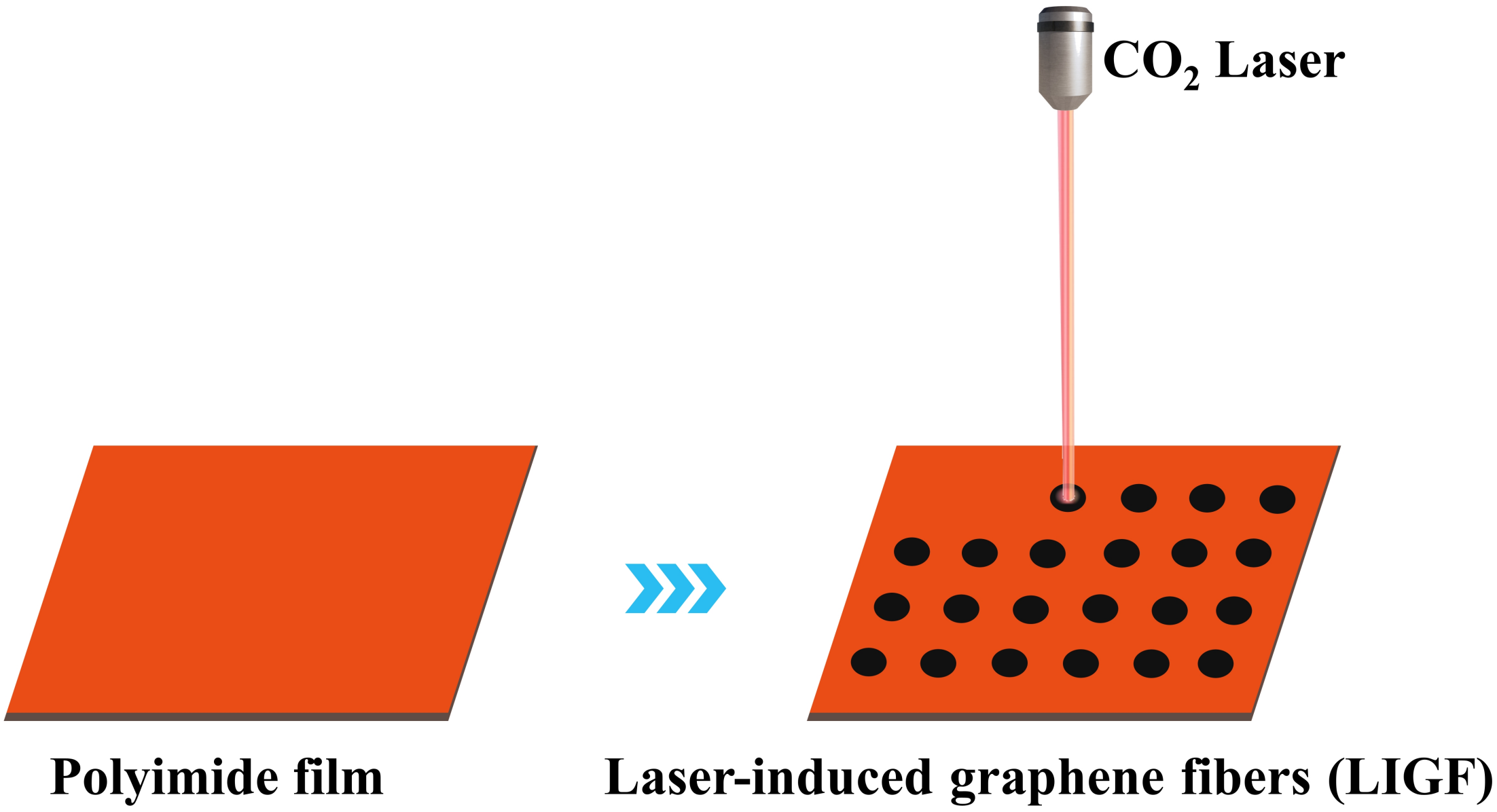

Figure S1. Schematic of LIGF fabrication.

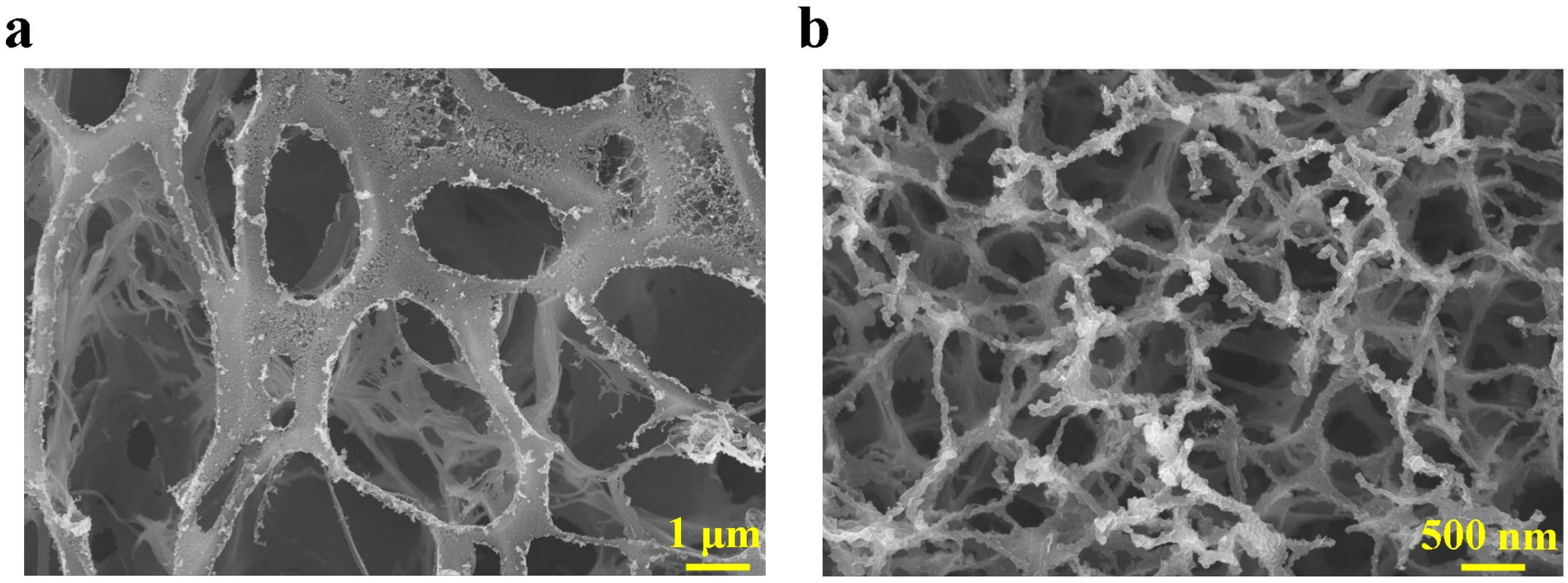

Figure S2. SEM images of graphene structures induced on PI film surface under laser powers of (a) 5.0 W and (b) 6.0 W.

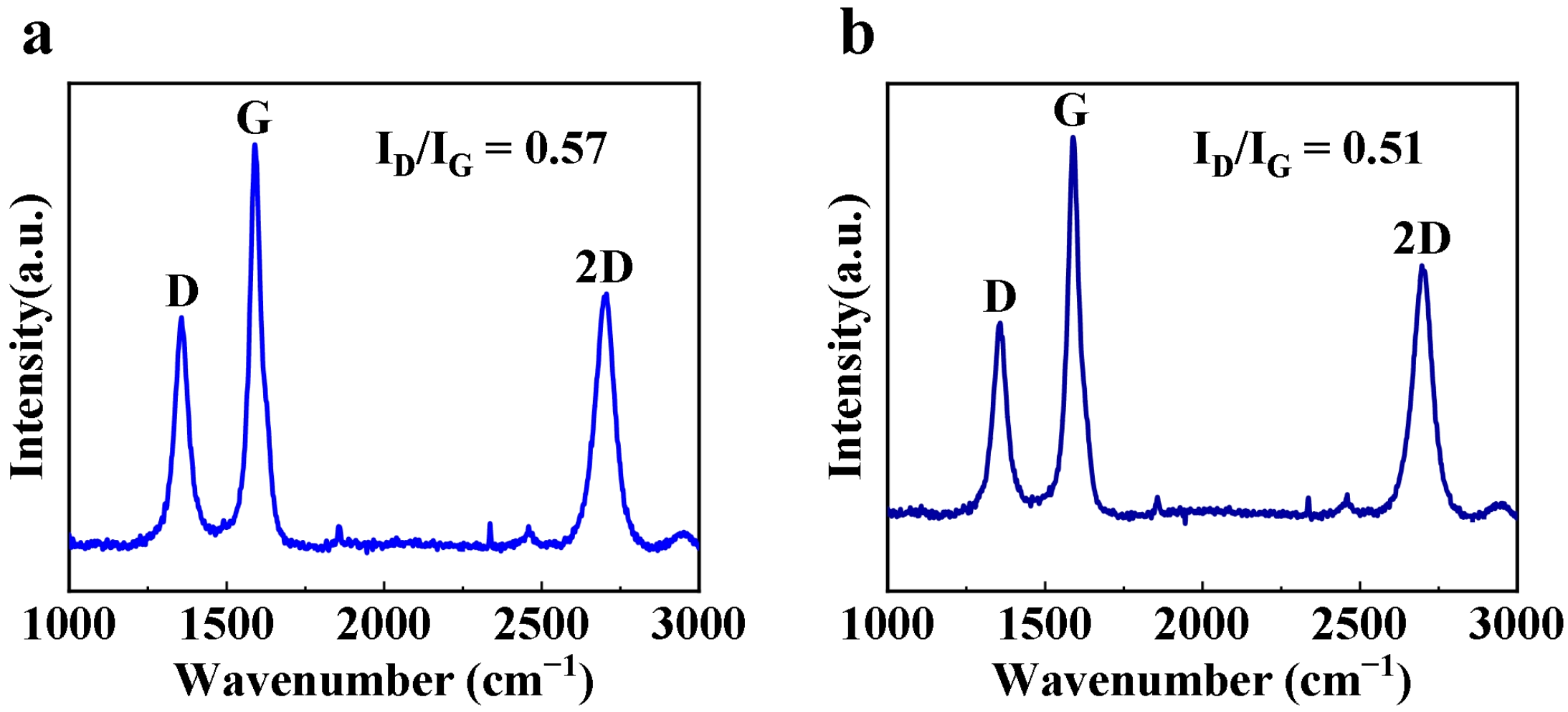

Figure S3. Raman spectroscopic characterization of LIGF from two independent fabrication batches.

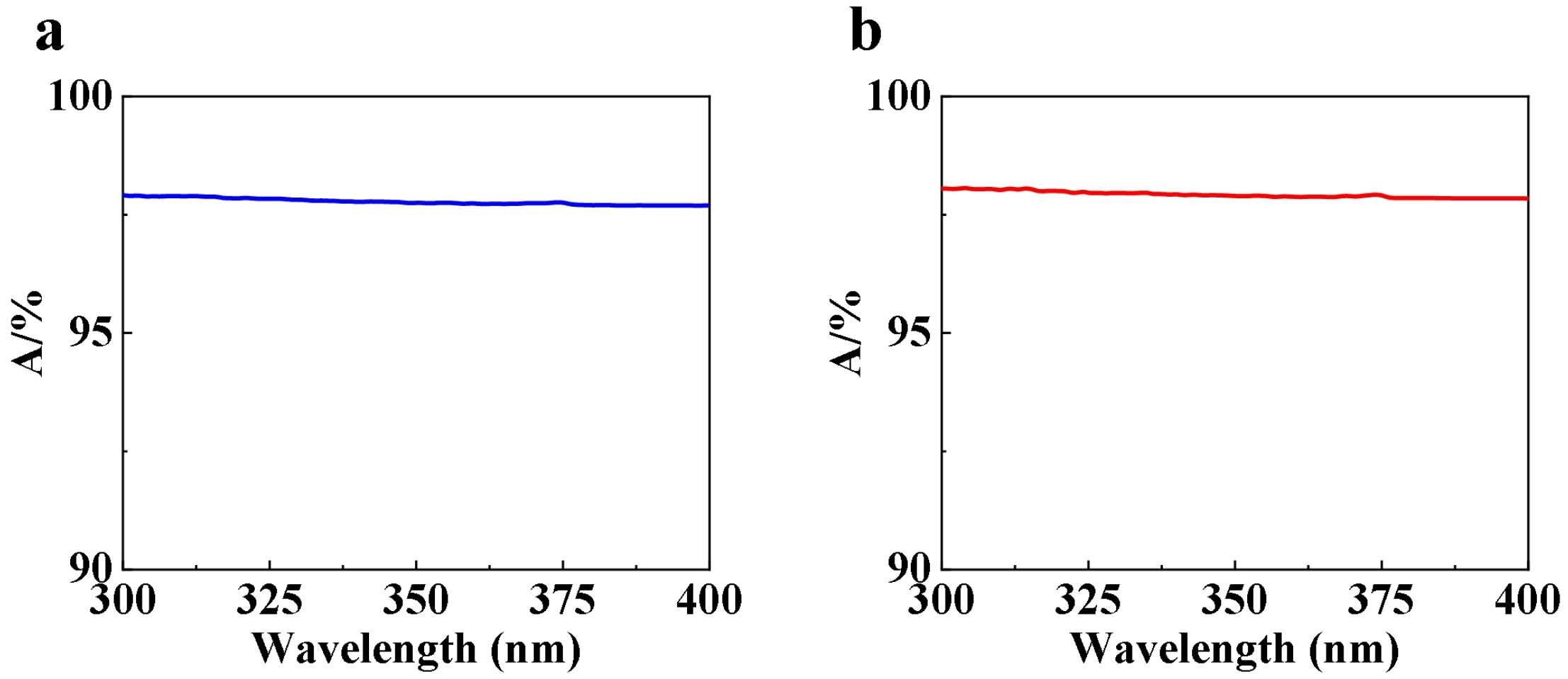

Figure S4. UV absorption characterization of LIGF from two independent fabrication batches.

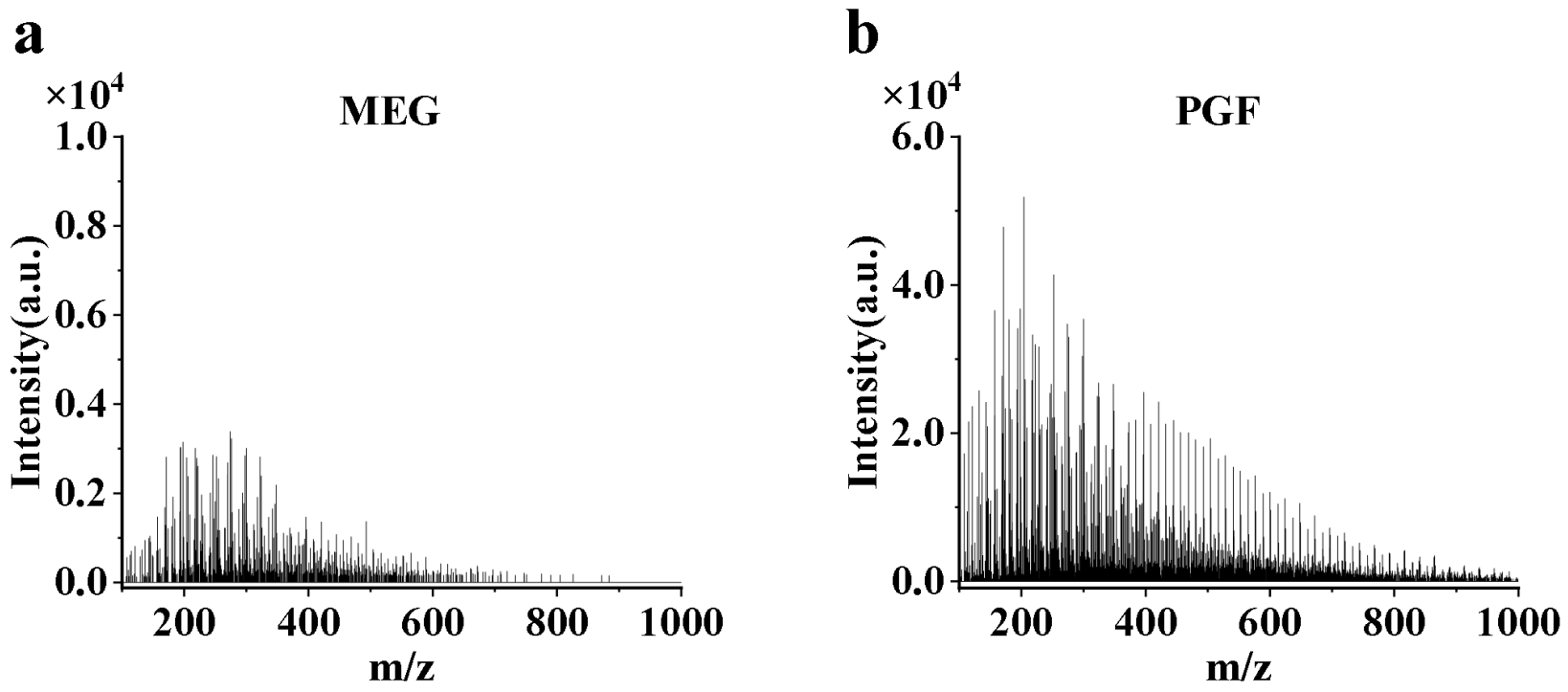

Figure S5. LDI-MS spectra of (a) MEG and (b) PGF.

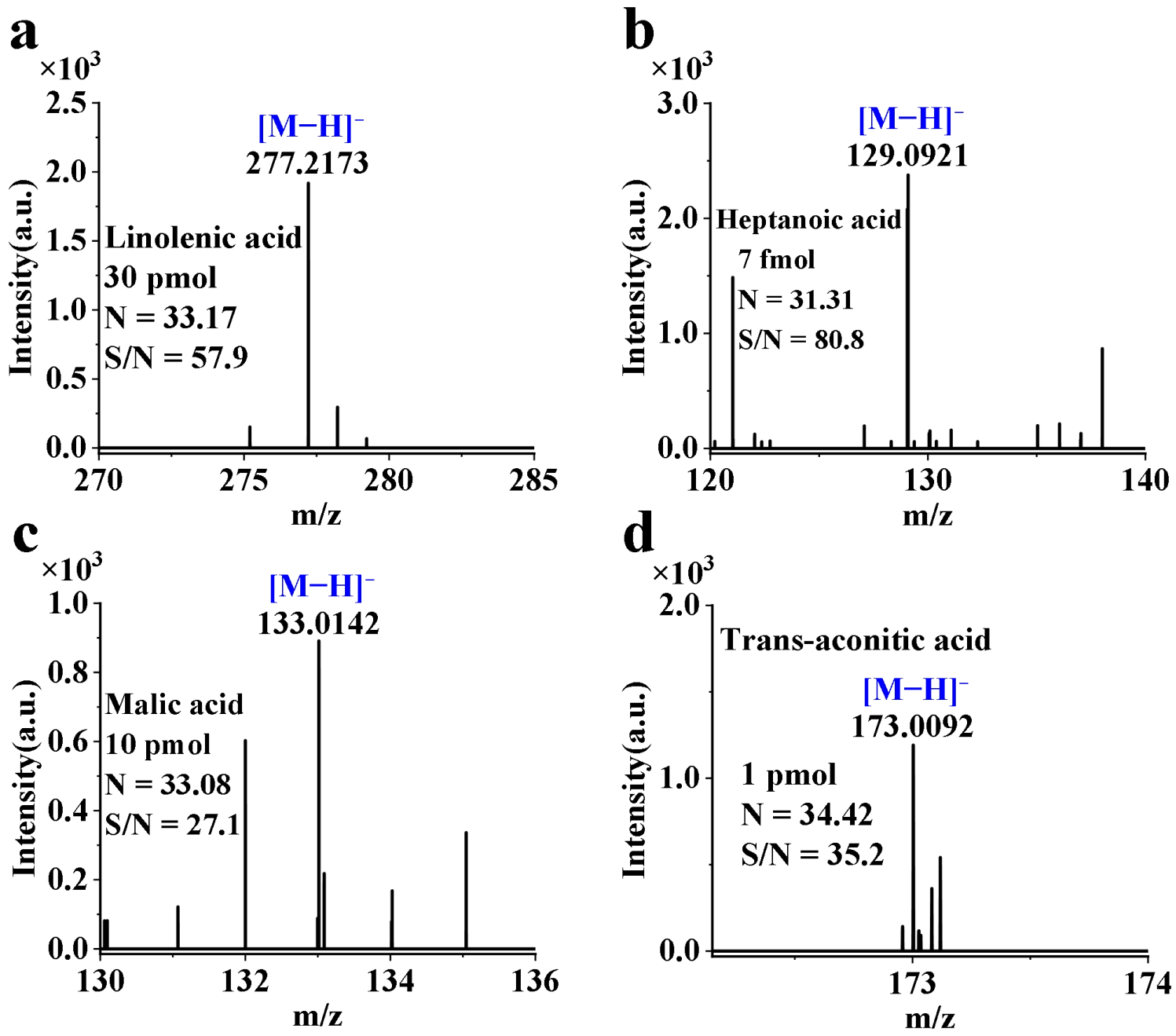

Figure S6. Limit of detection (S/N > 3) for linolenic acid, heptanoic acid, malic acid, and trans-aconitic acid by LIGF-LDI-MS: 30 pmol, 7 fmol, 10 pmol, and 1 pmol, respectively.

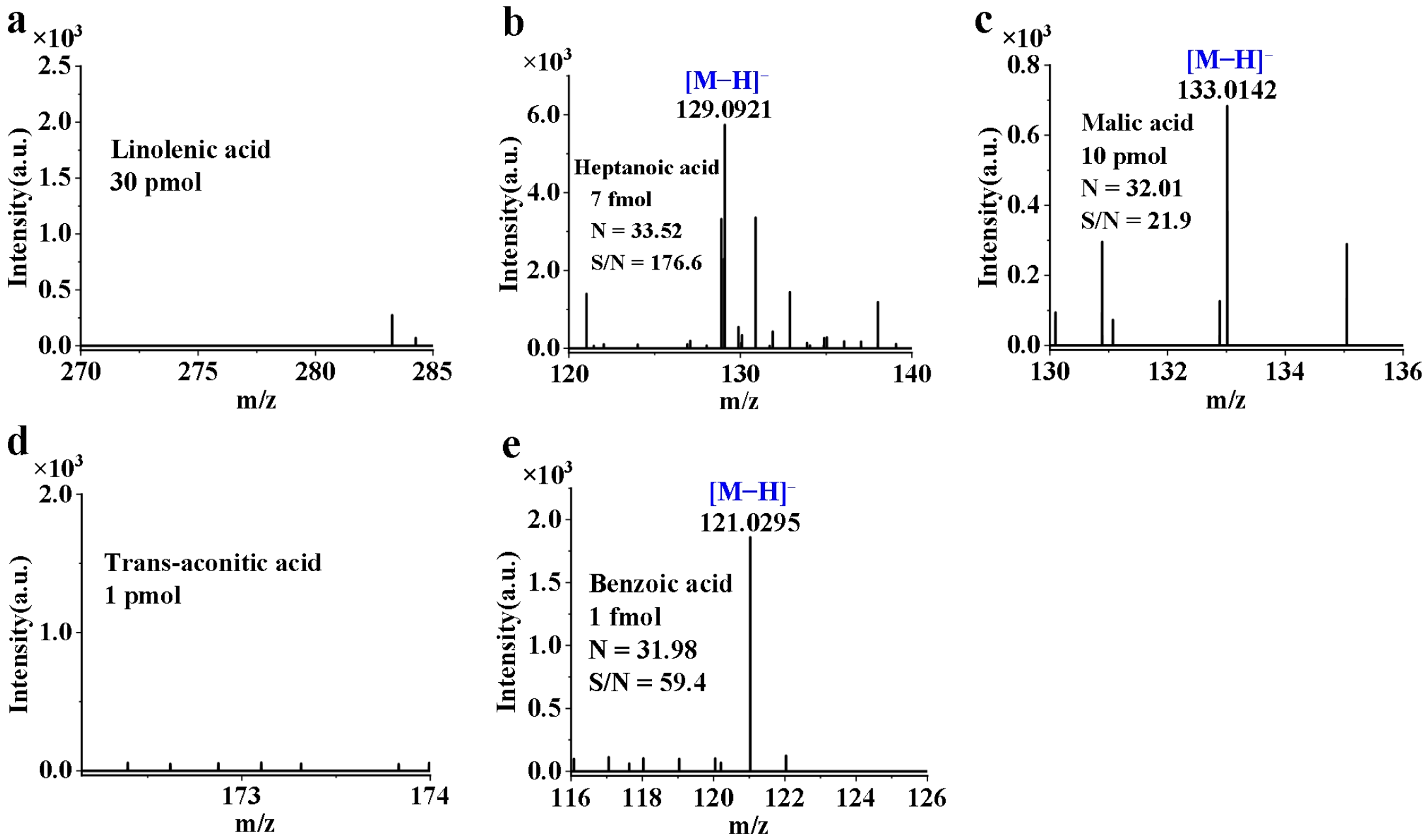

Figure S7. Sensitivity assessment of NEDC-assisted LDI-MS for linolenic acid, heptanoic acid, malic acid, trans-aconitic acid, and benzoic acid.

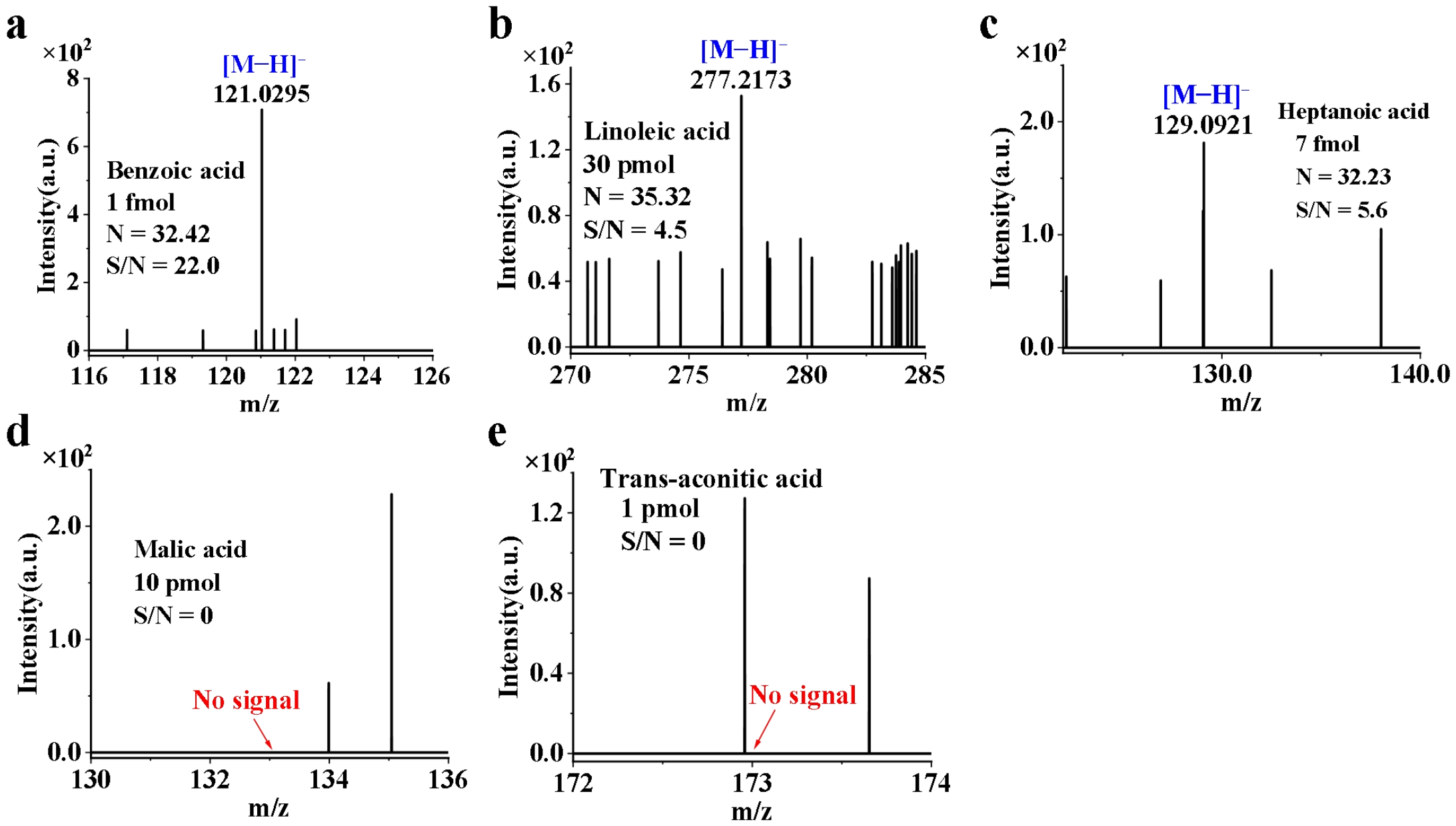

Figure S8. Sensitivity assessment of anti-reflective surface-assisted LDI-MS for linolenic acid, heptanoic acid, malic acid, trans-aconitic acid, and benzoic acid.

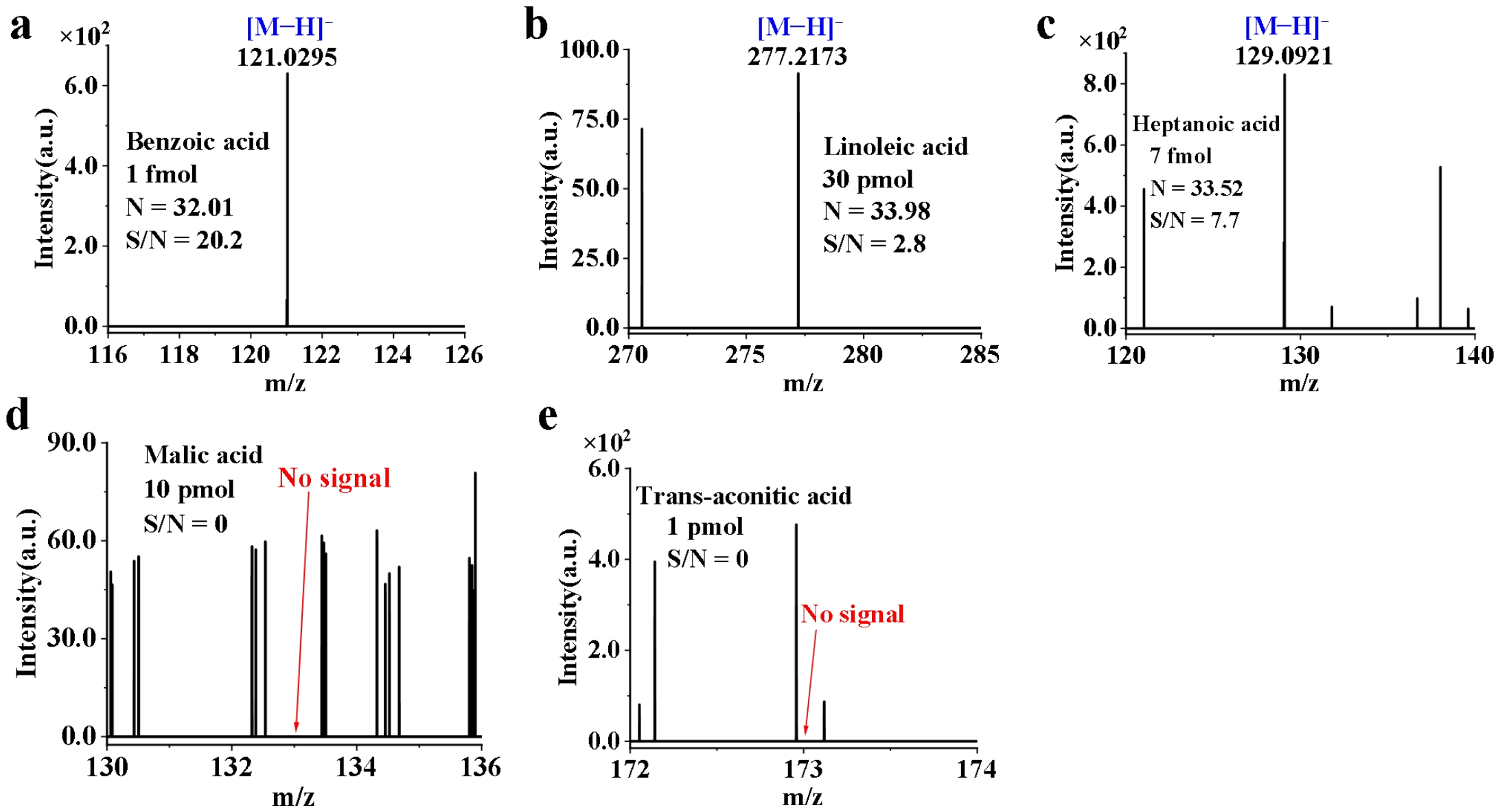

Figure S9. Sensitivity assessment of porous silicon-assisted LDI-MS for linolenic acid, heptanoic acid, malic acid, trans-aconitic acid, and benzoic acid.

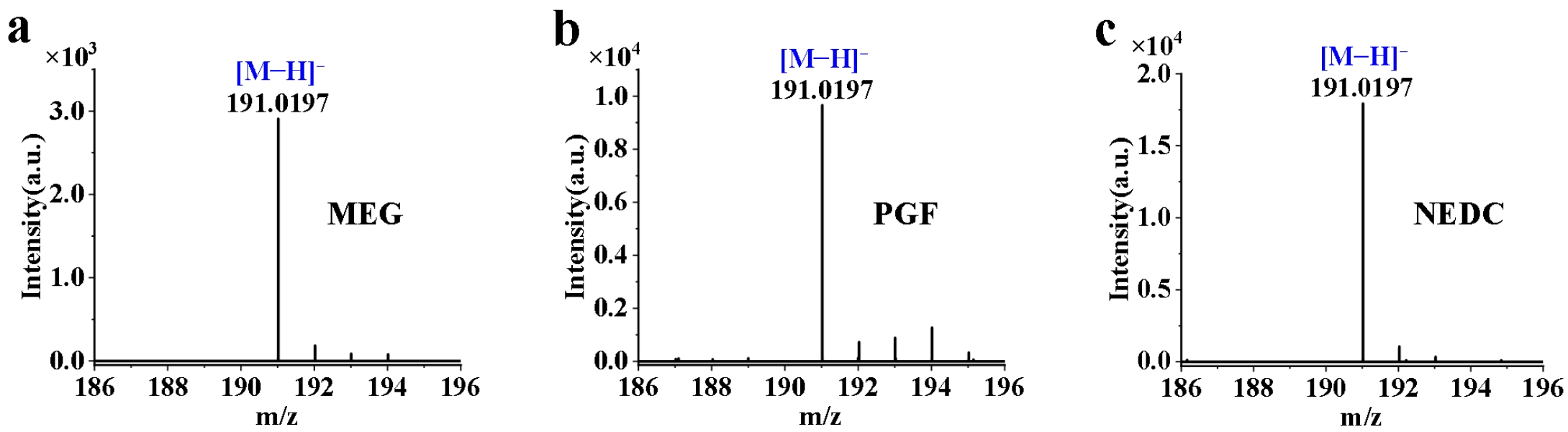

Figure S10. LDI-MS spectra of 1 nmol citric acid acquired at a laser energy of 1.92 μJ/pulse using (a) MEG, (b) PGF, and (c) NEDC as the substrate/matrix.

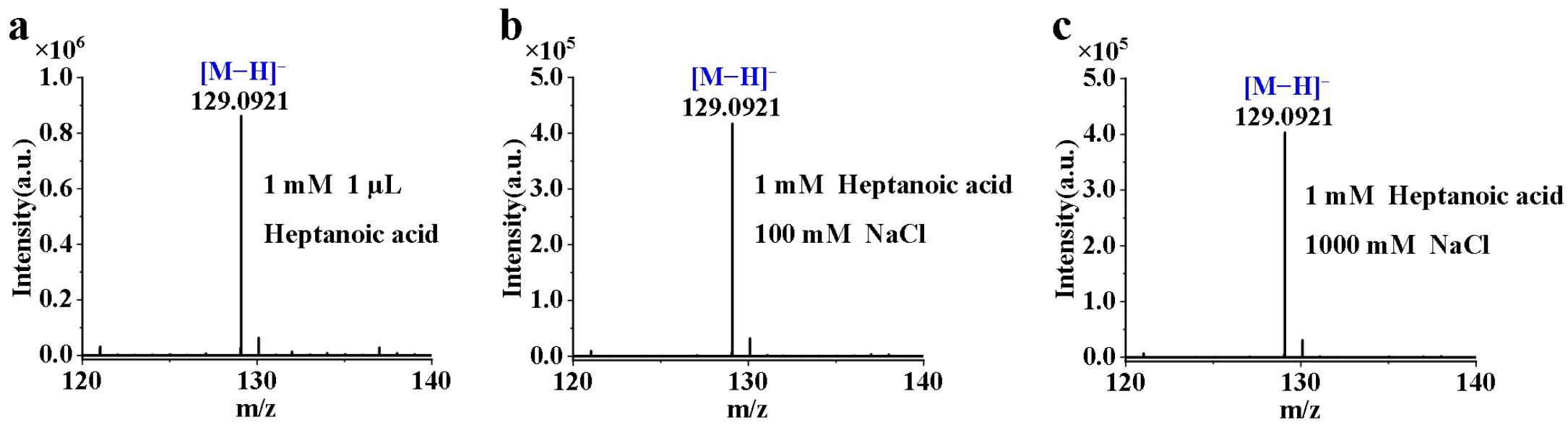

Figure S11. LIGF-LDI-MS spectra of 1 mM heptanoic acid in (a) 0 mM, (b) 100 mM, and (c) 1000 mM NaCl solutions.

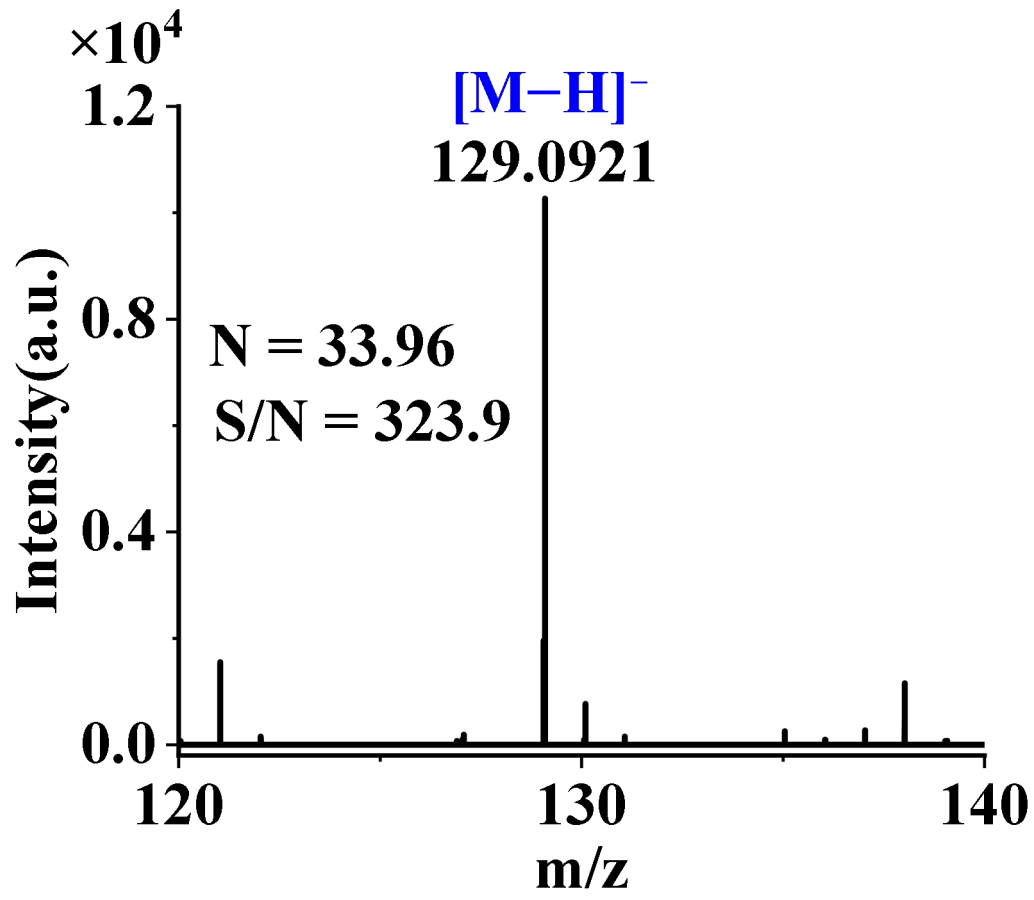

Figure S12. NEDC-assisted LDI-MS spectrum of 1 mM heptanoic acid in a saturated NaCl solution.

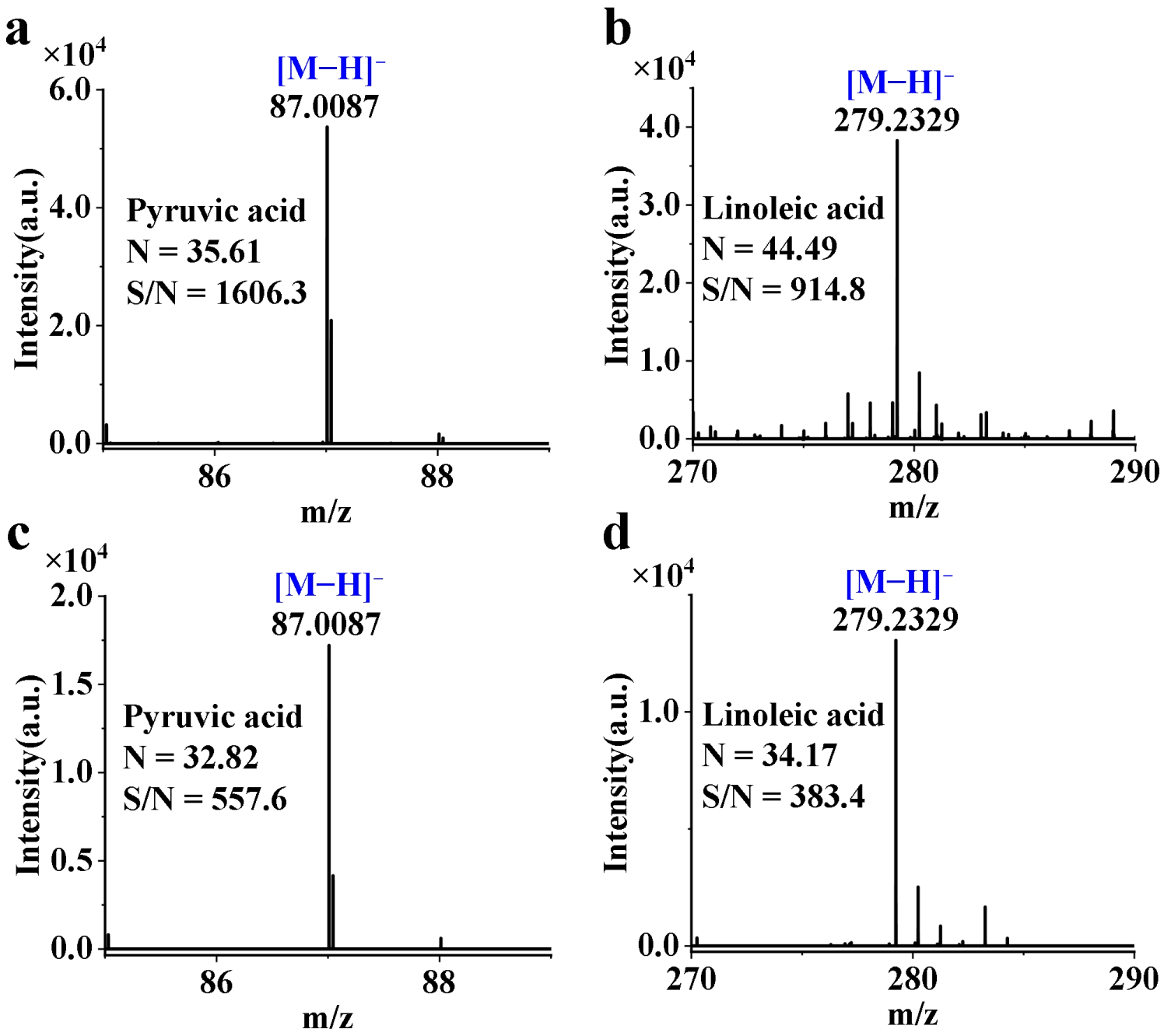

Figure S13. (a), (b) LIGF-LDI-MS and (c), (d) NEDC-assisted LDI-MS spectra of 1 mM pyruvic acid and linoleic acid, respectively, in saturated NaCl solutions.

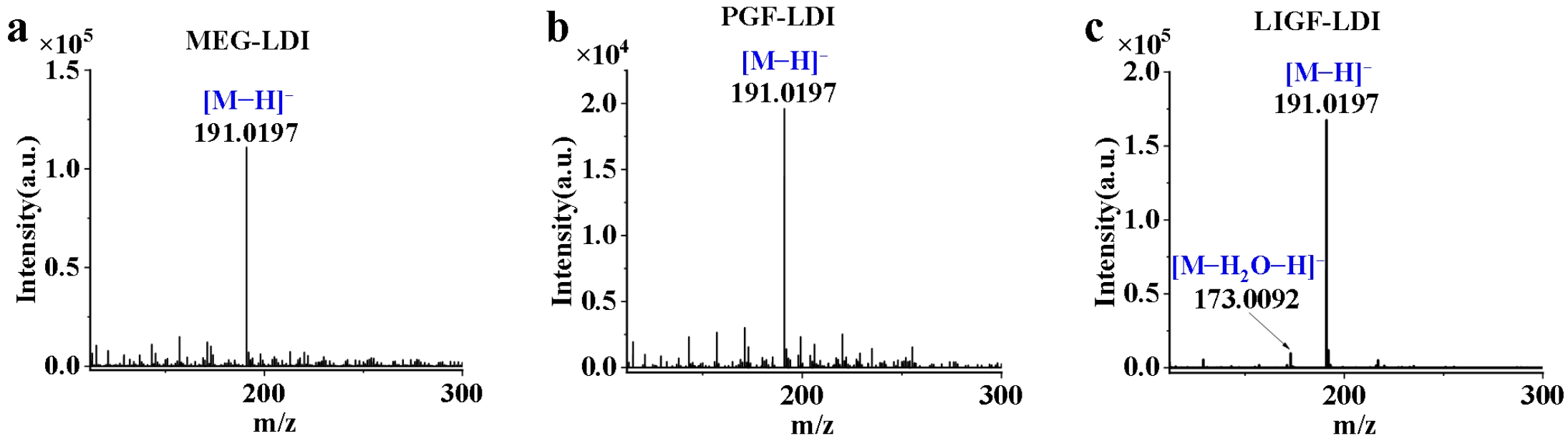

Figure S14. (a) MEG-LDI-MS, (b) PGF-LDI-MS, and (c) LIGF-LDI-MS spectra of 1 nmol citric acid.

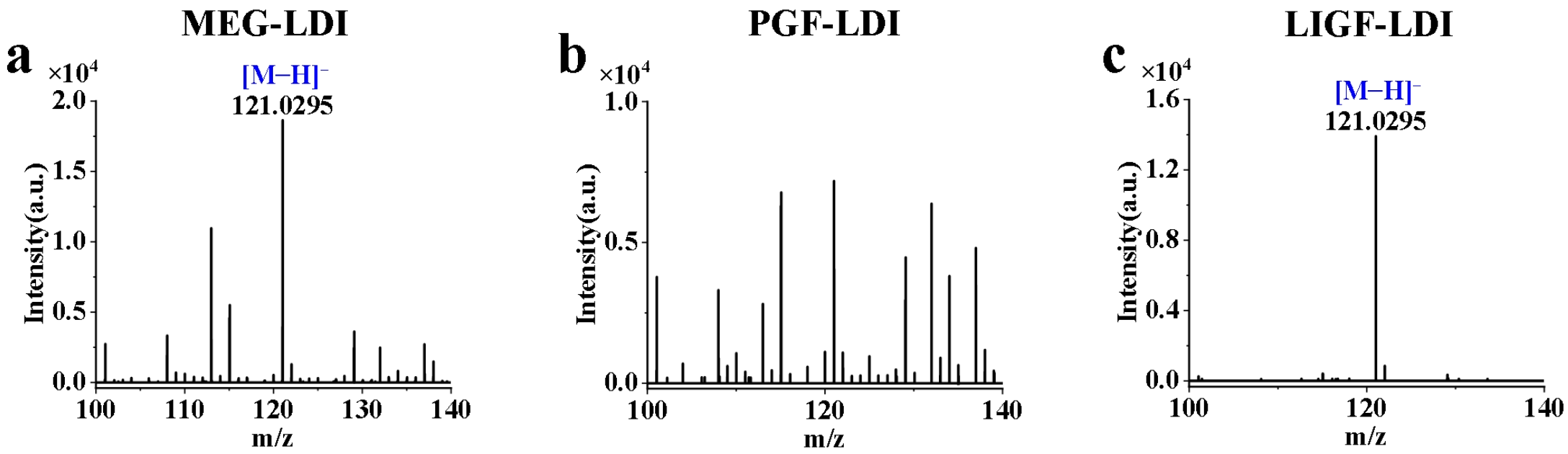

Figure S15. (a) MEG-LDI-MS, (b) PGF-LDI-MS, and (c) LIGF-LDI-MS spectra of 1 nmol benzoic acid.

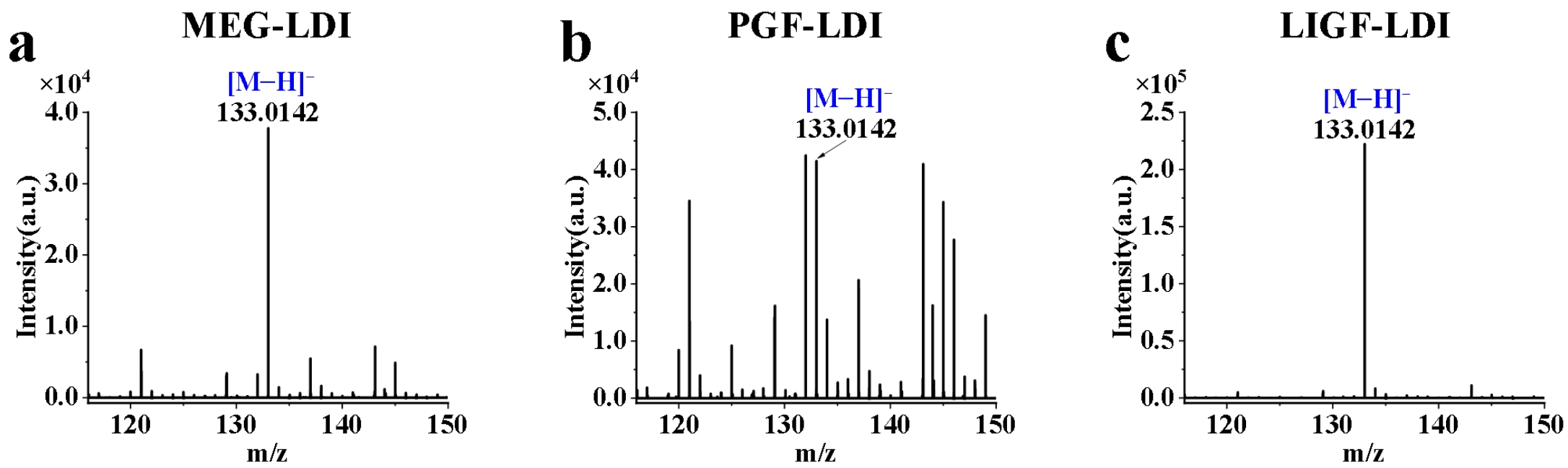

Figure S16. (a) MEG-LDI-MS, (b) PGF-LDI-MS, and (c) LIGF-LDI-MS spectra of 1 nmol malic acid.

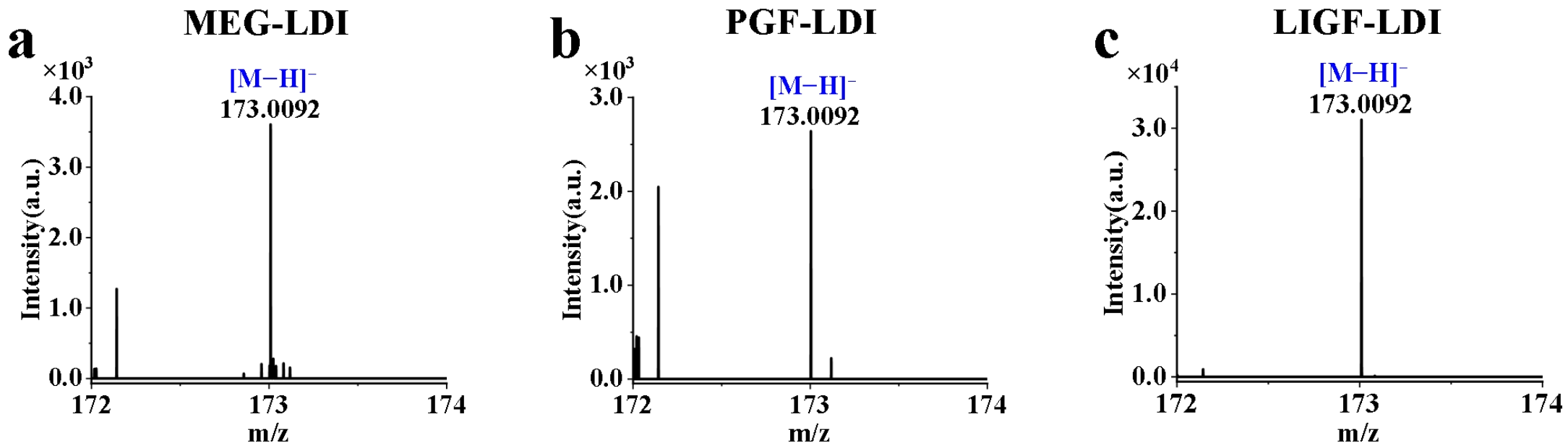

Figure S17. (a) MEG-LDI-MS, (b) PGF-LDI-MS, and (c) LIGF-LDI-MS spectra of 1 nmol trans-aconitic acid.

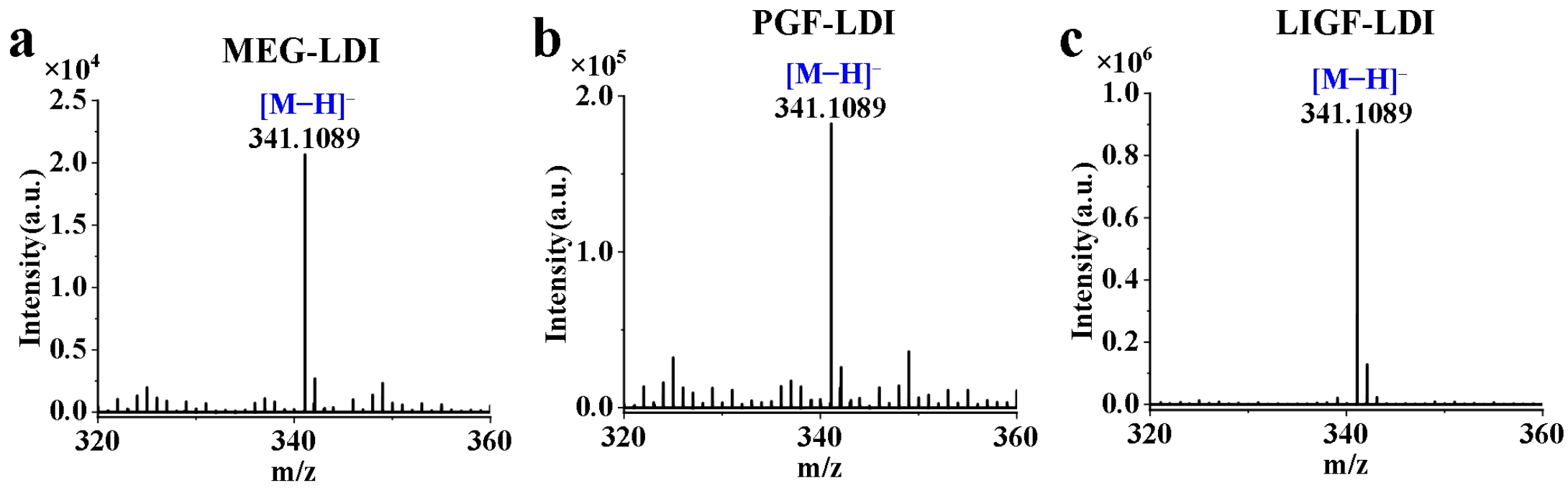

Figure S18. (a) MEG-LDI-MS, (b) PGF-LDI-MS, and (c) LIGF-LDI-MS spectra of 1 nmol sucrose.

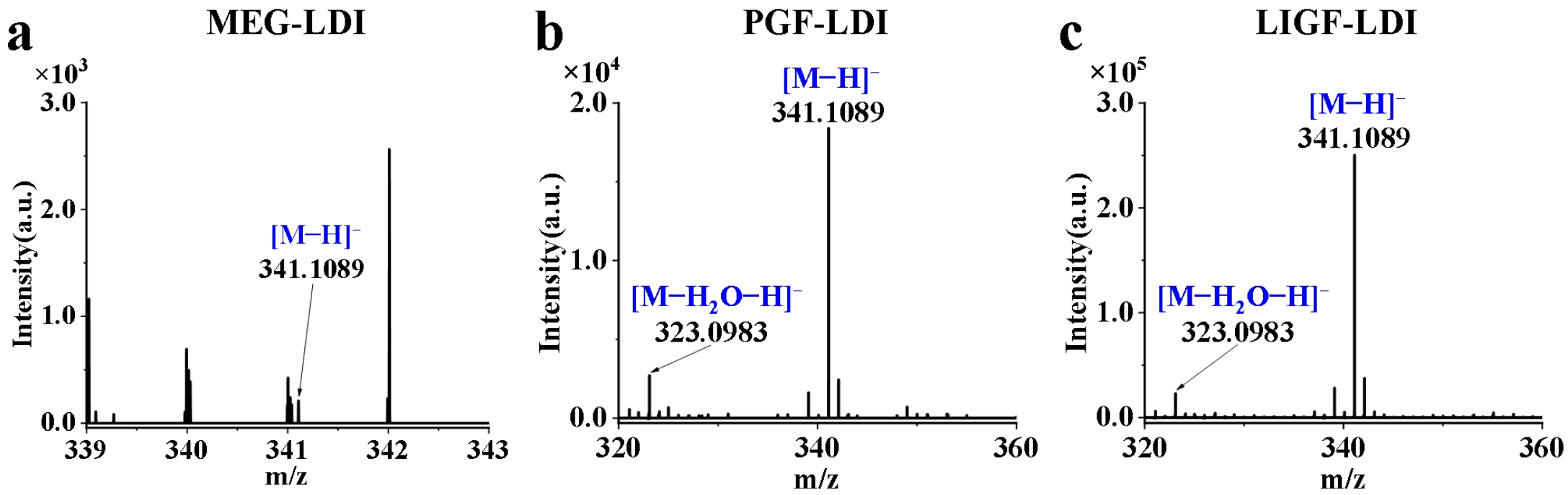

Figure S19. (a) MEG-LDI-MS, (b) PGF-LDI-MS, and (c) LIGF-LDI-MS spectra of 1 nmol maltose.

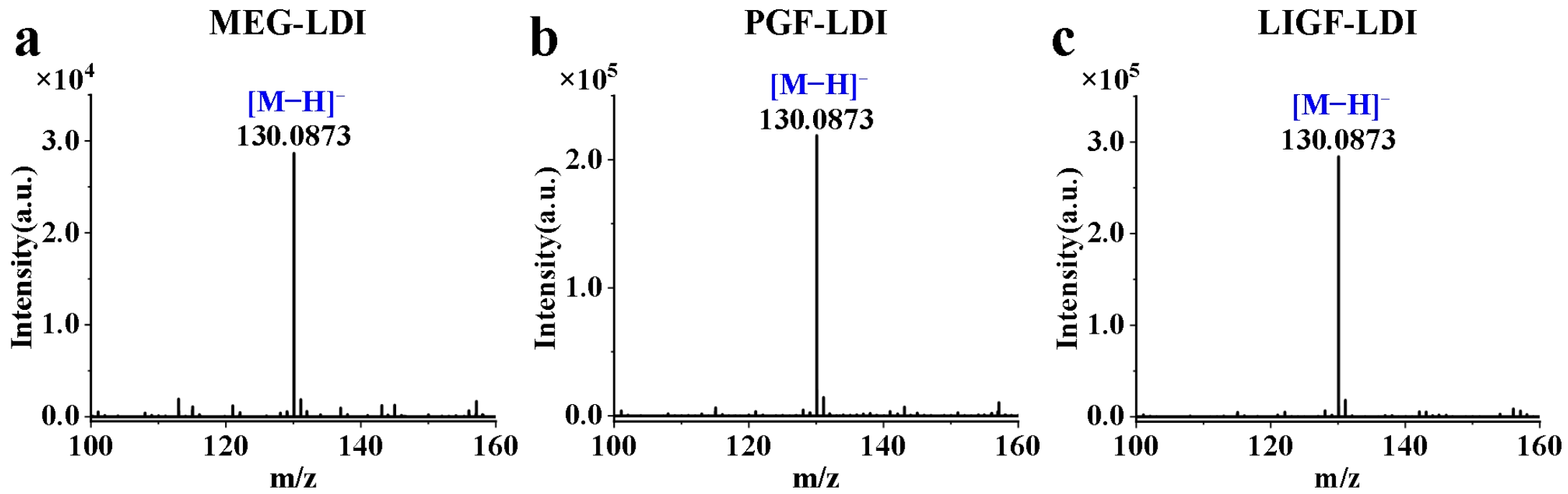

Figure S20. (a) MEG-LDI-MS, (b) PGF-LDI-MS, and (c) LIGF-LDI-MS spectra of 10 nmol L-isoleucine.

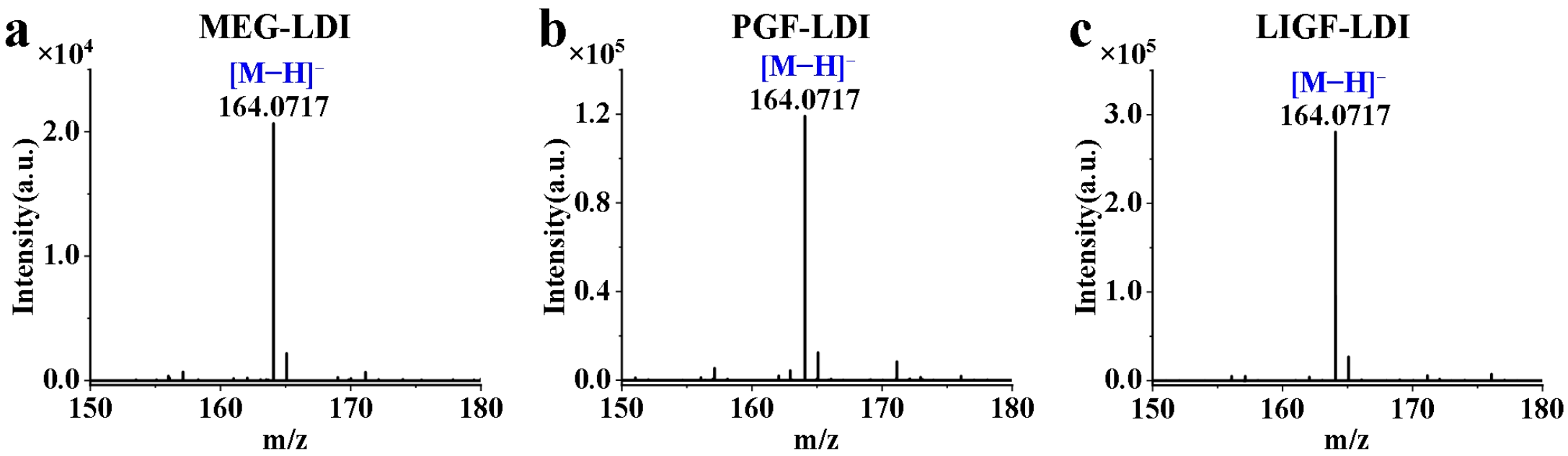

Figure S21. (a) MEG-LDI-MS, (b) PGF-LDI-MS, and (c) LIGF-LDI-MS spectra of 10 nmol L-phenylalanine.

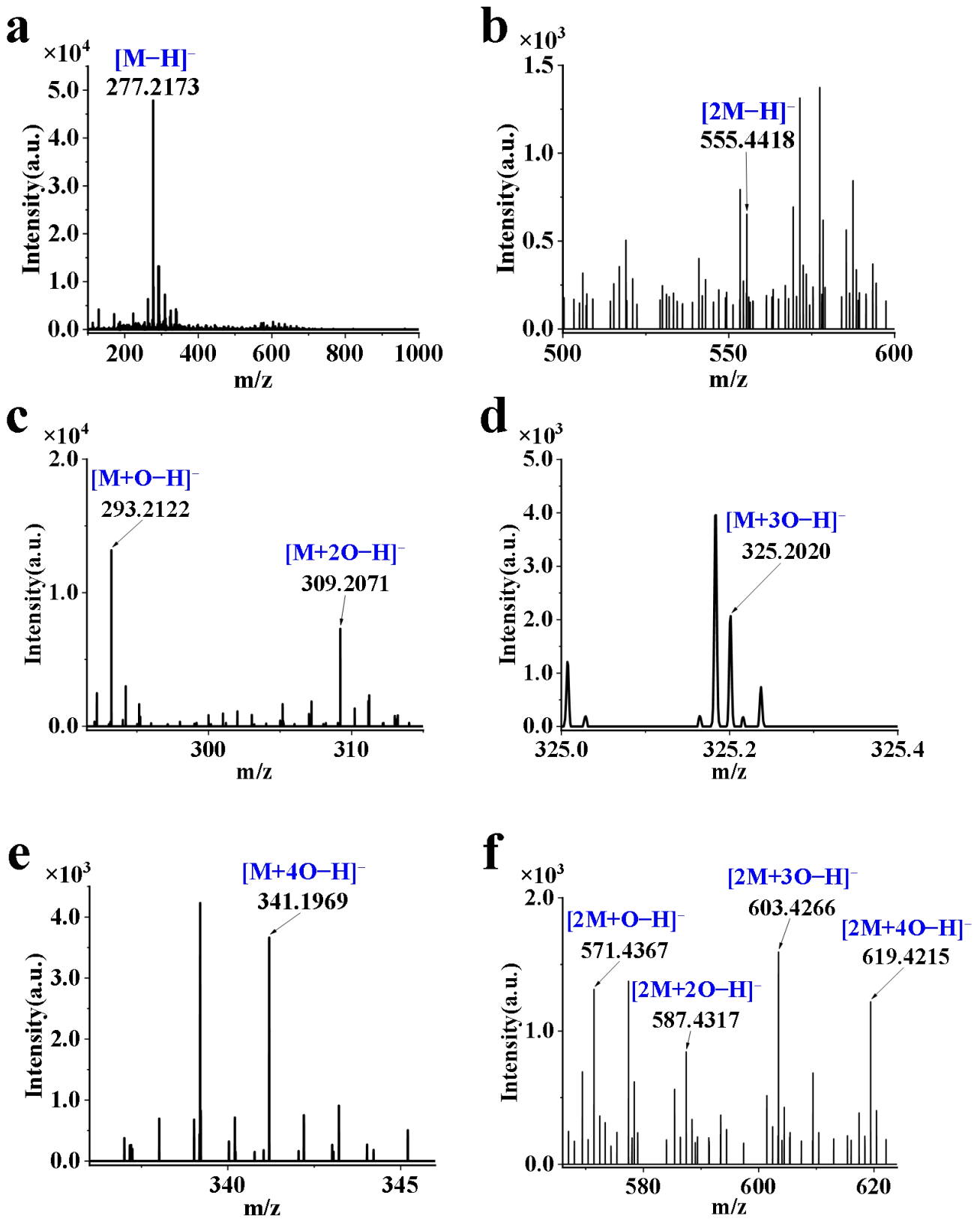

Figure S22. MEG-LDI-MS spectra of 1 mg/mL linolenic acid (1 μL). Magnified views in different m/z ranges for panels (b)–(f).

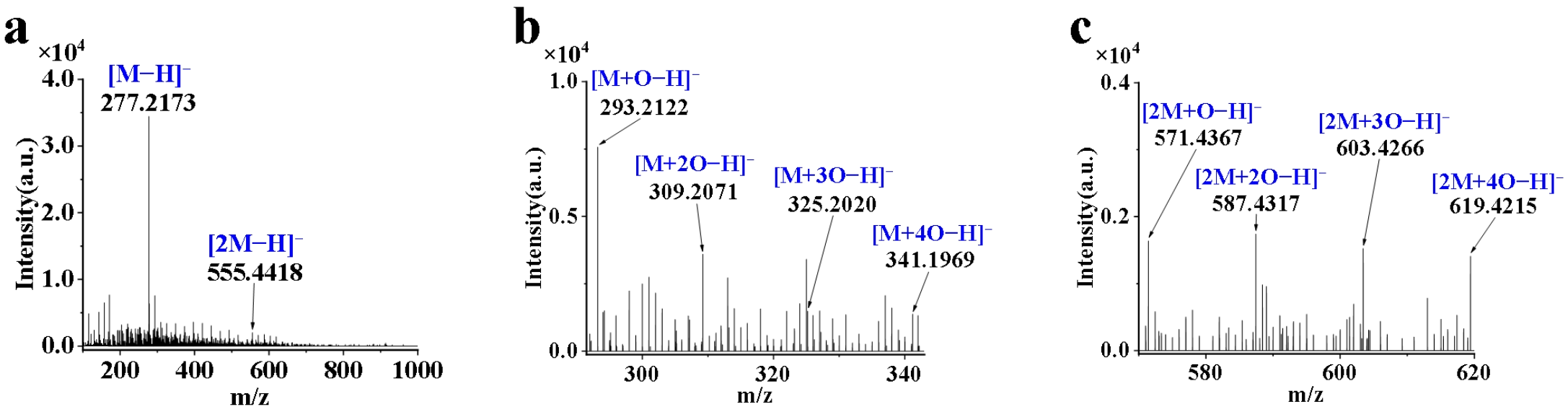

Figure S23. PGF-LDI-MS spectra of 1 mg/mL linolenic acid (1 μL). Magnified views in different m/z ranges for panels (b) and (c).

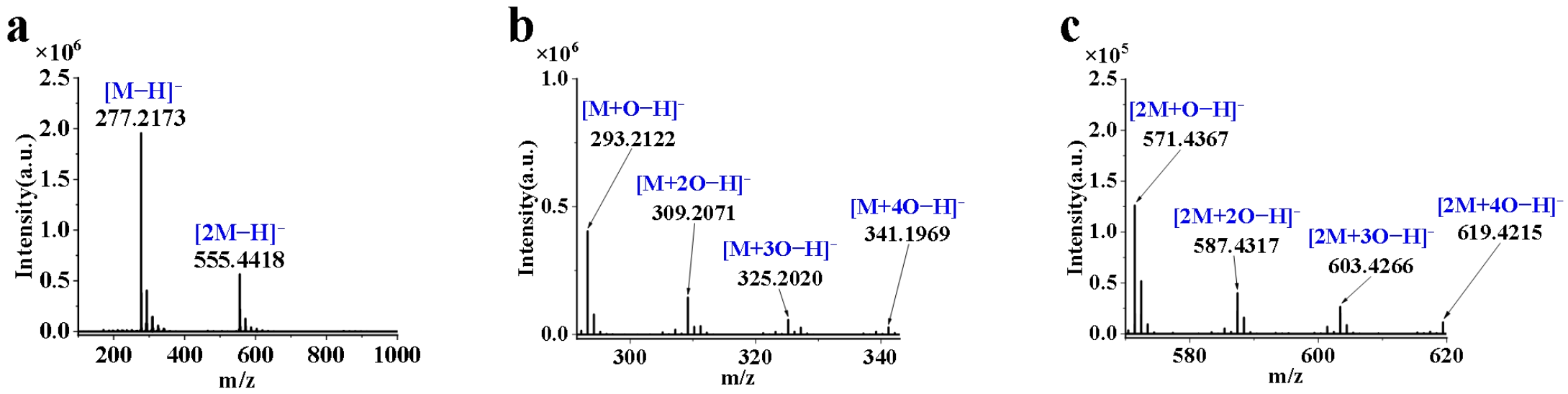

Figure S24. LIGF-LDI-MS spectra of 1 mg/mL linolenic acid (1 μL). Magnified views in different m/z ranges for panels (b) and (c).

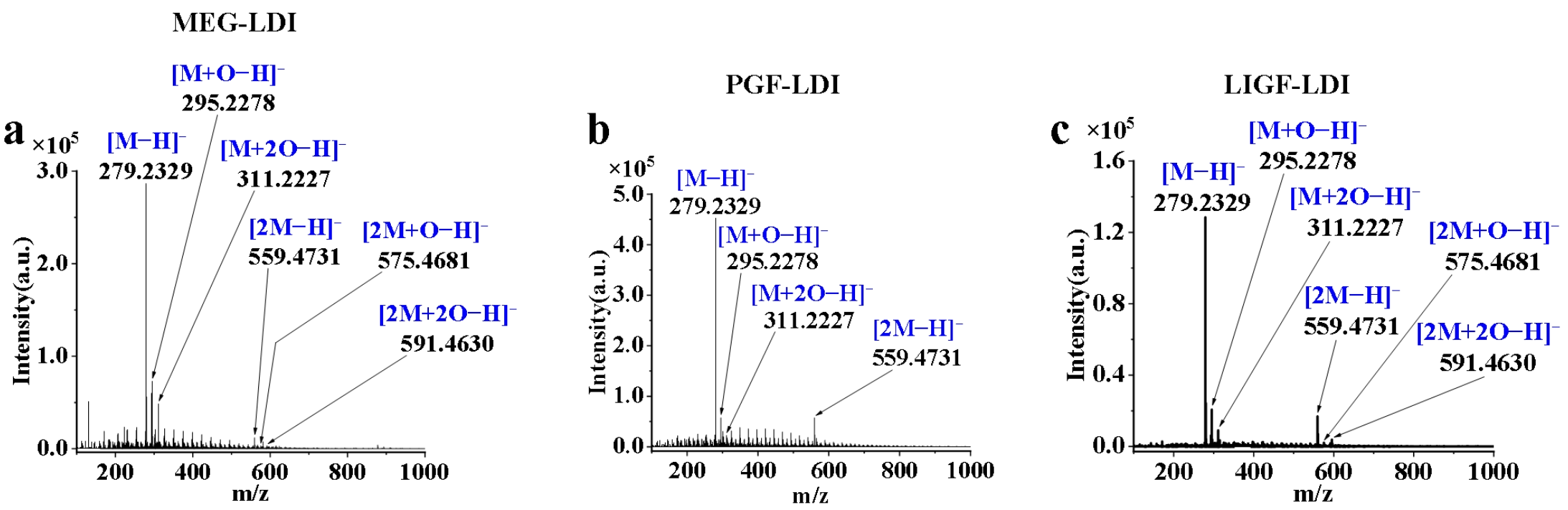

Figure S25. (a) MEG-LDI-MS, (b) PGF-LDI-MS, and (c) LIGF-LDI-MS spectra of 1 mg/mL linoleic acid (1 μL).

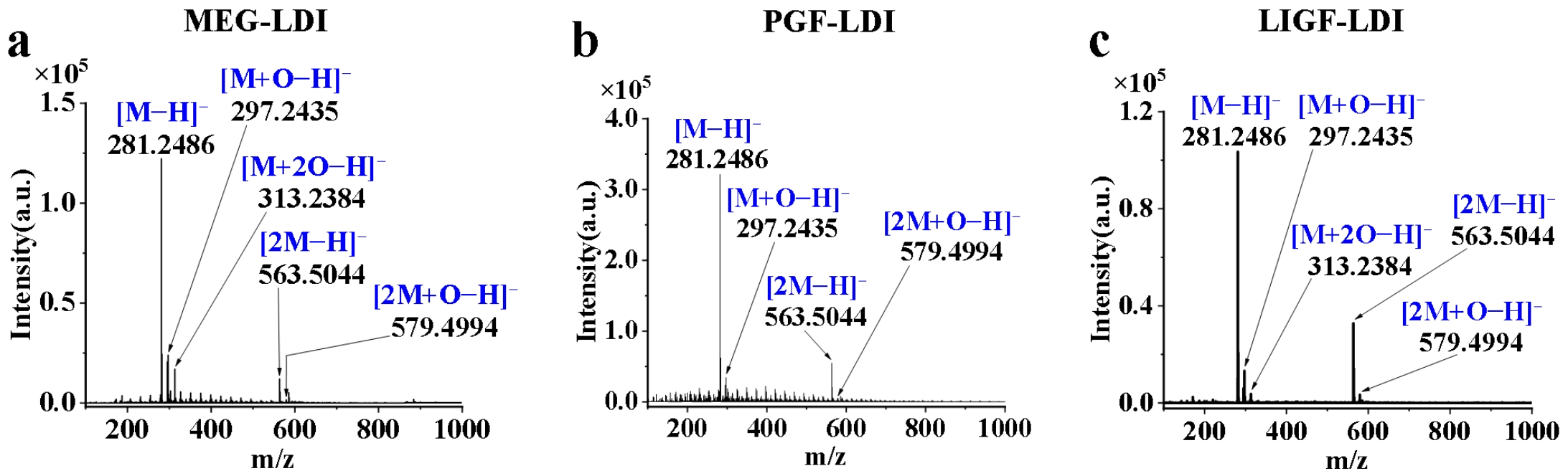

Figure S26. (a) MEG-LDI-MS, (b) PGF-LDI-MS, and (c) LIGF-LDI-MS spectra of 1 mg/mL oleic acid (1 μL).

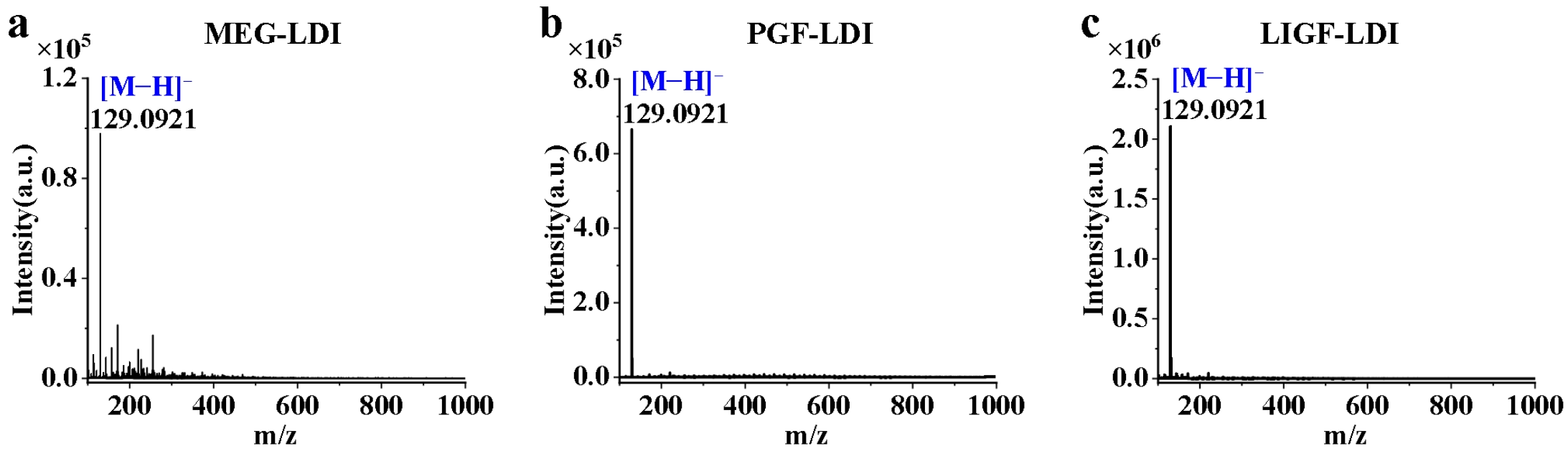

Figure S27. (a) MEG-LDI-MS, (b) PGF-LDI-MS, and (c) LIGF-LDI-MS spectra of 1 mg/mL heptanoic acid (1 μL).

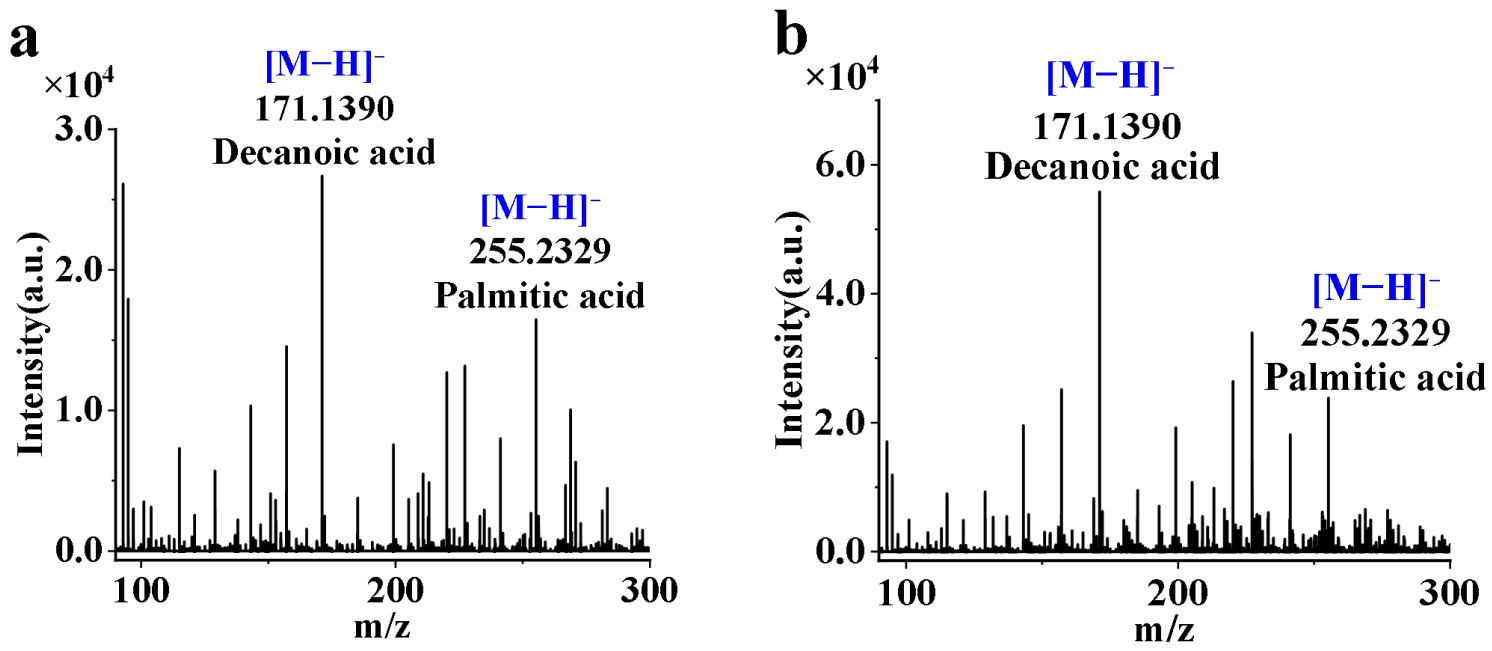

Figure S28. LIGF-LDI-MS spectra of MCF-7 cells and HT-1080 cells.

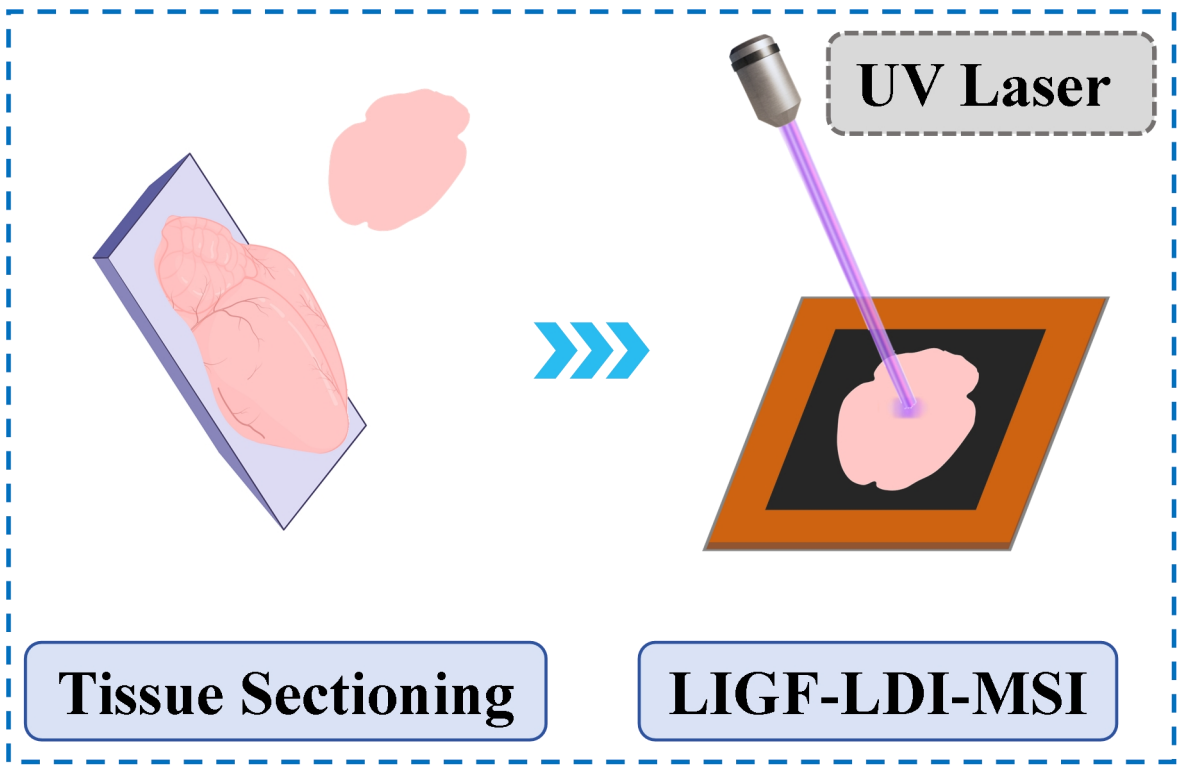

Figure S29. Schematic diagram of the LIGF-LDI-MSI.

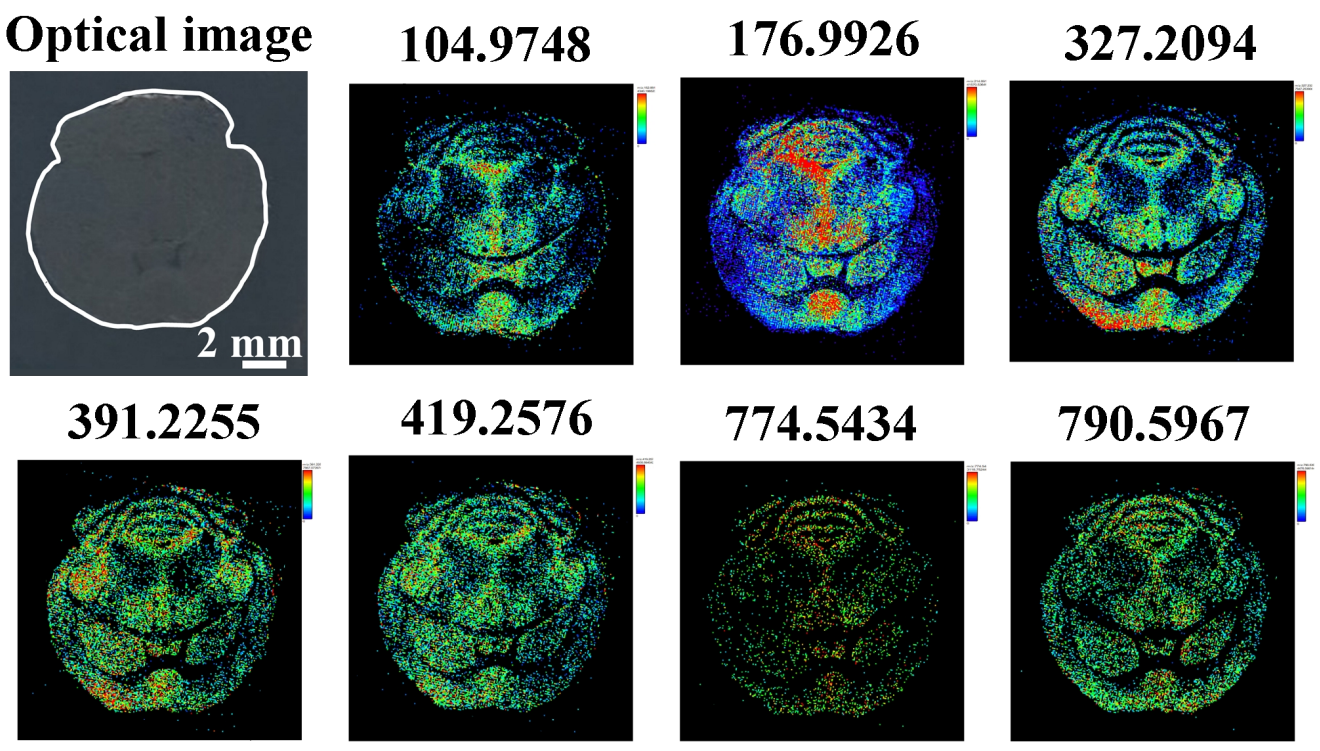

Figure S30. LIGF-LDI-MSI images of a mouse brain tissue section acquired in linear scanning mode. Scan speed: 3.8 px/s; spatial resolution: 50 μm; laser energy: 11.2 μJ/pulse.

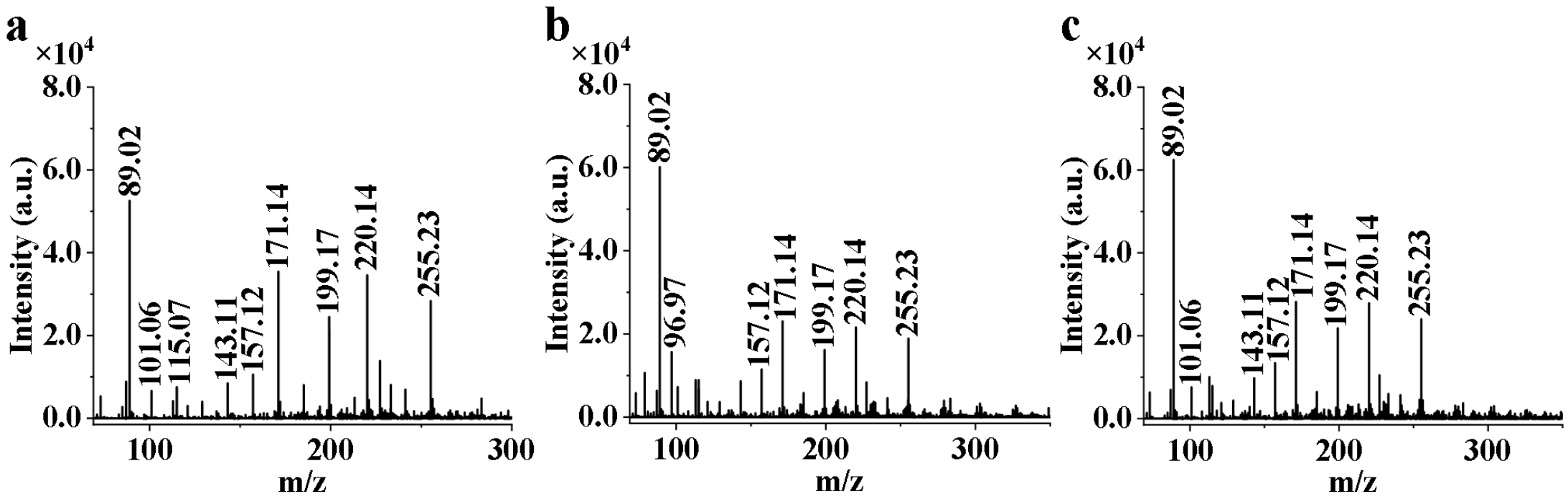

Figure S31. Representative LIGF-LDI-MS spectra of (a) mouse liver tissue and (b) mouse brain tissue acquired in 3D mode. (c) Representative LIGF-LDI-MS spectra of mouse brain tissue acquired in linear scanning mode.

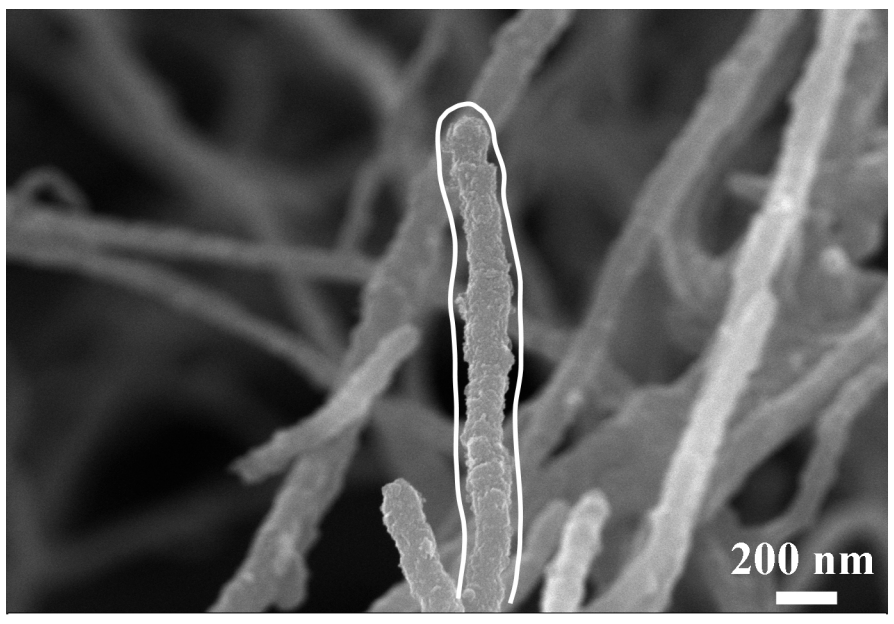

Figure S32. SEM image of the LIGF tip morphology.

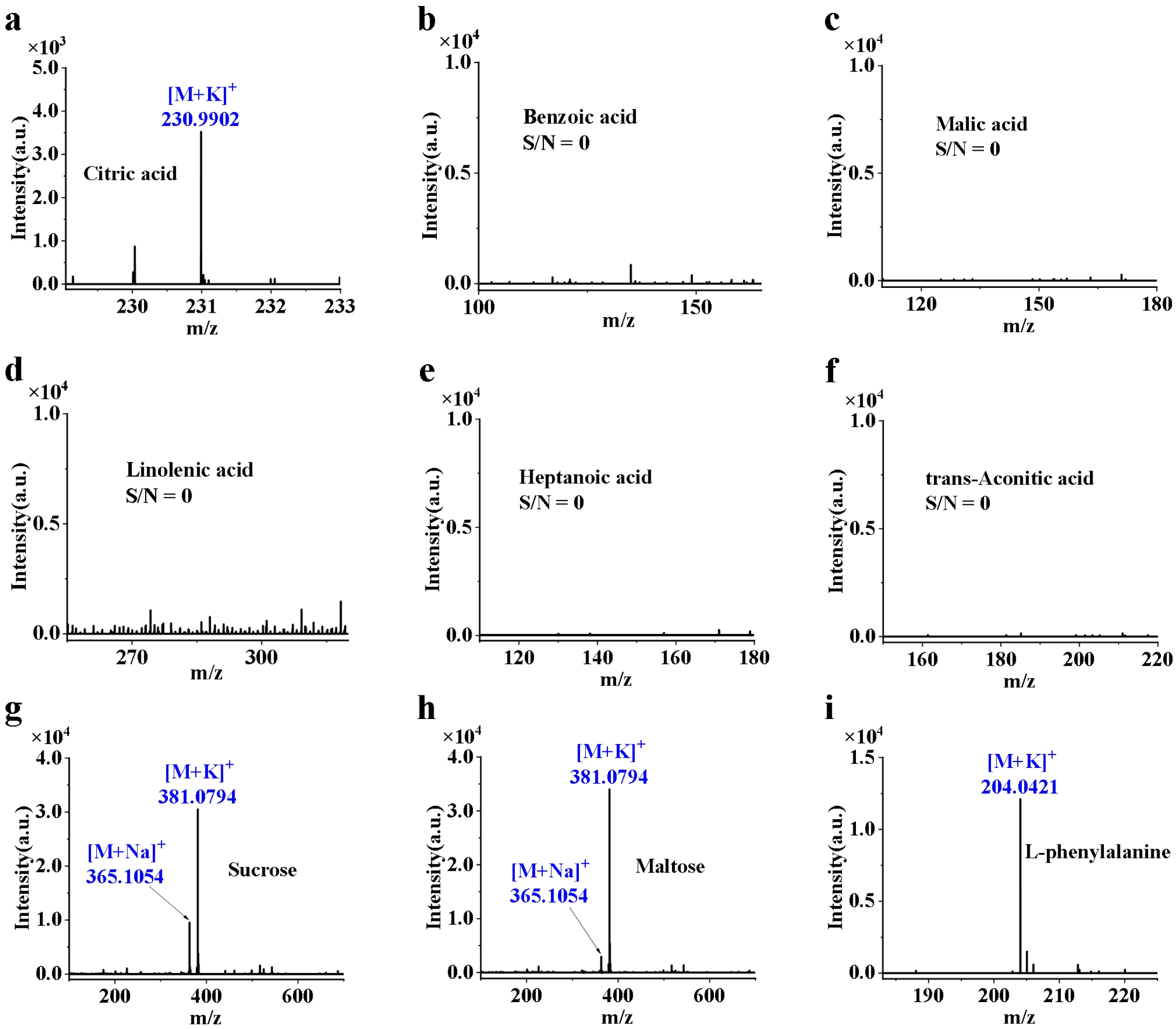

Figure S33. LIGF-LDI-MS spectra of nine standard analytes in positive ion mode: (a) citric acid, (b) benzoic acid, (c) malic acid, (d) linolenic acid, (e) heptanoic acid, (f) trans-aconitic acid, (g) sucrose, (h) maltose, and (i) L-phenylalanine. The loading of each analyte was the same as that used in negative-ion mode.

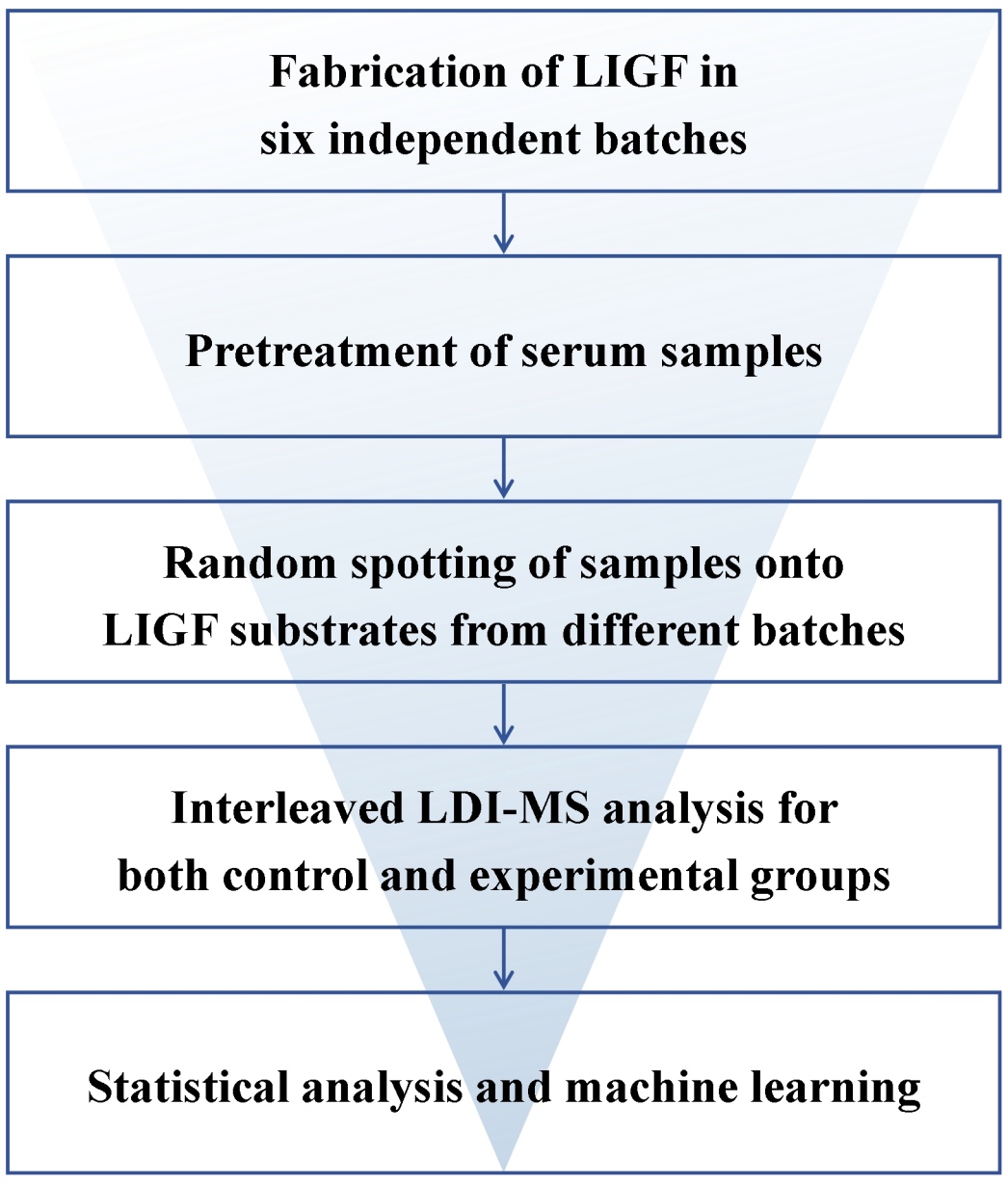

Figure S34. Experimental workflow for clinical serum sample analysis using LIGF-LDI-MS.

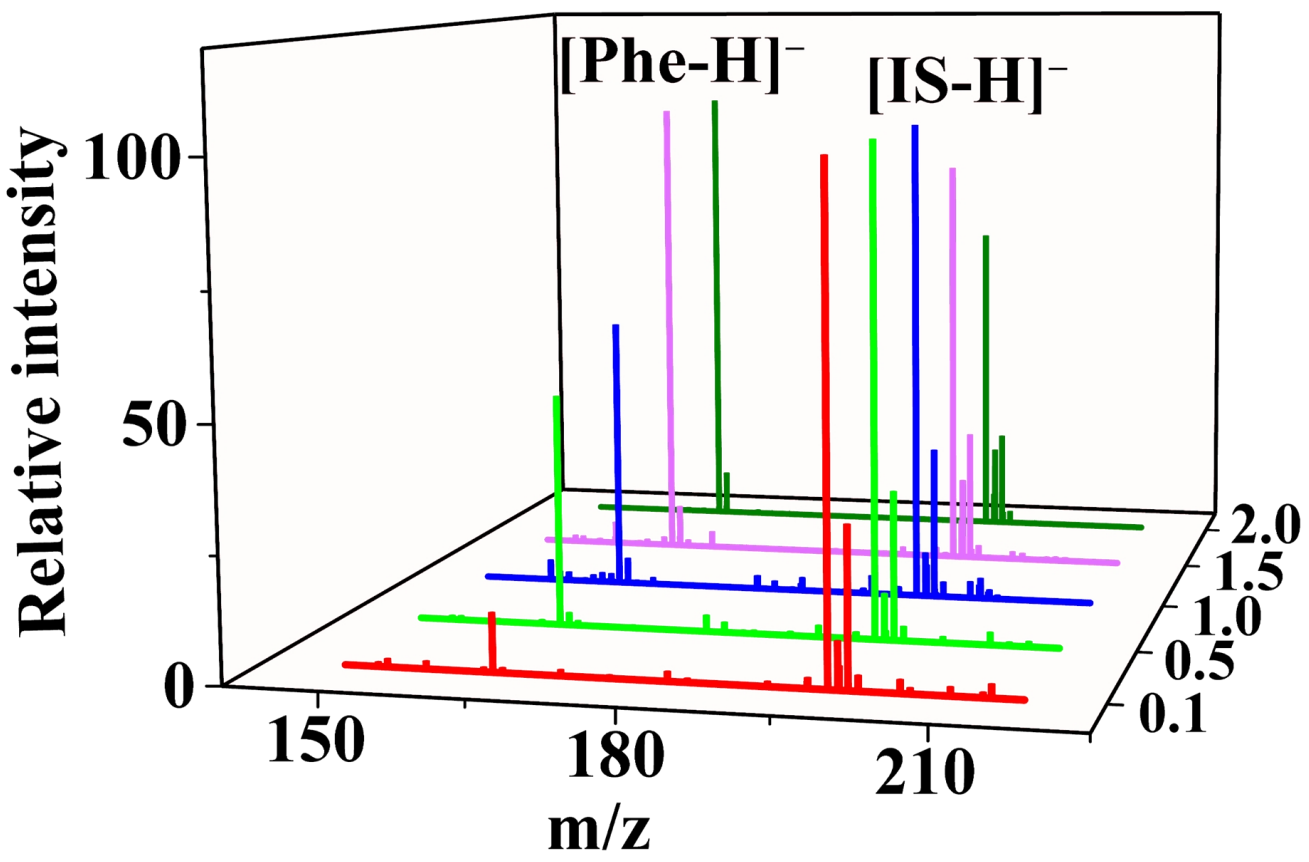

Figure S35. Overlaid LIGF-LDI-MS spectra of L-phenylalanine and the internal standard (IS) at different concentration ratios (0.1-2.0).

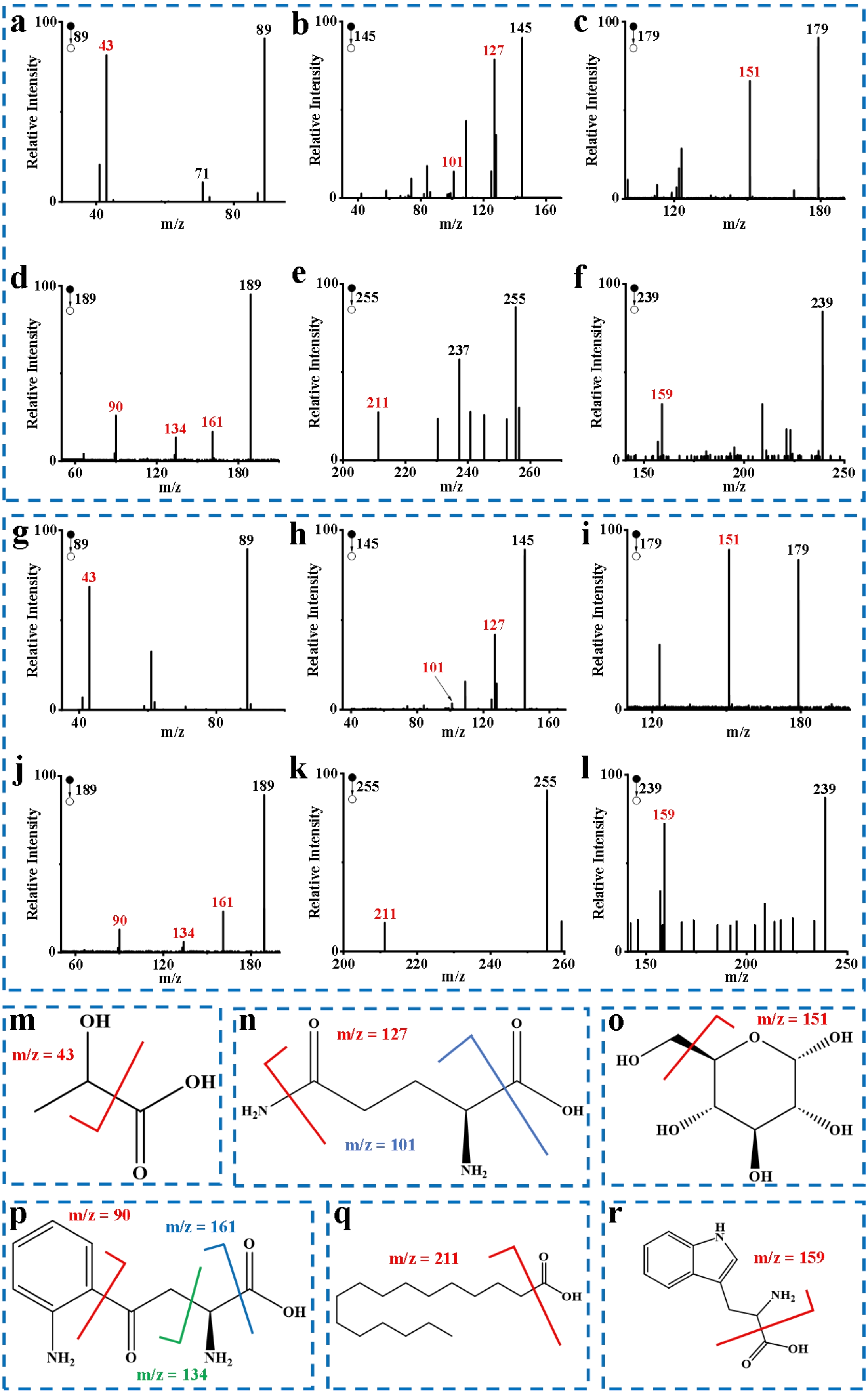

Figure S36. MS/MS spectra of six key metabolites (*m/z*: 89.02, 255.23, 189.07, 239.06, 179.06, and 145.06). (a–f) MS/MS spectra of six standards. (g–l) MS/MS spectra of six metabolites in serum.

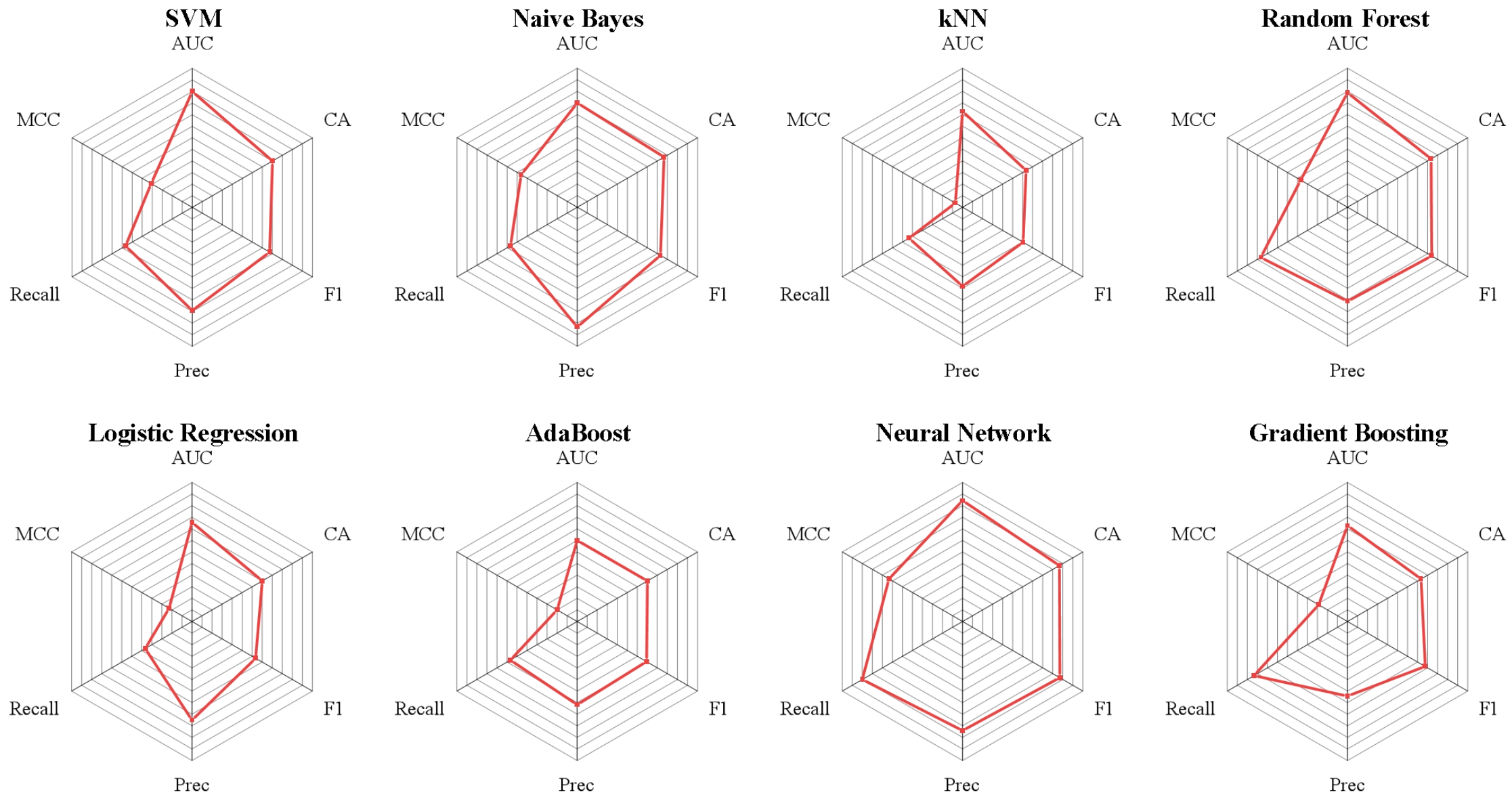

Figure S37. Comparison between eight machine learning models.

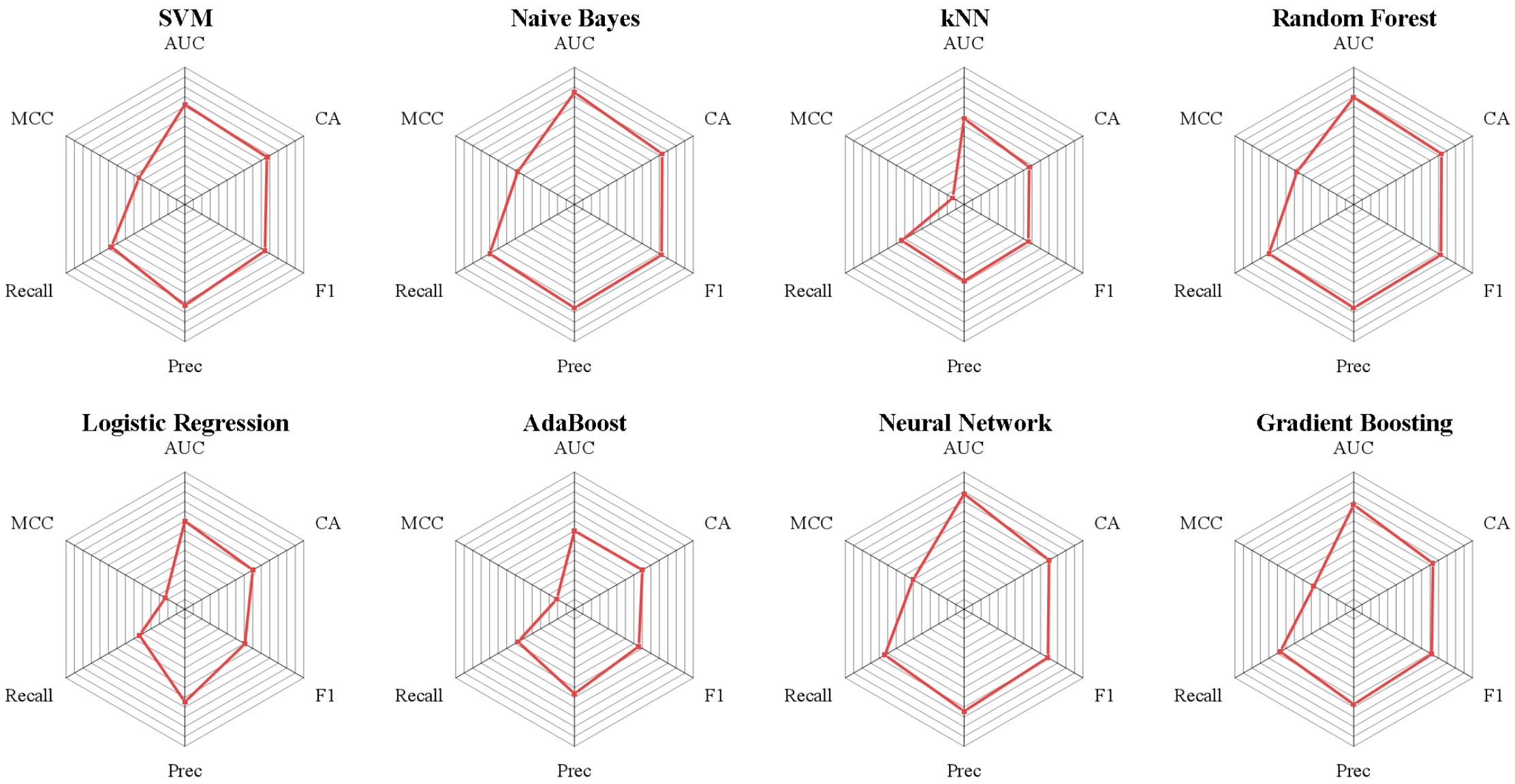

Figure S38. Comparison between eight machine learning models using six selected metabolites.

Table S1. Elemental composition of the LIGF.

Element	Signal Type	At%
C	EDS	95.28
N	EDS	0.00
O	EDS	4.72
Total		100.00

Table S2. List of metabolites identified in mouse brain and liver tissues.

m/z_{cal}	m/z_{exp}	$\Delta m/z$ (ppm)	Assignment	Ion	Formula
104.9747	104.9748	-1.0	Phosphonoacetaldehyde	$[M-H_2O-H]^-$	$C_2H_5O_4P$
176.9918	176.9926	-4.5	Orotic acid	$[M+Na-2H]^-$	$C_5H_4N_2O_4$
327.2096	327.2094	0.6	5α-Androstane-3β,17β-diol	$[M+Cl]^-$	$C_{19}H_{32}O_2$
391.2255	391.2255	0.0	LPA(16:0)	$[M-H_2O-H]^-$	$C_{19}H_{39}O_7P$
419.2568	419.2576	-1.9	LPA(18:0)	$[M-H_2O-H]^-$	$C_{21}H_{43}O_7P$
762.4846	762.4869	-3.0	PC(32:3)	$[M+Cl]^-$	$C_{40}H_{74}NO_8P$
774.5443	774.5434	1.2	PE(40:5)	$[M-H_2O-H]^-$	$C_{45}H_{80}NO_8P$
790.5969	790.5967	0.3	GlcCer(d18:1/20:0)	$[M+Cl]^-$	$C_{44}H_{85}NO_8$

Table S3. Comparison of classification performance of various machine learning models on the full dataset.

Modle	AUC	CA	F1	Prec	Recall	MCC
**AdaBoost**	0.750	0.750	0.746	0.759	0.733	0.500
**Gradient Boosting**	0.814	0.767	0.788	0.722	0.867	0.544
**kNN**	0.814	0.717	0.702	0.741	0.667	0.436
**Logistic Regression**	0.828	0.750	0.717	0.826	0.633	0.514
**Naive Bayes**	0.850	0.833	0.815	0.917	0.733	0.680
**Neural Network**	0.924	0.883	0.885	0.871	0.900	0.767
**Random Forest**	0.894	0.817	0.820	0.806	0.833	0.634
**SVM**	0.900	0.800	0.786	0.846	0.733	0.605

Table S4. Comparison of classification performance of various machine learning models on the dataset derived from the six representative differential metabolites.

Modle	AUC	CA	F1	Prec	Recall	MCC
**AdaBoost**	0.700	0.700	0.679	0.731	0.633	0.404
**Gradient Boosting**	0.833	0.767	0.759	0.786	0.733	0.535
**kNN**	0.739	0.683	0.678	0.690	0.667	0.367
**Logistic Regression**	0.750	0.700	0.654	0.773	0.567	0.415
**Naive Bayes**	0.872	0.817	0.814	0.828	0.800	0.634
**Neural Network**	0.885	0.800	0.793	0.821	0.767	0.601
**Random Forest**	0.847	0.817	0.814	0.828	0.800	0.634
**SVM**	0.811	0.783	0.772	0.815	0.733	0.570

Table S5. Tentatively Identified compounds from the clinical serum samples.

m/z_{cal}	m/z_{exp}	$\Delta m/z$ (ppm)	Assignment	Ion	Formula
57.0346	57.0345	1.6481	Acetone	$[M-H]^-$	C_3H_6O
59.0139	59.0138	1.0845	Acetic acid	$[M-H]^-$	$C_2H_4O_2$
68.9982	68.9981	1.0725	Pyruvic acid	$[M-H_2O-H]^-$	$C_3H_4O_3$
69.0346	69.0344	2.5060	Butyric acid	$[M-H_2O-H]^-$	$C_4H_8O_2$
71.0139	71.0138	1.2110	Lactic acid	$[M-H_2O-H]^-$	$C_3H_6O_3$
72.9931	72.9930	1.0138	Glyoxylic acid	$[M-H]^-$	$C_2H_2O_3$
73.0295	73.0294	0.7668	Glycerol	$[M-H_2O-H]^-$	$C_3H_8O_3$
74.0248	74.0246	2.2290	Glycine	$[M-H]^-$	$C_2H_5NO_2$
75.0088	75.0086	1.7731	Glycolic acid	$[M-H]^-$	$C_2H_4O_3$
83.0139	83.0138	0.2650	Acetoacetic acid	$[M-H_2O-H]^-$	$C_4H_6O_3$
83.0502	83.0502	0.7586	Valeric acid	$[M-H_2O-H]^-$	$C_5H_{10}O_2$
85.0295	85.0295	-0.0118	3-Hydroxybutyric acid (3-HB)	$[M-H_2O-H]^-$	$C_4H_8O_3$
86.0248	86.0247	0.3487	Serine	$[M-H_2O-H]^-$	$C_3H_7NO_3$
87.0088	87.0088	-0.2988	Pyruvic acid	$[M-H]^-$	$C_3H_4O_3$
87.0452	87.0451	0.6893	Butyric acid	$[M-H]^-$	$C_4H_8O_2$
88.0404	88.0403	1.1245	L-Alanine	$[M-H]^-$	$C_3H_7NO_2$
89.0244	89.0244	0.4942	Lactic acid	$[M-H]^-$	$C_3H_6O_3$
91.0401	91.0401	-0.7799	Glycerol	$[M-H]^-$	$C_3H_8O_3$
93.0094	93.0094	0.0108	Uracil	$[M-H_2O-H]^-$	$C_4H_4N_2O_2$
95.0251	95.0251	-0.3262	Dihydrouracil	$[M-H_2O-H]^-$	$C_4H_6N_2O_2$
96.9697	96.9695	2.4788	Acetic acid	$[M+K-2H]^-$	$C_2H_4O_2$

97.0295	97.0294	1.0718	α-Ketoisovaleric acid (α-KIV)	$[M-H_2O-H]^-$	$C_5H_8O_3$
98.0014	98.0010	4.2433	Ethanolamine	$[M+K-2H]^-$	C_2H_7NO
99.0088	99.0088	-0.0404	Succinic acid	$[M-H_2O-H]^-$	$C_4H_6O_4$
99.0452	99.0451	0.1111	2-Hydroxyvaleric acid (2-HV)	$[M-H_2O-H]^-$	$C_5H_{10}O_3$
100.0404	100.0404	-0.1399	L-Threonine	$[M-H_2O-H]^-$	$C_4H_9NO_3$
101.0244	101.0243	0.9008	2-Ketobutyric acid (2-KB)	$[M-H]^-$	$C_4H_6O_3$
101.0608	101.0607	0.6036	Valeric acid	$[M-H]^-$	$C_5H_{10}O_2$
103.0401	103.0400	0.3688	3-Hydroxybutyric acid (3-HB)	$[M-H]^-$	$C_4H_8O_3$
104.0353	104.0352	1.5379	Serine	$[M-H]^-$	$C_3H_7NO_3$
110.0248	110.0248	-0.7180	Pyroglutamic acid	$[M-H_2O-H]^-$	$C_5H_7NO_3$
111.0200	111.0202	-1.7114	Uracil	$[M-H]^-$	$C_4H_4N_2O_2$
112.0404	112.0403	0.8122	4-Hydroxyproline	$[M-H_2O-H]^-$	$C_5H_9NO_3$
113.0244	113.0244	-0.1062	Glutaric acid	$[M-H_2O-H]^-$	$C_5H_8O_4$
113.0357	113.0356	0.3539	L-Asparagine	$[M-H_2O-H]^-$	$C_4H_8N_2O_3$
114.0197	114.0196	0.7718	L-Aspartic acid	$[M-H_2O-H]^-$	$C_4H_7NO_4$
114.0561	114.0559	1.0083	Proline	$[M-H]^-$	$C_5H_9NO_2$
115.0765	115.0765	0.0087	Caproic acid	$[M-H]^-$	$C_6H_{12}O_2$
116.0506	116.0506	-0.6032	Indole	$[M-H]^-$	C_8H_7N
116.0717	116.0716	0.4738	L-Valine	$[M-H]^-$	$C_5H_{11}NO_2$
117.0193	117.0192	0.8546	Succinic acid	$[M-H]^-$	$C_4H_6O_4$
117.0346	117.0344	1.3757	Phenylacetic acid (PAA)	$[M-H_2O-H]^-$	$C_8H_8O_2$

117.0557	117.0557	0.5553	3-Hydroxyisovaleric acid (3-HIVA)	$[M-H]^-$	$C_5H_{10}O_3$
118.0298	118.0298	0.0000	Trigonelline	$[M-H_2O-H]^-$	$C_7H_7NO_2$
119.0139	119.0138	0.5125	Salicylic acid	$[M-H_2O-H]^-$	$C_7H_6O_3$
119.0251	119.0251	-0.4957	Urocanic acid	$[M-H_2O-H]^-$	$C_6H_6N_2O_2$
120.0091	120.0092	-0.5416	6-Hydroxynicotinic acid (6-HNA)	$[M-H_2O-H]^-$	$C_6H_5NO_3$
121.0295	121.0296	-1.0989	Benzoic acid	$[M-H]^-$	$C_7H_6O_2$
121.0407	121.0408	-0.5618	Niacinamide	$[M-H]^-$	$C_6H_6N_2O$
122.0248	122.0248	0.0082	Nicotinic acid	$[M-H]^-$	$C_6H_5NO_2$
122.9854	122.9853	1.2684	Pyruvic acid	$[M+Cl]^-$	$C_3H_4O_3$
124.0074	124.0068	4.9997	Taurine	$[M-H]^-$	$C_2H_7NO_3S$
124.0171	124.0167	3.1566	L-Alanine	$[M+Cl]^-$	$C_3H_7NO_2$
124.0380	124.0377	2.7615	4-Aminobutyric acid (GABA)	$[M+Na-2H]^-$	$C_4H_9NO_2$
127.0013	127.0012	0.2967	Glyceric acid	$[M+Na-2H]^-$	$C_3H_6O_4$
127.0513	127.0515	-1.4482	Glutamine	$[M-H_2O-H]^-$	$C_5H_{10}N_2O_3$
128.0353	128.0353	0.1328	Glutamic acid	$[M-H_2O-H]^-$	$C_5H_9NO_4$
129.0921	129.0922	-0.3796	Heptanoic acid	$[M-H]^-$	$C_7H_{14}O_2$
130.0874	130.0874	-0.4535	Leucine/Isoleucine	$[M-H]^-$	$C_6H_{13}NO_2$
131.0139	131.0139	-0.3053	Phenylglyoxylic acid (PGOA)	$[M-H_2O-H]^-$	$C_8H_6O_3$
131.0350	131.0350	-0.4732	D-Ribose	$[M-H_2O-H]^-$	$C_5H_{10}O_5$
133.0407	133.0408	-0.5637	N1-Methyl-4-pyridone-3-carboxamide (4PY)	$[M-H_2O-H]^-$	$C_7H_8N_2O_2$

134.0248	134.0247	0.4850	3-Hydroxyanthranilic acid (3-HAA)	$[M-H_2O-H]^-$	$C_7H_7NO_3$
135.0452	135.0454	-2.1326	Phenylacetic acid (PAA)	$[M-H]^-$	$C_8H_8O_2$
135.0564	135.0565	-0.8811	N-Methylnicotinamide (NMN)	$[M-H]^-$	$C_7H_8N_2O$
136.0404	136.0405	-0.4557	2-Aminobenzoic acid (2-ABA)	$[M-H]^-$	$C_7H_7NO_2$
137.0244	137.0244	0.0803	Salicylic acid	$[M-H]^-$	$C_7H_6O_3$
137.0357	137.0357	-0.6933	Urocanic acid	$[M-H]^-$	$C_6H_6N_2O_2$
138.0197	138.0199	-1.5722	6-Hydroxynicotinic acid (6-HNA)	$[M-H]^-$	$C_6H_5NO_3$
139.0013	139.0012	0.2999	Succinic acid	$[M+Na-2H]^-$	$C_4H_6O_4$
139.0769	139.0765	3.1499	Choline	$[M+Cl]^-$	$C_5H_{14}NO$
143.1078	143.1078	-0.4123	Caprylic acid	$[M-H]^-$	$C_8H_{16}O_2$
145.0506	145.0506	0.2620	L-Fucose	$[M-H_2O-H]^-$	$C_6H_{12}O_5$
145.0619	145.0619	-0.0207	Glutamine	$[M-H]^-$	$C_5H_{10}N_2O_3$
145.0983	145.0983	-0.2068	Lysine	$[M-H]^-$	$C_6H_{14}N_2O_2$
148.0404	148.0403	0.4053	Pyridoxal	$[M-H_2O-H]^-$	$C_8H_9NO_3$
150.0561	150.0561	-0.4465	Norepinephrine	$[M-H_2O-H]^-$	$C_8H_{11}NO_3$
151.0401	151.0400	0.3310	p-Hydroxyphenylacetic acid (p-HPAA)	$[M-H]^-$	$C_8H_8O_3$
151.0513	151.0514	-0.3774	N1-Methyl-4-pyridone-3-carboxamide (4PY)	$[M-H]^-$	$C_7H_8N_2O_2$
152.0119	152.0118	0.9245	Proline	$[M+K-2H]^-$	$C_5H_9NO_2$
152.0353	152.0354	-0.7696	3-Hydroxyanthranilic acid (3-HAA)	$[M-H]^-$	$C_7H_7NO_3$

153.0169	153.0169	0.0506	Glutaric acid	$[M+Na-2H]^-$	$C_5H_8O_4$
153.0193	153.0195	-1.0195	Gentisic acid	$[M-H]^-$	$C_7H_6O_4$
154.0065	154.0060	2.9220	Indole	$[M+K-2H]^-$	C_8H_7N
154.0122	154.0122	-0.4693	L-Aspartic acid	$[M+Na-2H]^-$	$C_4H_7NO_4$
154.0622	154.0619	2.0706	Histidine	$[M-H]^-$	$C_6H_9N_3O_2$
155.0714	155.0715	-0.8964	Suberic acid	$[M-H_2O-H]^-$	$C_8H_{14}O_4$
157.1234	157.1235	-0.4837	Pelargonic acid	$[M-H]^-$	$C_9H_{18}O_2$
159.0234	159.0240	-3.7667	Cysteinylglycine	$[M-H_2O-H]^-$	$C_5H_{10}N_2O_3S$
161.0357	161.0358	-0.6272	Nicotinuric acid	$[M-H_2O-H]^-$	$C_8H_8N_2O_3$
161.0455	161.0455	0.5216	D-Glucose	$[M-H_2O-H]^-$	$C_6H_{12}O_6$
163.0401	163.0404	-1.7910	Homovanillic acid	$[M-H_2O-H]^-$	$C_9H_{10}O_4$
163.0612	163.0613	-0.5581	L-Fucose	$[M-H]^-$	$C_6H_{12}O_5$
164.0353	164.0353	0.1890	4-Pyridoxic acid	$[M-H_2O-H]^-$	$C_8H_9NO_4$
164.0717	164.0719	-1.0057	Phenylalanine	$[M-H]^-$	$C_9H_{11}NO_2$
166.0510	166.0511	-0.6203	Pyridoxal	$[M-H]^-$	$C_8H_9NO_3$
167.0211	167.0213	-1.1555	Uric acid	$[M-H]^-$	$C_5H_4N_4O_3$
167.0826	167.0828	-1.2150	Pyridoxamine	$[M-H]^-$	$C_8H_{12}N_2O_2$
168.0666	168.0666	-0.1250	Norepinephrine	$[M-H]^-$	$C_8H_{11}NO_3$
169.0870	169.0872	-1.2124	Azelaic acid	$[M-H_2O-H]^-$	$C_9H_{16}O_4$
171.0275	171.0273	0.8562	D-Ribose	$[M+Na-2H]^-$	$C_5H_{10}O_5$
171.1391	171.1390	0.0993	Capric acid	$[M-H]^-$	$C_{10}H_{20}O_2$
172.0404	172.0404	0.0698	5-Hydroxyindoleacetic acid (5-HIAA)	$[M-H_2O-H]^-$	$C_{10}H_9NO_3$
173.1044	173.1043	0.7625	L-Arginine	$[M-H]^-$	$C_6H_{14}N_4O_2$

174.0201	174.0199	1.3442	1-Methylnicotinamide (1-MNA)	$[M+K-2H]^-$	$C_7H_9N_2O$
179.0462	179.0464	-0.9662	Nicotinuric acid	$[M-H]^-$	$C_8H_8N_2O_3$
179.0561	179.0563	-1.3069	D-Glucose	$[M-H]^-$	$C_6H_{12}O_6$
180.0666	180.0665	0.6886	L-Tyrosine	$[M-H]^-$	$C_9H_{11}NO_3$
183.0121	183.0117	2.6610	4-Ethylphenylsulfate (4-EPS)	$[M-H_2O-H]^-$	$C_8H_{10}O_4S$
183.1027	183.1027	-0.1147	Sebacic acid	$[M-H_2O-H]^-$	$C_{10}H_{18}O_4$
185.9997	185.9988	4.3887	Methionine	$[M+K-2H]^-$	$C_5H_{11}NO_2S$
186.0206	186.0198	4.6306	Methionine sulfoxide (MetSO)	$[M+Na-2H]^-$	$C_5H_{11}NO_3S$
186.0561	186.0559	0.6127	Indolelactic acid	$[M-H_2O-H]^-$	$C_{11}H_{11}NO_3$
187.0976	187.0975	0.7109	Azelaic acid	$[M-H]^-$	$C_9H_{16}O_4$
188.0353	188.0355	-0.9573	Kynurenic acid	$[M-H]^-$	$C_{10}H_7NO_3$
189.0670	189.0672	-1.1795	Kynurenine	$[M-H_2O-H]^-$	$C_{10}H_{12}N_2O_3$
196.0227	196.0227	0.0480	N-Acetyl-L-aspartic acid (NAA)	$[M+Na-2H]^-$	$C_6H_9NO_5$
197.0390	197.0396	-2.8015	Bisnorbiotin	$[M-H_2O-H]^-$	$C_8H_{12}N_2O_3S$
199.0877	199.0879	-0.9041	N-Acetylserotonin	$[M-H_2O-H]^-$	$C_{12}H_{14}N_2O_2$
199.1704	199.1705	-0.6176	Dodecanoic acid	$[M-H]^-$	$C_{12}H_{24}O_2$
201.0381	201.0382	-0.5814	D-Glucose	$[M+Na-2H]^-$	$C_6H_{12}O_6$
201.0670	201.0669	0.4924	5-Hydroxy-L-tryptophan (5-HTP)	$[M-H_2O-H]^-$	$C_{11}H_{12}N_2O_3$
211.1340	211.1340	-0.1137	Dodecanedioic acid (DDDA)	$[M-H_2O-H]^-$	$C_{12}H_{22}O_4$
213.1860	213.1861	-0.4175	Tridecanoic acid	$[M-H]^-$	$C_{13}H_{26}O_2$

215.0328	215.0332	-1.7160	D-Glucose	$[M+Cl]^-$	$C_6H_{12}O_6$
227.2017	227.2015	0.6910	Myristic acid	$[M-H]^-$	$C_{14}H_{28}O_2$
230.0129	230.0120	3.7302	Norepinephrine sulfate (NE-S)	$[M-H_2O-H]^-$	$C_8H_{11}NO_6S$
231.1754	231.1758	-1.3453	Hexadecatetraenoic acid	$[M-H_2O-H]^-$	$C_{16}H_{26}O_2$
232.0285	232.0275	4.2710	Dopamine 3-O-sulfate (DA-3S)	$[M-H]^-$	$C_8H_{11}NO_5S$
236.9994	236.9986	3.0927	4-Ethylphenylsulfate (4-EPS)	$[M+Cl]^-$	$C_8H_{10}O_4S$
239.0593	239.0598	-2.3315	L-Tryptophan	$[M+Cl]^-$	$C_{11}H_{12}N_2O_2$
241.2173	241.2175	-0.8499	Pentadecanoic acid	$[M-H]^-$	$C_{15}H_{30}O_2$
252.0103	252.0092	4.1448	Tyramine-O-sulfate	$[M+Cl]^-$	$C_8H_{11}NO_4S$
253.2173	253.2177	-1.4928	Palmitoleic acid	$[M-H]^-$	$C_{16}H_{30}O_2$
255.2330	255.2328	0.4114	Palmitic acid	$[M-H]^-$	$C_{16}H_{32}O_2$
269.2486	269.2484	0.8357	Heptadecanoic acid	$[M-H]^-$	$C_{17}H_{34}O_2$
279.2330	279.2329	0.1862	Linoleic acid	$[M-H]^-$	$C_{18}H_{32}O_2$
281.2486	281.2485	0.3164	Oleic acid	$[M-H]^-$	$C_{18}H_{34}O_2$
283.2643	283.2642	0.3530	Stearic acid	$[M-H]^-$	$C_{18}H_{36}O_2$
303.2330	303.2329	0.3100	Arachidonic acid	$[M-H]^-$	$C_{20}H_{32}O_2$
347.2228	347.2211	4.7635	Tetrahydrocortisol	$[M-H_2O-H]^-$	$C_{21}H_{34}O_5$
360.0005	359.9999	1.6582	Cytidine monophosphate (CMP)	$[M+K-2H]^-$	$C_9H_{14}N_3O_8P$
384.0003	384.0019	-4.1302	Cytidine Diphosphate (CDP)	$[M-H_2O-H]^-$	$C_9H_{15}N_3O_{11}P_2$
